# The role of *Bacillus* species in the management of plant-parasitic nematodes

**DOI:** 10.3389/fmicb.2024.1510036

**Published:** 2025-01-17

**Authors:** Prabhakaran Vasantha-Srinivasan, Ki Beom Park, Kil Yong Kim, Woo-Jin Jung, Yeon Soo Han

**Affiliations:** ^1^Department of Applied Biology, Institute of Environmentally Friendly Agriculture (IEFA), College of Agriculture and Life Sciences, Chonnam National University, Gwangju, Republic of Korea; ^2^Research and Development Center, Invirustech Co., Inc., Gwangju, Republic of Korea; ^3^Department of Agricultural Chemistry, Institute of Environmentally-Friendly Agriculture (IEFA), College of Agriculture and Life Sciences, Chonnam National University, Gwangju, Republic of Korea

**Keywords:** plant-parasitic nematodes, biocontrol, *Bacillus* spp., nematicidal compounds, integrated pest management

## Abstract

Plant-parasitic nematodes (PPNs), including root-knot nematodes (*Meloidogyne* spp.), cyst nematodes (*Heterodera* and *Globodera* spp.), and other economically significant nematode species, pose severe threats to global agriculture. These nematodes employ diverse survival strategies, such as dormancy in cysts or robust infective juvenile stages. Consequently, their management is challenging. Traditional control methods, such as the use of chemical nematicides, are increasingly scrutinized because of environmental and health concerns. This review focuses on the specific mechanisms employed by *Bacillus* spp., including nematicidal compound production, systemic resistance induction, and cuticle degradation, to target root-knot and cyst nematodes. These mechanisms offer sustainable solutions for managing nematodes and promoting soil health by enhancing microbial diversity and nutrient cycling. An integrated approach leveraging *Bacillus-*based biocontrol is proposed to maximize efficacy and agricultural sustainability.

## Introduction

### Overview of nematode infestations in crops and their impact on agriculture

Nematode infestations significantly threaten global agriculture, causing substantial economic losses of over USD 80 billion annually (Nicol et al., 2011; Abd-Elgawad, 2024). Plant-parasitic nematodes (PPNs) are highly diverse and include various species, such as root-knot nematodes (*Meloidogyne* spp.), cyst nematodes (*Heterodera* and *Globodera* spp.), lesion nematodes (*Pratylenchus* spp.), and reniform nematodes (*Rotylenchulus reniformis*). These nematodes exhibit unique parasitic mechanisms. Hence, their management in agricultural systems is challenging.

Root-knot nematodes invade root tissues and induce the formation of specialized feeding structures called giant cells, diverting host resources and stunting plant growth. *Bacillus subtilis* produces nematicidal enzymes, such as proteases, which degrade nematode cuticles, reducing mobility and infectivity. Secondary metabolites, such as fengycin and surfactin, exhibit potent activity by disrupting nematode cell membranes, causing cell lysis and death (Jiang et al., 2021). Moreover, these metabolites inhibit egg hatching and juvenile development, effectively suppressing the nematode life cycle. On the other hand, cyst nematodes form syncytia in root tissues, resulting in long-term nutrient extraction. Lesion nematodes produce migratory lesions that compromise root integrity and increase susceptibility to secondary infections (Gupta et al., 2023). These adaptations result in yield losses, with root-knot nematodes alone accounting for an estimated loss of over 5% globally. Their adaptability and multiple life cycles in warm climates exacerbate this damage (Subbotin et al., 2021). Similarly, cyst nematodes survive under unfavorable conditions by forming resilient cysts containing eggs, enabling extended dormancy in the soil (Moens et al., 2018). *B. amyloliquefaciens* plays a crucial role in managing cyst nematodes by inducing systemic resistance in plants, thereby suppressing the formation of syncytia within root tissues. This bacterium also produces chitinases to degrade cyst shells, preventing hatching and subsequent infestations (Ngalimat et al., 2021). Given these functions of *Bacillus* spp. and their role in improving plant vigor, they are effective against cyst nematodes in diverse agricultural systems.

The survival strategies of nematodes demand tailored management approaches that account for the distinct biological traits of each group. For instance, root-knot nematodes secrete effector proteins that suppress key host plant defense pathways, such as those mediated by jasmonic acid (JA) and salicylic acid (SA), while cyst nematodes release effector proteins that alter root architecture to facilitate syncytium formation (Ahmad et al., 2021). Moreover, lesion nematodes disrupt cell walls enzymatically, contributing to extensive root decay. Understanding these intricate molecular interactions is crucial for devising effective and sustainable management strategies.

Traditional control methods, such as crop rotation, the use of resistant cultivars, and the use of chemical nematicides, are limited by the biological versatility of nematodes and the environmental concerns associated with chemical usage. The ability of root-knot nematodes to overcome resistant cultivars further complicates breeding efforts (Pradhan et al., 2023). Moreover, although chemical nematicides are initially effective, they pose risks to nontarget organisms and contribute to environmental degradation (Kumar et al., 2017). These limitations underscore the need for safer, eco-friendly alternatives.

Recent advances in biocontrol have demonstrated the potential of *Bacillus* spp. in combating specific PPNs. *Bacillus* spp. employ various mechanisms, such as the production of nematicidal metabolites (e.g., lipopeptides and proteases), the induction of systemic resistance in plants, and competition with nematodes for resources (Patil et al., 2019; Jiang et al., 2021). For instance, *B. subtilis* produces fengycin and surfactin lipopeptides, which disrupt root-knot nematode cuticles, while *B. amyloliquefaciens* induces systemic resistance in plants, enhancing defenses against cyst nematodes (Lin et al., 2020). Understanding the mechanisms underlying these distinct interactions is crucial for optimizing their applications in nematode management programs and ensuring that they also contribute positively to soil health. This review emphasizes the targeted use of *Bacillus* spp. against root-knot and cyst nematodes, detailing their distinct survival strategies and biocontrol mechanisms.

Given the diversity of PPNs and the limitations of conventional management strategies, this review focuses on *Bacillus* spp. as biocontrol agents, discussing their mechanisms, efficacy, and potential for integration into sustainable nematode management programs. The discussion covers multiple PPNs, focusing on crop nematodes, especially root-knot, cyst, lesion, and reniform nematodes. The literature is sourced from reputable databases, including Elsevier, Springer, and MDPI, ensuring the inclusion of high-quality and relevant studies.

### Major phytopathogenic nematodes in global agriculture

Phytopathogenic nematodes pose a significant threat to global agriculture. They impact a wide range of crops by feeding on plant roots, disrupting nutrient uptake, and serving as vectors for other pathogens. The most harmful genera include *Meloidogyne*, *Heterodera*, *Globodera*, *Pratylenchus*, *Radopholus*, *Rotylenchulus*, *Ditylenchus*, and *Bursaphelenchus*, each exhibiting unique life cycles, modes of action, and seasonal habitats that contribute to pathogenicity (Mesa-Valle et al., 2020; Palomares-Rius et al., 2020).

Root-knot nematodes (*Meloidogyne* spp.), including *M. incognita*, *M. javanica*, and *M. arenaria*, are particularly damaging. Their life cycles progress from eggs to infective juveniles and adults, with juveniles primarily causing damage by penetrating plant roots. These nematodes thrive in warm climates and cause peak damage during spring and summer, contributing to significant yield losses in various crops, such as tomatoes, soybeans, and cotton in Brazil, China, and other regions (Blouin et al., 1998; Subbotin et al., 2021). Cyst nematodes (*Heterodera* and *Globodera* spp.) pose unique challenges because of their ability to form cysts containing eggs. Consequently, they can survive for long durations under adverse conditions. The soybean cyst nematode *H. glycines* and the golden potato cyst nematode *G. rostochiensis* cause substantial crop losses, particularly in temperate regions. Their dormant cysts hatch under favorable environmental conditions, typically in spring, aligning with the planting season (He et al., 2022). Lesion nematodes (*Pratylenchus* spp.) are migratory endoparasites that create lesions in root tissues as they feed, significantly impairing plant health. These nematodes are active throughout the year in warm, moist environments, such as those in tropical agricultural regions, causing severe yield losses in various crops, such as banana, coffee, and soybean (Saikai and MacGuidwin, 2022; Riascos-Ortiz et al., 2022). Similarly, burrowing nematodes (*Radopholus similis*) and stem nematodes (*Ditylenchus dipsaci*) exhibit seasonal activity, with the former thriving in wet tropical climates and the latter affecting bulbous plants in cooler climates (Mathew and Opperman, 2019; Sturhan and Brzeski, 2020). The global burden of these nematodes is substantial. Hence, there is an urgent need for sustainable, effective management strategies to mitigate the impact of these nematodes on global food security.

### Traditional methods of nematode control and their limitations

Traditional nematode management approaches, including cultural practices, biocontrol methods, and chemical treatments, have been widely implemented to mitigate the detrimental effects of nematodes and maintain crop health and productivity (Elango et al., 2020). Cultural methods, such as crop rotation, soil solarization, and sanitation, aim to interrupt the life cycle of nematodes, thereby diminishing their populations in the soil (Oka, 2010). Biocontrol methods leverage natural predators and antagonistic plants to maintain the ecological balance of nematode populations (El-Saadony et al., 2021). Chemical treatments, which involve the application of nematicides, can directly target nematodes and rapidly reduce their populations.

Despite their extensive use, these conventional methods have several limitations that undermine their long-term efficacy and sustainability (Sikora and Roberts, 2018). Although cultural practices, such as crop rotation, are theoretically effective, they require extensive knowledge and labor and can yield inconsistent results because of environmental variations (Grubišić et al., 2018). Biocontrol methods, including the use of antagonistic plants, such as marigold (*Tagetes* spp.) and neem (*Azadirachta indica*), offer environmentally friendly alternatives; however, they often fail to exhibit adequate suppressive effects and may require considerable time to be effective (Waller and Thamsborg, 2004). Moreover, the efficacy of biocontrol methods can significantly vary depending on the species involved and the environmental conditions.

Although chemical treatments provide rapid and effective nematode control, they pose significant risks to human health, nontarget organisms, and the environment. The persistent use of nematicides has led to the emergence of resistant nematode strains, thereby diminishing their long-term effectiveness (Timper, 2014). The regulatory restrictions posed on many effective nematicides because of their adverse environmental impacts have further limited their availability and use (Grubišić et al., 2018).

These inherent limitations of traditional nematode control methods highlight the need for innovative and sustainable approaches. Integrated pest management (IPM) strategies that combine traditional practices with modern technological advancements present a promising solution. These strategies aim to enhance the effectiveness of nematode control while minimizing the associated environmental and health risks.

### Biocontrol strategies for nematodes with a focus on *Bacillus* spp

Biocontrol strategies are being recognized as sustainable and environmentally friendly alternatives to chemical nematicides for managing nematode infestations. Various microbial agents and botanical extracts have shown potential for reducing nematode populations. For instance, fungal strains, such as *Auxarthron reticulatum* DY-2, *Verticillium saksenae* A-1, *Lecanicillium psalliotae* A-1, and *L. antillanum* B-3, have been explored for their effectiveness in parasitizing and reducing nematode populations (Oh et al., 2014a,b; Nguyen et al., 2014). Additionally, extracts of *Cinnamomum cassia* bark and *C. aromaticum* have demonstrated enzyme-inhibitory and nematicidal properties, thereby serving as potential agents for botanical interventions (Nguyen et al., 2009, 2012; Nguyen and Jung, 2014). Nguyen et al. (2011) demonstrated that treatment with *C. cassia* crude extracts significantly reduced gall formation and nematode growth in a dose-dependent manner in root-knot nematode-infested cucumber plants. This treatment also enhanced the activities of antioxidative enzymes, such as SOD, CAT, and APX, in cucumber leaves, indicating a strengthened defense response against the nematode. Furthermore, bark extracts of *Terminalia nigrovenulosa* and related compounds have been found to disrupt nematode life cycles (Seo et al., 2013).

**Figure 1:**
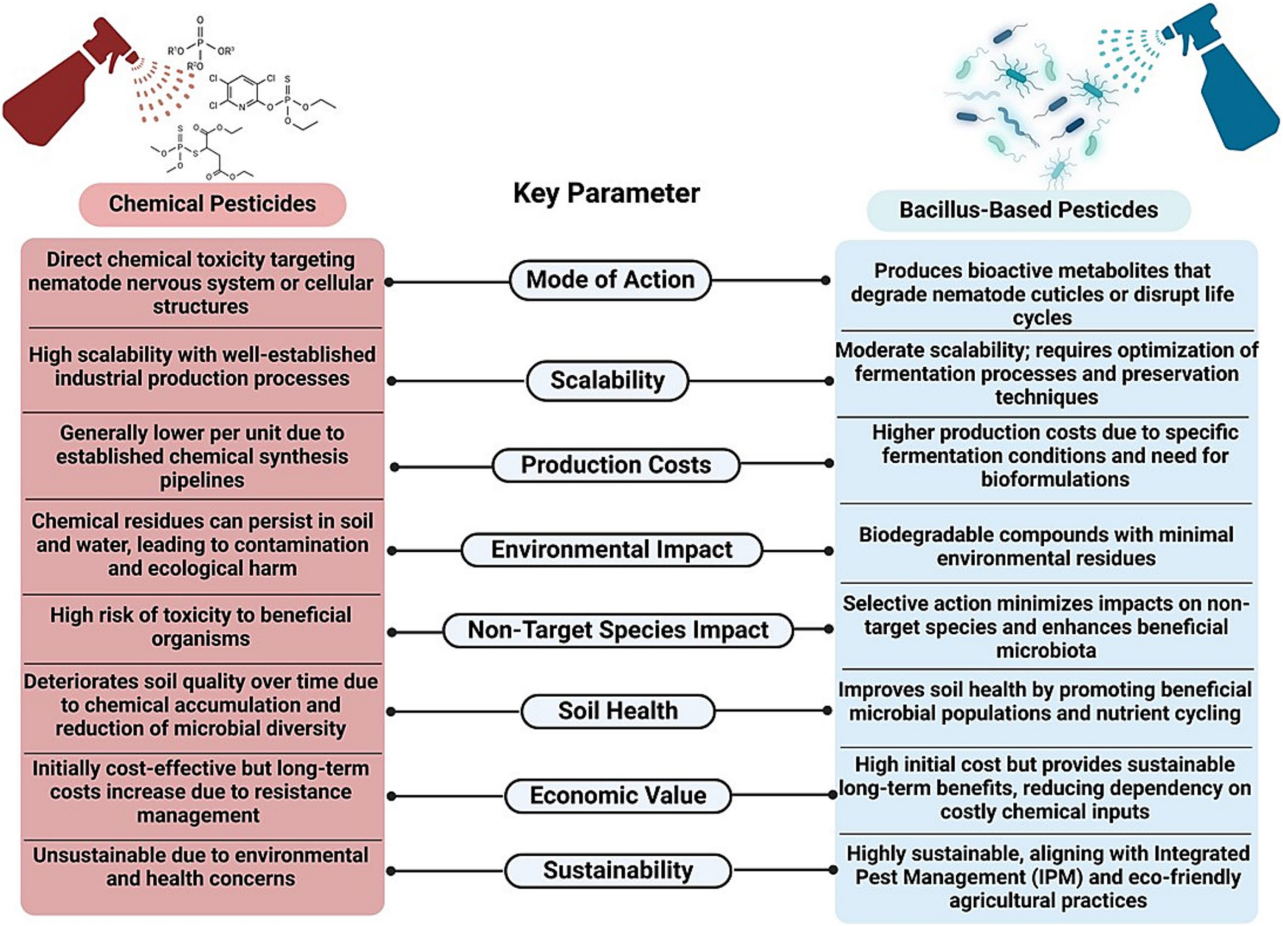
Schematic representation of comparison of conventional and *Bacillus*-based nematode management strategies.

In addition to fungi and botanical extracts, entomopathogenic nematodes (EPNs), such as *Steinernema* and *Heterorhabditis* spp., are known for their ability to release symbiotic bacteria (e.g., *Xenorhabdus* and *Photorhabdus* spp.) that produce toxins lethal to nematodes (El Aimani et al., 2022). Furthermore, predatory fungi, such as *Paecilomyces* and *Arthrobotrys* spp., can trap and digest nematodes, while endophytic fungi, such as *Trichoderma* spp., can colonize plant roots and produce enzymes and metabolites that can inhibit nematode activity and enhance plant resistance (Singh et al., 2019). The incorporation of organic amendments, such as compost and green manure, into the soil can also boost the populations of beneficial microbes that compete with or directly antagonize nematodes. These biocontrol strategies can not only reduce the reliance on chemical nematicides but also promote sustainable agricultural practices by enhancing soil health and biodiversity. The schematic representation of comparison of chemical pesticide-based nematode management with *Bacillus*-based biocontrol approaches, showcasing differences in mode of action, scalability, production costs, environmental impacts, non-target species effects, soil health, economic value, and sustainability was displayed (Figure 1).

Bacterial antagonists are among the most promising biocontrol agents. They suppress PPNs through multiple mechanisms, including the production of nematicidal lipopeptides, such as surfactin and fengycin, which disrupt nematode cuticles and membranes. *Bacillus* spp. produce various enzymes, such as chitinases and proteases, which degrade nematode eggshells and cuticles, effectively reducing juvenile development and reproduction (Yang et al., 2013). In particular, *B. subtilis* triggers systemic resistance in plants by activating JA and SA signaling pathways, thereby enhancing the natural defenses of plants against nematode attacks (Chowdhury et al., 2015). The antagonistic effects of *Paenibacillus elgii* HOA73 and *P. illinoisensis* KJA-424 were evaluated through *in vitro* nematicidal assays and greenhouse experiments. Key methodologies included assessing nematode motility and mortality using bacterial supernatants and evaluating the activity of enzymes, such as chitinases and proteases. Greenhouse trials confirmed reductions in nematode gall formation and reproduction in infested tomato plants (Jung et al., 2002; Nguyen et al., 2013). *Bacillus* spp., in particular, are a diverse group of gram-positive, rod-shaped, endospore-forming bacteria commonly found in soil and plant environments. They can produce various bioactive compounds, including enzymes, antibiotics, and toxins, which enhance their effectiveness in controlling plant pathogens and promoting plant health (El Aimani et al., 2022). Some *Bacillus* spp. are notably effective against nematodes and other plant pathogens, making them valuable for sustainable agricultural practices.

*Bacillus* spp. produce various nematicidal compounds, including lipopeptides, proteases, and chitinases, which target nematodes at various life stages (Tran et al., 2019). These soil-dwelling bacteria produce spores that can endure extreme environmental conditions, making them ideal candidates for sustainable nematode management (Singh et al., 2019). They can directly antagonize nematodes by producing toxins, enzymes, and other bioactive compounds that impact nematode mobility, development, and reproduction (Migunova and Sasanelli, 2021). *Bacillus* spp., such as *B. thuringiensis* (Bt) and *B. firmus*, have been extensively studied for their nematicidal activities (Zuckerman et al., 1993). For instance, Bt produces crystal (Cry) proteins that are toxic to a broad range of nematodes and can cause cell lysis and death upon ingestion (Forghani and Hajihassani, 2020). Similarly, *B. firmus* produces enzymes and secondary metabolites that degrade the nematode cuticle and interfere with physiological processes. The use of *Bacillus* spp. not only reduces the reliance on chemical nematicides, thereby mitigating environmental impacts, but also promotes soil health by maintaining beneficial microbial populations (Tran et al., 2019).

*Bacillus* spp. can effectively manage PPN infestations through various biocontrol strategies (Tian et al., 2007; Gamalero and Glick, 2020; Diyapoglu et al., 2022). The nematicidal activity of *B. subtilis* was assessed through *in vitro* bioassays focusing on lipopeptides, such as surfactin and fengycin, which can cause significant disruption of nematode cell membranes, resulting in mortality (El Aimani et al., 2022). Similarly, studies on *B. amyloliquefaciens* have revealed its efficacy in IPM programs. By producing antifungal and antibacterial metabolites, the bacterium could exhibit dual efficacy against PPNs and secondary infections in plants under controlled and field conditions (Cetintas et al., 2018). These strategies highlight the versatility of *Bacillus* spp. as biocontrol agents through multiple mechanisms, including direct toxicity, the inhibition of nematode development, and the enhancement of plant resistance. These bacteria also induce systemic resistance in plants, enhancing their defensive capabilities against nematode attacks (Yang et al., 2022). They produce chitinase and other enzymes that can degrade nematode eggshells, thereby reducing hatching rates and subsequent infection levels. Field trials have also revealed that formulations containing *Bacillus* spp. can significantly reduce root galling and improve plant health, demonstrating their practical applicability in agricultural settings (Forghani and Hajihassani, 2020).

**Figure 2:**
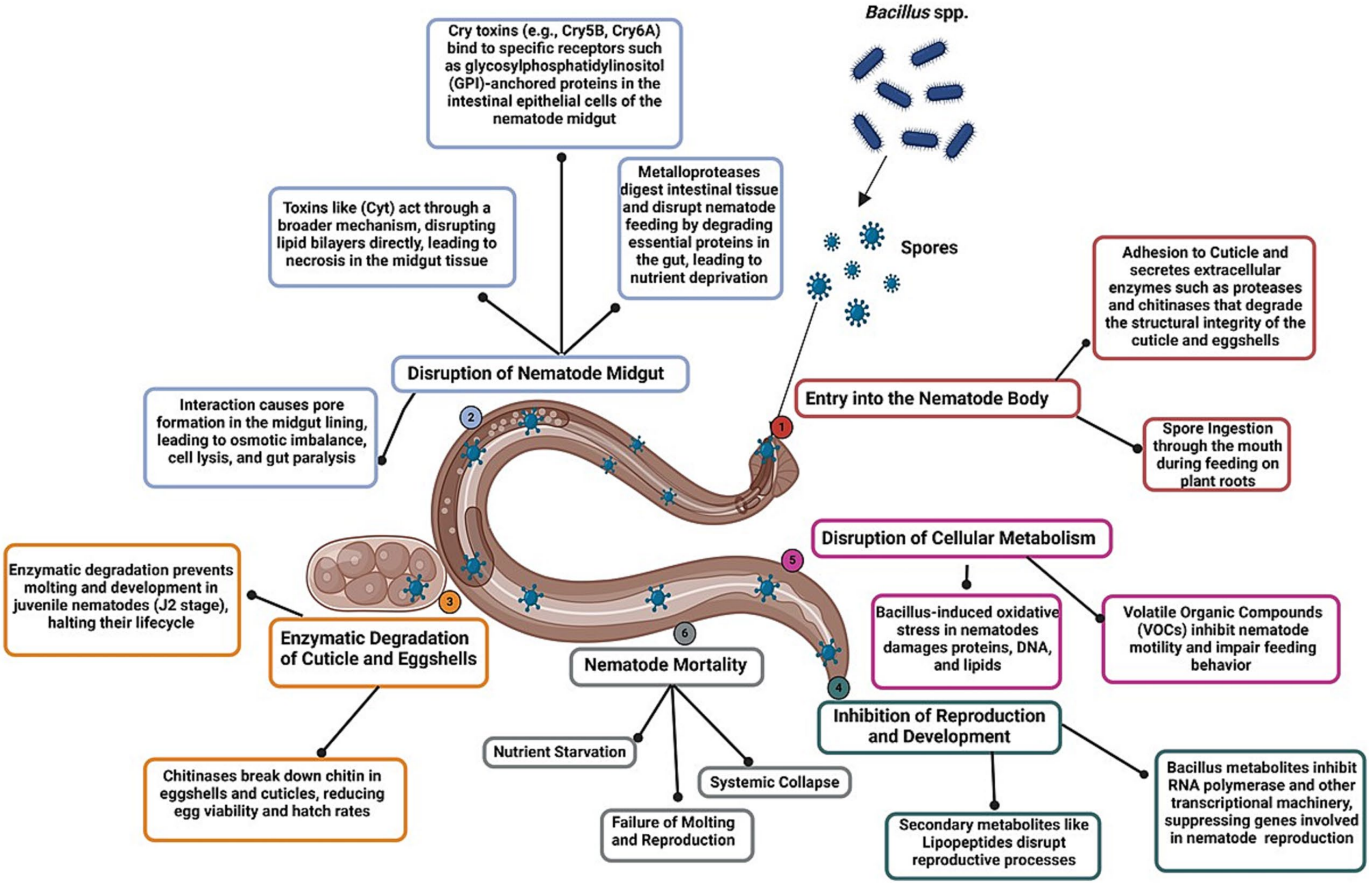
Mode of action of *Bacillus* spp. against plant-parasitic nematodes. The figure illustrates the sequential mechanisms of *Bacillus* species, including the entry of spores into the nematode body via ingestion or adhesion to the cuticle, enzymatic degradation of structural components (such as cuticles and eggshells), and disruption of intestinal cells through Cry and Cyt toxin-induced pore formation. The figure also highlights the inhibition of nematode reproduction, the disruption of cellular metabolism, and systemic physiological collapse, ultimately resulting in nematode mortality.

In summary, *Bacillus* spp. employ various proteins and secondary metabolites to exhibit nematicidal effects. The key proteins include Cry proteins from Bt, which act by forming pores in the gut cells of nematodes, causing cell lysis and death (Forghani and Hajihassani, 2020; Diyapoglu et al., 2022). *B. firmus* produces chitinase, an enzyme that breaks down chitin in nematode eggshells, thereby preventing hatching and reducing nematode populations (Tran et al., 2019). Additionally, *B. subtilis* produces lipopeptides, such as surfactin and fengycin, which disrupt nematode cell membranes, causing the loss of cell integrity and cell death (El Aimani et al., 2022). *B. amyloliquefaciens* produces proteases, which degrade nematode cuticles and interfere with their physiological processes, resulting in reduced viability and reproduction (Cetintas et al., 2018). The primary modes of action through which *Bacillus* spp. target nematodes include direct toxicity by producing toxins and enzymes, the inhibition of egg hatching and juvenile development, the induction of systemic resistance in plants, and the disruption of physiological processes by degrading structural components (e.g., cuticles) and interfering with metabolic pathways essential for nematode survival (Shafi et al., 2017). The detailed mechanisms of action underlying the efficacy of *Bacillus* spp. against PPNs are presented in Figure 2.

## Historical perspective on the use of *Bacillus* spp. as biocontrol agents

The historical development of *Bacillus* spp. as biocontrol agents against plant pathogens, particularly nematodes, highlights significant advancements in scientific understanding and practical applications. *Bacillus* spp. were first identified by Ferdinand Cohn in the late 19th century. Early research highlighted their roles in improving soil health and promoting plant growth through the production of nematicidal compounds, such as enzymes and secondary metabolites (Brzezinska et al., 2020).

**Figure 3:**
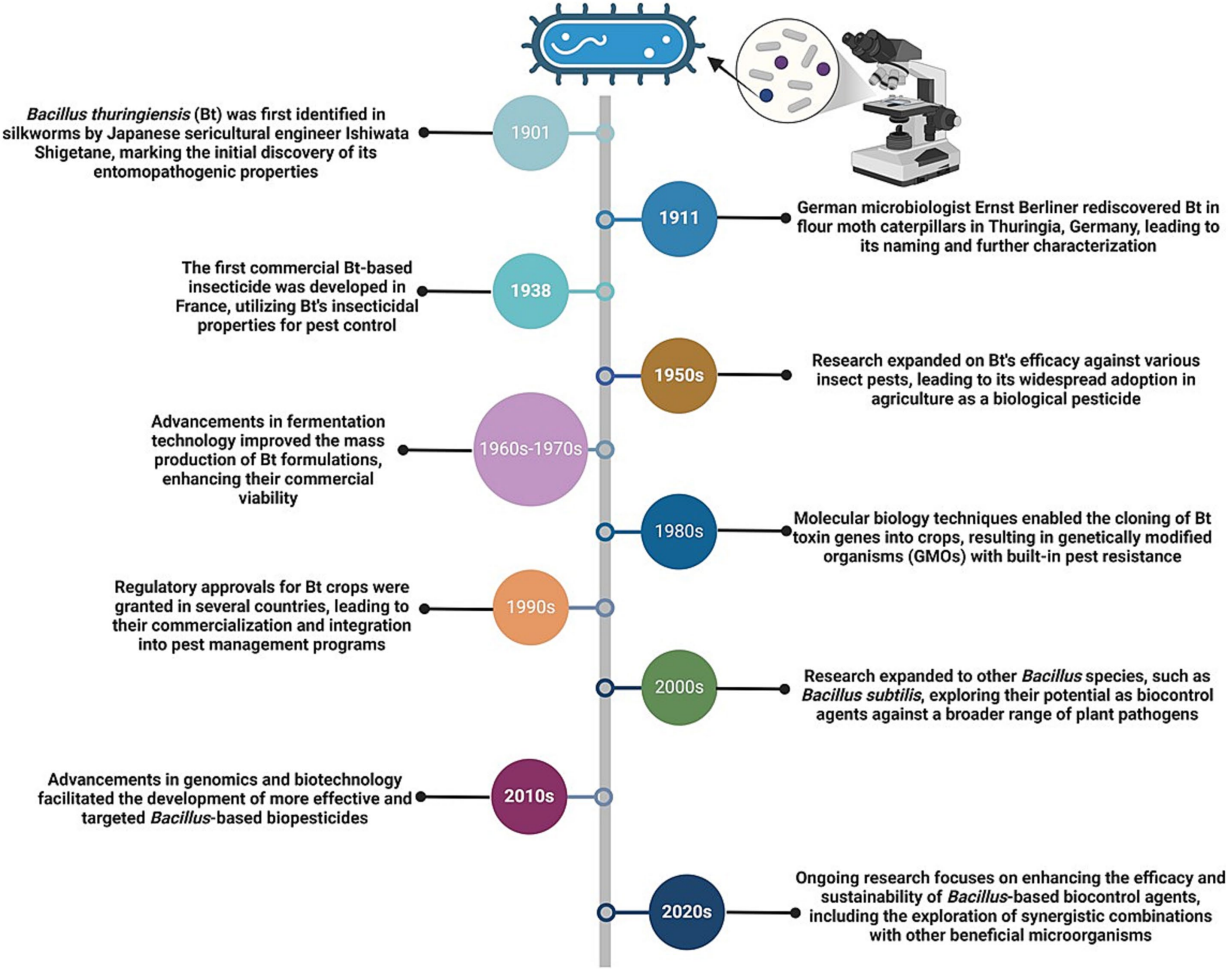
Timeline of *Bacillus* species development as biocontrol agents. This timeline highlights significant milestones in the development of Bacillus species as biocontrol agents, from their initial discovery to advancements in genetic engineering and sustainable agricultural practices.

The mid-20th century marked a pivotal advancement with the discovery of Bt and its insecticidal Cry proteins, forming the foundation for experimental biocontrol applications (Sanahuja et al., 2011). A timeline highlighting significant milestones in the development of *Bacillus* species as biocontrol agents, from their initial discovery to advancements in genetic engineering and sustainable agricultural practices, emphasizing their expanding role in integrated pest management, is presented (Figure 3). Initial studies on nematode management focused on nematicidal compounds, such as chitinases and proteases, (Bacon et al., 2006). By the 1970s and 1980s, researchers identified specific toxins and enzymes produced by *Bacillus* spp., revealing their targeted actions against nematodes (Van Frankenhuyzen, 2009, 2013). Field trials in the 1990s evaluated the efficacy of *Bacillus*-based biocontrol agents under various environmental and agronomic conditions. These studies highlighted the importance of application methods, soil properties, and microbial interactions in achieving consistent nematode suppression (Etesami et al., 2023; Serrão et al., 2024). With advancements in genomic technologies, researchers unraveled genes and regulatory pathways responsible for the biocontrol properties of *Bacillus* spp. in the early 21st century. This enabled the development of genetically enhanced strains with improved efficacy and environmental resilience (Carmona-Hernandez et al., 2019). Given the commercial success of *Bacillus*-based products, these biocontrol agents were further integrated into IPM systems, offering sustainable alternatives to chemical nematicides (Castillo et al., 2013). Current research underscores the role of *Bacillus* spp. in promoting soil biodiversity and enhancing plant microbiomes, which contribute to long-term nematode suppression (Calvo et al., 2010). Biotechnological advances, including CRISPR and synthetic biology, have further expanded the potential of *Bacillus* spp., enhancing their stability, specificity, and ability to produce nematicidal compounds (Baptista et al., 2022). Key *Bacillus* spp., including Bt, *B. subtilis*, and *B. cereus*, are crucial because they produce diverse nematicidal compounds, such as Cry proteins, chitinases, and lipopeptides, which exhibit broad-spectrum activity against nematodes (Jouzani et al., 2017; Saxena et al., 2020; Ahmad et al., 2021). Comparative studies have demonstrated the unique strengths of *Bacillus* spp., providing insights into their compatibility with specific crops and soil environments. For example, *B. subtilis* induces systemic resistance in plants, Bt acts through direct toxin-mediated gut disruption, and *B. cereus* enhances soil health through microbial synergism (Diyapoglu et al., 2022; Tran et al., 2019). This historical trajectory highlights the evolution of *Bacillus* spp. from their initial discovery to becoming cornerstones of sustainable agriculture. The roles of *Bacillus* spp. in nematode biocontrol highlight their potential as integral components of IPM strategies, addressing key challenges in plant health management (Sanahuja et al., 2011; Raymond and Federici, 2017).

### Key *Bacillus* spp. and their efficacy against nematodes Bt

Bt is widely recognized for its potent nematicidal activity, primarily mediated by the production of insecticidal Cry and cytolytic (Cyt) proteins. These proteins, synthesized as protoxins during sporulation, exhibit significant efficacy against various PPNs, including *Meloidogyne* and *Heterodera* spp. (Verduzco-Rosas et al., 2021; Kahn et al., 2021). Experimental studies on the efficacy of Bt toxins generally utilize nematode bioassays, in which second-stage juveniles (J2) of *Meloidogyne* spp. are exposed to varying concentrations of Cry and Cyt proteins under controlled environmental conditions. Mortality, hatching inhibition, and mobility reduction are the commonly measured endpoints in such studies. Upon ingestion, the alkaline gut environment of nematodes solubilizes these protoxins, which are then activated by specific gut proteases. The activated Cry proteins bind to gut epithelial receptors, such as cadherin-like proteins, aminopeptidases, and alkaline phosphatases, inducing structural changes that facilitate membrane insertion and pore formation (Griffitts et al., 2005; Schnepf et al., 1998). This pore formation disrupts osmotic balance, causing cell lysis, gut paralysis, and eventual nematode death due to starvation or secondary infections (Bravo et al., 2007). Cyt proteins complement Cry proteins by targeting the lipid components of nematode cell membranes, thereby inducing cell lysis through distinct pore-forming mechanisms (Gill et al., 1992; Wei et al., 2003). In laboratory assays, Cry5B has been found to interact with glycosylphosphatidylinositol-anchored proteins in the gut cells of *M. incognita*, causing cell swelling and epithelial rupture. Cry6A specifically targets aspartyl protease and alkaline phosphatase receptors, initiating apoptosis and disrupting gut integrity (Barros dos Santos et al., 2022; Shi et al., 2020). These experiments typically involve histological analysis of nematode midgut tissues and the use of advanced imaging techniques to confirm receptor interactions and cellular damage. The specificity and effectiveness of Bt toxins vary among nematode species because of differences in gut receptor structures and proteolytic activation. Nematodes can use innate defenses, such as enzyme detoxification and pH modulation, to mitigate Bt toxicity, highlighting the complexity of host–pathogen interactions (Zhang et al., 2012). These interactions underscore the versatility and adaptability of Bt in managing diverse nematode infestations. Advances in molecular biology have facilitated the engineering of transgenic crops expressing Cry proteins, conferring continuous protection against nematodes. For example, in field trials, transgenic rice expressing Cry6A exhibited significant resistance to *M. graminicola*, with the nematode populations decreasing by 80% and yield improving by 30% (Lilley et al., 2011; Berlitz et al., 2014). Such experiments typically involve randomized field plots, and the efficacy of treatments is compared with those of chemical nematicides and untreated controls. Nematode population dynamics and yield data are analyzed to assess efficacy. The integration of Bt formulations with organic amendments, such as chitin or neem extracts, can further enhance their efficacy through synergistic effects (Chen et al., 2000; Radwan, 2007). Field applications of Bt-based biopesticides can be evaluated using standardized protocols. For instance, Cry55A-containing formulations have shown notable efficacy in reducing *M. incognita* populations under greenhouse and field conditions, with Cry55A-treated soil exhibiting 70% lower nematode gall formation than untreated controls. These findings highlight the potential of Cry55A as a soil inoculant (Manivannan et al., 2019; Ramalakshmi et al., 2020). Innovative delivery systems, such as seed treatments and soil inoculants, ensure early and sustained activity throughout the growing season (Etesami et al., 2023). These advancements align with sustainable agricultural practices, offering an eco-friendly alternative to chemical nematicides (Hui et al., 2012; Chen et al., 2024). Given its robust mechanisms of action, adaptability to various nematode species, and compatibility with sustainable practices, Bt plays a crucial role in modern nematode management frameworks. Comparative insights across species and delivery systems underscore its effectiveness as a cornerstone of nematode biocontrol strategies.

### B. subtilis

*B. subtilis*, a versatile PGPR, exhibits remarkable efficacy against PPNs through diverse mechanisms. This bacterium produces lipopeptides, such as surfactins, fengycins, and iturins, which disrupt nematode cell membranes, causing cell lysis and death (Heerklotz and Seelig, 2007; Henry et al., 2011). *In vitro* studies can confirm these effects by exposing *Meloidogyne* juveniles to purified lipopeptides and assessing mortality through microscopic observations and viability staining. Additionally, *B. subtilis* secretes hydrolytic enzymes, such as chitinases and proteases, which degrade nematode eggshells and cuticles, thereby inhibiting juvenile emergence and reproduction (Hu et al., 2007; Huang et al., 2008). Enzymatic activity is often assessed using substrate degradation assays, in which enzymatic activity is correlated with nematode population decline. *B. subtilis* also induces systemic resistance in plants by activating JA and SA pathways, thereby enhancing the production of phenolics and defense proteins that limit nematode penetration (Adiwena et al., 2023). In greenhouse studies, RT-qPCR and phenolic quantification assays can be used to validate these responses. Volatile organic compounds (VOCs), such as 2,3-butanediol and acetoin, further suppress nematode motility and reproduction while promoting rhizosphere health (Henry et al., 2011). These VOCs can be identified through GC–MS analysis, and their inhibitory effects can be confirmed by performing bioassays. The applications of *B. subtilis* include seed treatments, soil drenching, and foliar sprays. Seed treatments ensure early root colonization, while soil drenching targets root zones for sustained nematode suppression. Foliar sprays activate induced systemic resistance (ISR) pathways, indirectly reducing nematode infestations (Barnawal et al., 2017; Basiouny and Abo-Zaid, 2018). In field trials, these methods can be assessed through randomized designs to monitor nematode levels and yield improvements. When integrated into IPM frameworks, *B. subtilis* performs synergistically with organic amendments and other biocontrol agents, enhancing efficacy and promoting soil health (Cavalcanti et al., 2024). These combined strategies can maximize nematicidal potential and support sustainable agriculture. The multifaceted actions of *B. subtilis* highlight its pivotal role in reducing nematode infestations and promoting eco-friendly pest management practices.

### B. cereus

*B. cereus* exhibits robust nematicidal activity against PPNs through diverse mechanisms. It secretes metalloproteinases, such as neutral protease (Npr) and bacillolysin (BlyA), which degrade nematode cuticle proteins, thereby causing structural collapse and death (Yin et al., 2021a,b; Kulkova et al., 2023). Enzyme assays have confirmed the degradation of nematode cuticles, correlating enzymatic activity with nematode mortality. Lipopeptides, such as surfactin and fengycin, disrupt nematode cell membranes via pore formation, causing cell leakage and lysis (Tong-Jian et al., 2013; Hu et al., 2020). Fluorescent dyes have been used to validate membrane disruption.

*B. cereus* also produces siderophores, such as bacillibactin, which can chelate iron, thereby depriving nematodes of essential nutrients (Köhl et al., 2019). Furthermore, they produce bacteriocins, such as cerein, which can act as antibiotics and target nematode cellular processes. Bioassays have confirmed nutrient depletion and reduced viability in treated nematodes. Nano-bioformulations have further improved the stability and bioavailability of these bioactive compounds, ensuring prolonged nematode suppression in diverse soils (Kumar et al., 2021). Field trials have highlighted their extended activity and reduced application frequencies. Optimized delivery methods include soil drenching, seed treatments, and foliar sprays. Soil drenching ensures uniform root-zone colonization, while seed treatments enable early protection during crucial growth stages (Ahmed et al., 2019). Randomized trials have revealed significant reductions in *M. incognita* populations and improvements in yield. When combined with mycorrhizal fungi, *B. cereus* exhibits synergistic effects, enhancing soil microbial diversity and plant resilience (Hu et al., 2017). Genetic engineering approaches, including CRISPR, are being used to enhance the production of bioactive compounds and target-specific nematicidal properties (Mohamed et al., 2021). Through its diverse mechanisms of action, including enzyme secretion, nutrient competition, and direct nematode disruption, *B. cereus* offers a sustainable biocontrol option for PPN management. Its integration into IPM strategies and compatibility with sustainable agriculture highlight its crucial role in reducing chemical nematicide usage while improving crop health and productivity.

### B. megaterium

*B. megaterium* is a robust biocontrol agent that has been proven to be effective against PPNs by producing various bioactive compounds and enzymes. It secretes proteases, such as neutral and serine proteases, which degrade structural proteins in nematode cuticles, causing severe damage and death (Padgham and Sikora, 2007). Lipopeptides, such as surfactin and iturin, disrupt nematode cell membranes through pore formation, causing cell leakage and lysis (Pueyo et al., 2009). Additionally, *B. megaterium* synthesizes siderophores, such as bacillibactin, which can chelate iron in the rhizosphere, thereby depriving nematodes of vital nutrients and suppressing their populations while promoting a balanced microbial community. These processes have been validated through enzyme assays, correlating siderophore activity with nematode suppression (Huang et al., 2010). Nano-bioformulations have further enhanced the stability and bioavailability of *B. megaterium* metabolites, ensuring prolonged nematode suppression and reduced application frequency (Kumar et al., 2021). Various application techniques, including soil drenching and seed treatments, have been optimized for efficient delivery. Soil drenching ensures deep root penetration, while seed treatments facilitate early root colonization, offering sustained protection during crucial growth stages (Padgham and Sikora, 2007; Raza et al., 2024). These strategies have been effective against root-knot nematodes, such as *M. incognita*, significantly improving plant health and yields in field trials (Mostafa et al., 2018). Genetic engineering approaches, such as the overexpression of genes responsible for lipopeptide synthesis and VOC production, have been employed to enhance nematicidal efficacy. These efforts have shown promise in increasing activity against nematodes while maintaining environmental safety (Grage et al., 2017; Hartz et al., 2021). Through its multifaceted nematicidal mechanisms, *B. megaterium* serves as an eco-friendly alternative to chemical nematicides. Its adaptability and integration into IPM strategies make it a cornerstone of sustainable pest management. It can support agricultural productivity while minimizing environmental impacts.

### B. pumilus

*B. pumilus* employs diverse nematicidal mechanisms, making it a powerful biocontrol agent against PPNs. It acts by secreting proteolytic enzymes, such as subtilisin, which can degrade nematode cuticle proteins, causing osmotic imbalance and eventual death (Ramezani Moghaddam et al., 2014). Lipopeptides, such as pumilacidin and bacilysin, disrupt nematode cell membranes and induce pore formation, ion leakage, and cytoplasmic efflux, thereby causing rapid cell lysis (Dobrzyński et al., 2023). *B. pumilus* also synthesizes siderophores, such as bacillibactin, which can chelate iron and other essential nutrients, depriving nematodes of crucial resources and fostering beneficial microbial competition in the rhizosphere (Lee et al., 2016). Additionally, *B. pumilus* produces antimicrobial compounds, including bacteriocins, which disrupt nematode metabolic pathways. A guanidine compound from *B. pumilus* strain LYMC-3 exhibited potent activity against *Bursaphelenchus xylophilus*; the LC_50_ values were 113.5 and 62.5 mg/L after 24 and 48 h, respectively, highlighting its targeted efficacy (Li et al., 2018). Nano-bioformulations have improved the stability and bioavailability of *B. pumilus* metabolites, ensuring consistent nematode suppression in different agricultural conditions (Mahmoud et al., 2016). *B. pumilus* differentiates itself by integrating siderophore-mediated nutrient deprivation with enzymatic and antimicrobial strategies, unlike Bt (which relies on Cry proteins) or *B. cereus* (which relies on lipopeptides). Its compatibility with agronomic practices, such as seed treatments and soil drenching, facilitates early root colonization and uniform metabolite distribution, enhancing field performance. Furthermore, its synergy with mycorrhizal fungi and other beneficial microbes enhances nutrient cycling and plant resilience, creating a holistic defense against nematodes (Carriel and Soto, 2022). Through its multifaceted actions and adaptability, *B. pumilus* exhibits significant potential for integration into IPM strategies. Further research on genetic optimization, delivery systems, and formulations is warranted to sustainably maximize its agricultural impact.

### B. licheniformis

*B. licheniformis* employs diverse mechanisms, including enzymatic degradation, antimicrobial activity, and soil microbiome modulation, to manage PPNs. Its nematicidal activity is attributed to the secretion of hydrolytic enzymes, such as proteases and chitinases, which target the cuticles and eggshells of nematodes, impairing their mobility, reproduction, and viability (Park et al., 2015). For example, strain MH48 effectively degrades nematode structures, particularly in *B. xylophilus* (Jeong et al., 2015). Additionally, *B. licheniformis* produces lipopeptides, such as bacillomycin and fengycin, which disrupt nematode and fungal cell membranes, causing ion leakage and cytoplasmic loss. Thus, it exhibits dual functionality as a biocontrol agent (Stoica et al., 2019). *B. licheniformis* strains, such as strain XF32, have exhibited enhanced production of fengycin through genetic modifications, highlighting their potential for agricultural and industrial applications (Zhaojian et al., 2021). Furthermore, strain JF-22 was found to reduce *M. incognita* populations and enrich beneficial microbial communities in tomato rhizospheres, promoting soil health and plant resilience (Du et al., 2022). Unlike Bt, which relies on Cry proteins, or *B. pumilus*, which relies on nutrient deprivation, *B. licheniformis* integrates enzymatic lysis with microbiome enhancement to suppress nematodes. It also supports plant defenses indirectly. Studies have indicated its ability to bolster the resistance of *C. elegans* to bacterial infections through hormonal signaling pathways, such as those involving serotonin, suggesting its potential for inducing systemic resistance in plants (Yun et al., 2014). Advances in genetic engineering, such as promoter and ribosome binding site engineering, have increased the capacity of *B. licheniformis* to produce antimicrobial compounds and enzymes, enhancing its biocontrol potential (Xiao et al., 2024). Field trials have highlighted its dual role in managing nematodes and promoting plant growth. For instance, strain MH48 was found to reduce fungal infections and improve nutrient availability in pine seedlings (Won et al., 2018). The synergy of *B. licheniformis* with other biocontrol agents further enhances its effectiveness in IPM strategies.

### B. firmus

*B. firmus* exhibits remarkable versatility in suppressing nematode populations and enhancing plant growth. As an alkaliphilic, endospore-forming bacterium, it thrives in various soil environments, making it suitable for diverse agricultural systems (Settu et al., 2024). It is distinguished from other *Bacillus* spp. by its ability to colonize plant roots and induce systemic resistance, exhibiting both direct nematicidal effects and indirect plant-protective effects (Huang et al., 2021). A primary mode of action of *B. firmus* involves the production of lytic enzymes, such as chitinases and proteases. These enzymes target the structural integrity of nematode eggshells and cuticles, resulting in the degradation and reduced viability of eggs and juveniles. Genomic studies on *B. firmus* strains, such as strain TNAU1, have identified genes like *chiA* and *chiB*, which are involved in the synthesis of chitinase, an enzyme crucial for breaking down the chitinous components of nematode structures (Settu et al., 2024). This enzymatic degradation not only disrupts nematode development but also facilitates nutrient recycling in the rhizosphere, indirectly benefiting plant health. Moreover, *B. firmus* produces antimicrobial peptides, including surfactin and fengycin, which disrupt nematode cell membranes. These lipopeptides interact with membrane lipids, forming pores that cause ion imbalance, cytoplasmic leakage, and eventual nematode death (Daulagala, 2021). For example, strain YBf-10 can significantly reduce *M. incognita* populations by producing these bioactive compounds, effectively suppressing nematode-induced damage, such as gall formation and egg mass production (Xiong et al., 2015). Among *Bacillus* spp., *B. firmus* is distinguished by its efficacy in reducing nematode reproductive potential. Strain I-1582, widely studied for its nematicidal efficacy, can suppress egg hatching and juvenile viability by producing proteases and secondary metabolites. These metabolites interfere with nematode signaling pathways essential for reproduction and development, offering a comprehensive mechanism for population control (Huang et al., 2021). Furthermore, *B. firmus* promotes plant growth by enhancing nutrient uptake and root colonization, thereby effectively mitigating the damage caused by nematode infestations. Comparative analyses have revealed that *B. firmus* differentiates itself from other *Bacillus* spp. through its robust adaptability to diverse soil pH levels and its ability to induce systemic resistance. Unlike Bt, which relies on Cry proteins for specific gut receptor targeting, or *B. subtilis*, which is known for its VOC-mediated effects, *B. firmus* integrates multiple mechanisms, including enzymatic degradation, lipopeptide production, and systemic resistance induction, to combat nematodes and support plant health. The dual role of *B. firmus* in nematode suppression and plant growth promotion highlights its suitability for sustainable agricultural practices. Recent advancements in genomic studies have further elucidated the biocontrol potential of *B. firmus*. For instance, strain TNAU1 harbors genes encoding nematode-virulent proteases and other antimicrobial compounds, which can enhance its specificity and efficacy against PPNs. Additionally, *B. firmus* YBf-10 can modulate microbial communities in the rhizosphere, enriching beneficial microbes and suppressing harmful pathogens. Thus, it can play a role in IPM strategies (Marin-Bruzos et al., 2021). Field applications of *B. firmus* include soil drenching and seed treatments, which ensure effective delivery of bioactive compounds to nematode hotspots. Pot experiments using soil-drenched YBf-10 revealed substantial reductions in nematode populations and an increase in overall plant growth, showcasing its practical applicability in real-world agricultural systems (Xiong et al., 2015). *B. firmus* employs a multifaceted approach involving enzymatic lysis, antimicrobial activity, and systemic resistance induction for managing nematodes. Its ability to thrive in diverse soil environments, its biocontrol efficacy, and its plant growth-promoting properties underscore its potential as a key agent in sustainable nematode management and IPM strategies.

### B. nematocida

*B. nematocida* is a spore-forming bacterium with distinct nematicidal properties. Thus, it is a pivotal agent for managing PPNs. This bacterium is predominantly found in soil and plant rhizospheres. It utilizes a multifaceted approach involving enzymatic, biochemical, and molecular strategies, which collectively contribute to its efficacy (Huang et al., 2005). Its nematicidal action is attributed to its ability to secrete lytic enzymes, such as chitinases and proteases, which are encoded by genes like *chiA*, *chiB*, *aprE*, and *nprB*. These enzymes target and damage the structural integrity of nematode eggshells and cuticles, directly impairing nematode survival and reproduction. The breakdown of these protective structures not only suppresses nematode populations but also releases essential nutrients, thereby enhancing soil fertility (Sun et al., 2024). Moreover, *B. nematocida* produces antimicrobial lipopeptides, such as fengycin, surfactin, and bacillomycin. These bioactive metabolites disrupt nematode cell membranes by interfering with lipid bilayers, resulting in pore formation, ion leakage, and eventual mortality (Niu et al., 2006; Niu et al., 2011; Niu et al., 2016; Bo et al., 2022). This biochemical disruption demonstrates the potent antagonistic effects of the bacterium on nematode physiology. A unique aspect of the mode of action of *B. nematocida* is the synthesis of 2-heptanone, a volatile compound that acts as a nematode attractant. These chemical lures nematodes toward the bacterium, enhancing its ability to target and infect nematodes with high precision. This mechanism exemplifies an evolutionary adaptation for host–pathogen interactions, as highlighted by Zhu et al. (2019). Such attractant-based pathogenicity differentiates *B. nematocida* from other *Bacillus* spp., adding a layer of specificity to its biocontrol efficacy. Recent studies have identified adaptive molecular responses in *B. nematocida* under stress conditions. For example, Sun et al. (2018) reported that protein acetylation modulates the enzymatic activity of the bacterium, enhancing its nematicidal efficacy. This adaptive regulation reflects a dynamic interaction between *B. nematocida* and its nematode targets, showcasing the ability of the bacterium to respond to environmental stimuli. Comparative analyses have revealed that *B. nematocida* utilizes a highly specialized approach compared with other *Bacillus* spp. Unlike *B. subtilis*, which primarily induces systemic resistance in plants and produces VOCs, or Bt, which relies on Cry proteins for gut-specific toxicity, *B. nematocida* integrates enzymatic degradation, membrane disruption, and chemical attraction to exhibit nematicidal effects. This multipronged strategy underscores its effectiveness in managing PPNs while minimizing collateral effects on nontarget organisms. The practical application of *B. nematocida* has shown promising results in field trials, with its soil drench formulations and seed treatments effectively reducing nematode populations and enhancing plant growth. The specificity of *B. nematocida* for nematodes reduces the ecological risks often associated with broad-spectrum chemical nematicides. Furthermore, its potential for integration into IPM strategies highlights its role in promoting sustainable agriculture. *B. nematocida* is an advanced biocontrol agent characterized by enzymatic degradation, biochemical toxicity, and adaptive molecular interactions. Its unique mechanisms of action and its specificity for nematodes make it a promising alternative to chemical nematicides, contributing to environmentally sustainable agricultural practices.

### B. amyloliquefaciens

*B. amyloliquefaciens* exhibits robust nematicidal activity. It is distinct from other *Bacillus* spp. because of the production of diverse enzymes and bioactive secondary metabolites. Its efficacy is mainly attributed to its ability to synthesize lipopeptides, such as fengycin and iturin, which disrupt nematode cell membranes. These lipopeptides interact with lipid bilayers and cause pore formation and subsequent cell lysis, resulting in nematode mortality (Ngalimat et al., 2021). Moreover, *B. amyloliquefaciens* secretes hydrolytic enzymes, such as chitinases and proteases, which enzymatically degrade nematode cuticles and eggshells, thereby inhibiting juvenile development and reducing nematode reproduction rates (Migunova and Sasanelli, 2021). Genomic studies have highlighted the roles of various genes, such as *fenA* and *ituD*, in the biosynthesis of these lipopeptides, underscoring the genetic adaptability of the bacterium for biocontrol applications (Luo et al., 2022). In addition to exhibiting direct nematicidal effects, *B. amyloliquefaciens* significantly contributes to soil health and plant growth. It stimulates plant development by producing phytohormones and promotes nutrient availability by altering the soil microbiome. For instance, VOCs produced by *B. amyloliquefaciens* not only suppress pathogens but also enhance root growth and nutrient uptake, reinforcing its dual role as a biocontrol agent and a growth promoter (Chowdhury et al., 2015). Strain FZB42 exhibits these attributes by inducing systemic resistance in plants. ISR is achieved through the activation of JA and ethylene (ET) signaling pathways, resulting in the increased production of defense-related enzymes and antimicrobial compounds that protect plants from nematodes and other pathogens (Chowdhury et al., 2015). The genetic manipulation of *B. amyloliquefaciens* has further enhanced its efficacy. For example, the fusion of *B. amyloliquefaciens* SA5 with *Lysinibacillus sphaericus* created a hybrid strain (Bas8) with elevated chitinase production. This strain exhibited significant nematicidal effects against *M. incognita* in controlled trials (Abdel-Salam et al., 2018). Similarly, Liu et al. (2013) demonstrated that the deletion of the gene *RBAM_007470*, responsible for the synthesis of plantazolicin, reduced the nematicidal efficacy of strain FZB42, highlighting the importance of specific metabolites in biocontrol strategies. Field and greenhouse trials have substantiated the biocontrol potential of *B. amyloliquefaciens*. For example, applications of this bacterium at varying concentrations (50–200%) effectively suppressed *M. javanica* in common beans by inhibiting juvenile hatching and reducing motility. These effects were observed both *in vitro* and *in vivo*, showcasing its adaptability across different environmental conditions (Messa et al., 2019). Furthermore, the spiral nematode *Helicotylenchus dihystera* was effectively controlled in soybean fields treated with *B. amyloliquefaciens*-based formulations, with the nematicidal effects being comparable to those of chemical nematicides, such as abamectin. Improvements were also noted in soybean yield and soil health (Camatti et al., 2023). Compared with other *Bacillus* spp., *B. amyloliquefaciens* uniquely combines potent direct nematicidal mechanisms with plant growth-promoting traits. While Bt primarily relies on Cry proteins for nematode control and *B. subtilis* relies on systemic resistance induction, *B. amyloliquefaciens* integrates membrane disruption, enzymatic degradation, and systemic resistance induction, making it a versatile and holistic agent for nematode management. Its ability to modulate the soil microbiome and enhance nutrient cycling further distinguishes it as an indispensable component of sustainable agricultural practices. Overall, *B. amyloliquefaciens* employs a synergistic blend of biochemical, enzymatic, and ecological strategies to control PPNs and enhance plant health. Continued research on its genetic pathways, interaction mechanisms, and field applications can further enhance its role in IPM and sustainable agriculture (Table 1).

**Table 1:** Mode of actions of different isolates or proteins from *Bacillus* species against major pytopathogenic nematodes.

S. no.	*Bacillus* species	Isolates/protein name	Target species	Actions	References
1	*B. velezensis*	BMH INV	*M. incognita*	Reduction in the number of galls and eggs in tomato roots	Cruz‐Magalhães et al. (2022)
2	*B. subtilis*	AP-3	*Meloidogyne* spp. *Pratylenchus* spp.	Effective nematode control when applied post-emergence in sugarcane crops	Mazzuchelli et al. (2020)
3	*B. paralicheniformi* *B. subtilis*	FMCH001 FMCH002	*Meloidogyne* spp.	Decreased egg hatching and juvenile survival	Díaz-Manzano et al. (2023)
4	*B. thuringiensis* *B. velezensis*	KYC CE 100	*Meloidogyne incognita*	Structural damage to nematode eggs and second-stage juveniles	Choi et al. (2020)
5	*B. cereus*	09B18	*Heterodera filipjevi*	High mortality of second-stage juvenile nematodes and reduced egg hatchability	Zhang et al. (2016)
6	*B. subtilis* *B. amyloliquefaciens*	OKB105 FZB42	*Aphelenchoides besseyi* *Ditylenchus destructor* *Bursaphelenchus xylophilus* *M. javanica*	Significant inhibition of growth and increased mortality percentage	Xia et al. (2011)
7	*B. subtilis*	Bbv 57	*M. incognita*	Reduction in egg hatching capacity and increased juvenile mortality	Ramyabharathi et al. (2020)
8	*Bacillus cereus* *B. subtilis*	137JC 18JC	*M. exigua*	High mortality of second-stage juvenile nematodes	Oliveira et al. (2014)
9	*B. velezensis*	BZR 86	*M. incognita*	Decreased egg hatchability and number of root galls in tomato and cucumber plants	Migunova et al. (2021)
10	*B.thuringiensis* (crystal proteins)	Cry55Aa, Cry6Aa, Cry5Ba	*M. hapla*	Midgut toxicity in second-stage juveniles	Zhang et al. (2012)
11	*B. thuringiensis*	YBT-1518	*M. hapla*	Toxic to second-stage juveniles	Guo et al. (2008)
12	*B.thuringiensis* (crystal proteins)	Cry6A	*M. hapla*	Toxicity to second-stage juveniles, reduced galling index, and egg masses	Yu et al. (2015)
13	*B.thuringiensis* (crystal proteins)	Cry5Ca1 Cry5Da1	*M. incognita*	Negative effects on nematode lifespan, fertility, and survival	Geng et al. (2017)
14	*B. megaterium*	YMF3.25	*M. incognita*	Decreased egg hatchability and reduced nematode infections by producing nematicidal volatile compounds	Huang et al. (2010)
15	*B. cereus*	Bc-cm103	*M. incognita*	Over 90% mortality rate in second-stage juveniles (J2)	Yin et al. (2021a)
16	*B. altitudinis*	AMCC 1040	*M. incognita*	Volatile compounds block nematode growth	Ye et al. (2022)
17	*B. velezensis*	BZR 86 BZR 277	*M. incognita*	High nematicidal activity with improved cucumber plant growth under greenhouse conditions	Asaturova et al. (2022)
18	*B. firmus*	I-1582	*H. schachtii*	Negative impact on nematode reproduction, pathogenicity, and development of the next generation	Huang et al. (2021)
19	*B. amyloliquefaciens* *B. firmus* *B. licheniformis* *B. subtilis*	BV03, PTA4838 MBI600 Bf-I1582 FMC001 FMC002	*Helicotylenchus dihystera*	*Bacillus* filtrates reduce nematode growth and survival	Camatti et al. (2023)
20	*B. firmus*	I-1582	*M. incognita*	Degrades eggshells, colonizes eggs, and improves systematic resistance in tomato plants	Ghahremani et al. (2020)
21	*B. cereus* *B. proteolyticus*	IBCBb130 IBCBb116	*M. incognita*	High mortality rates (>68%) in second-stage juveniles (J2)	Amorim et al. (2024)
22	*B. velezensis*	YS-AT-DS1	*M. incognita*	Reduced infection rate of second-stage juveniles (J2) and the number of galls and egg masses on tomato roots	Hu et al. (2022)
23	*B. velezensis*	Pt-RP9	*Bursaphelenchus xylophilus*	Over 90% mortality rate and lower reproduction rate	Sun et al. (2024)
24	*B. cereus,* *B. megaterium,* *B. subtilis* *B. thuringiensis*	*Bacillus* sp. mixture filtrate	*M. arenaria* *M. incognita* *M. javanica* *M. enterolobii*	85–90% immobility of Meloidogyne spp. (J2) after 96 h	Engelbrecht et al. (2022)
25	*Bacillus* Sp.	Bacterial volatiles	*M. graminicola*	Lethal to second-stage juveniles (J2) and significantly reduced infection of susceptible rice	Bui et al. (2020)
26	*B. velezensis*	Bv-25	*M. incognita*	Achieved 100% J2 mortality, decreased expression of ord-1, mpk-1, and flp-18 genes in *M. incognita*, and elevated expression of defense genes (pr1, pr3, and lox1) in cucumber plants	Tian et al. (2022)
27	*B. halotolerans* *B. kochii* *B. oceanisediminis* *B. pumilus* *B. toyonensis* *B. cereus* *B. pseudomycoides*	DDWA DDWB DDWC DDWD DDWNEI DDWWAI JNC	*M. incognita*	Suppressed *M. incognita* up to 69.96% under greenhouse conditions and increased tomato yield	Liu et al. (2020)
28	*B. altitudinis*	AMCC1040	*M. incognita*	Reduced root-knot nematode damage to ginger	Wang et al. (2021a)
29	*B. cereus*	AMA3 AA3 YW4	*Bursaphelenchus xylophilus*	Reduced survival, fecundity, and host adaptability	Yuan et al. (2023)
30	*B. cereus* *B. megaterium*	KMT-5 KMT-8	*M. javanica*	Declined egg hatchability (96%) and 89% second-stage juvenile (J2) mortality	Antil et al. (2022a)
31	*B. subtilis*	MTCC441	*M. incognita*	Egg mortality of 85% at 35 ppm dosage and maximum ovicidal activity (83%)	Nadeem et al. (2021)
32	*B. megaterium*	GIUBAM-2020	*M. incognita*	Volatile organic compounds induced oxidative stress leading to mortality	Maqsood et al. (2024)
33	*B. cereus*	NJSZ-13	*B. xylophilus*	Protease isolated from *B. cereus* causes cuticle degradation	Li et al. (2023)
34	*B. cereus* *B. mycoides* *B. subtilis* *Bacillus* sp.	RBI2AB2.1 RBI2AB2.2 RBIKDA2.2 IR.1.3.4 RBI1IBPL2.3 RBIKDA1.2 RZ21AP1 RZ22AG2	*Meloidogyne Sp.*	Decreased the number of eggs and second-stage juveniles (J2)	Habazar et al. (2021)
35	*B. aryabhattai*	KMT-4	*M. javanica*	73% reduction in eggs and 80% reduction in galls in plant roots (*S. lycopersicum*)	Antil et al. (2021)
36	*B. subtilis*	Culture filtrate	*M. incognita*	Eggs were infected up to 48%	Singh et al. (2021)
37	*B. subtilis*	AP-3	*M. incognita*	Promoted reduction of nematode reproduction factor and gall index in the roots	Bavaresco et al. (2021)
38	*B. velezensis*	AP03 S2527 S2545	*M. incognita*	Increased second-stage juvenile (J2) mortality and inhibited egg hatchability	Pacifico et al. (2021)
39	*B. paralicheniformis*	TB197	*M. incognita*	Showed >95% of nematicidal activity under *in vitro* and *in vivo* conditions	Chavarria-Quicaño et al. (2023a,b)
40	*B. velezensis*	FC37	*M. hapla*	Reduced plant disease severity, crown incidence and severity, and petiole colonization	Camacho et al. (2023)
41	*B. simplex*	Sneb545	*H. glycines*	Improved disease resistance in soybean roots	Kang et al. (2020)
42	*B. megaterium*	Sneb207	*H. glycines*	Reduced the number of cysts, SCN juveniles, and eggs and promoted soybean growth	Zhou et al. (2021)
43	*B. cereus*	Bc-cm103	*M. incognita*	Volatile compounds delivered fumigation activity and higher mortality rates (97.2%) of j2.	Yin et al. (2021b)
44	*B. flexus* *B. pumilus* *B. cereus* *B. megaterium* *B. subtilis*	DK-Sa-A1 KB-Se-A2 KT-Se-A2 PB-Sa-A2 SRJ-Sa-A1	*G. rostochiensis*	≥50% cyst and egg mortality	Widianto et al. (2021)
45	*B. subtilis*	MCC 0067	*M. javanica*	Decreased the nematode numbers (36%) in combination with *Glomus mosseae* and *Trichoderma harzianum*	Sohrabi et al. (2020)
46	*B. velezensis*	VB7	*M. incognita*	Increased juvenile mortality by 87.9% and Induced MAMP-triggered nematode immunity increasing the expression of defense genes WRKY, LOX, PAL, MYB, and PR	Kamalanathan et al. (2023)
47	*B. altitudinis*	123.en	*M. incognita*	Reduced the number of galls, egg masses, and juvenile populations in the soil, and increased expression of pathogenesis-related genes (PR-1 and PR-5) in treated kiwi fruit plants	Banihashemian et al. (2023)
48	*B. subtilis*	-	*M. incognita*	Bacteria grown with MnCl2 and CuCl2 significantly increased juvenile mortality and suppressed their chemotaxis response to tomato roots	Adiwena et al. (2023)
49	*B. atrophaeus*	GBSC56	*M. incognita*	GBSC56 volatiles caused high juvenile mortality, induced oxidative stress, and enhanced defense gene expression in tomato plants	Ayaz et al. (2021)
50	*B. amyloliquefaciens*	D747	*M. enterolobii*	reduced nematode eggs and gall index on cucumber roots in combination with *Purpureocillium lilacinum*	de Paula et al. (2024)
51	*B. cereus*	NRC12	*M. incognita*	Protoplast fusion between *B. cereus* and *B. thuringiensis* produced a fusant strain that significantly increased nematode mortality and enhanced eggplant growth	Mohamed et al. (2021)
52	*B. amyloliquefaciens*	SA5	*M. javanica*	Fusant strains of *B. amyloliquefaciens and Lysinibacillus sphaericus delivered significant inhibition of nematode*	Osman et al. (2020)
53	*B. altitudinis*	KMS-6	*M. javanica*	reduced nematode infestations and enhanced eggplant growth compared to Carbofuran treatment	Antil et al. (2022b)
54	*B. thuringiensis*	B7	*Meloidogyne ssp.*	high inhibitory activity, killing up to 89.67% nematode eggs and 100% of J2 within 10 h	Khanh (2020)
55	*B. cereus*	BCM2	*M. incognita*	BCM2 extracellular proteins caused 100% mortality by damaging the nematode cuticle and eggshell, leading to content leakage	Hu et al. (2020)
56	*B. megaterium* *B. safensis*	C3 VW3	*M. javanica*	Inhibition of Egg-hatchability (20–28%)	Rostami et al. (2021)
57	*B. subtilis*	SJ19	*Meloidogyne* spp.	controlled 67.75% of tomato root-knot nematodes in combination with other biological compounds	Shu et al. (2021)
58	*B. megaterium* *B. subtilis*	–	*M. incognita*	nematicidal potential and significant chitinolytic activity combination with *Serratia marcescens*	Abdellatif et al. (2021)
59	*Bacillus* Sp.	GBSC56 SYST2 FZB42	*Aphelenchoides besseyi*	Compounds of *Bacillus* sp. demonstrated high nematicidal activity against and enhanced growth and defense gene expression in rice seedlings	Ali et al. (2023)
60	*B. amyloliquefaciens* *B. firmus*	QST713 I-1582	*M. incognita*	Effectively manage nematode in cotton through direct antagonistic capabilities and systemic resistance involving JA and SA pathways	Gattoni et al. (2022)
61	*B. subtilis*	MN252542.1	*M. javanica*	Showed 100% mortality and hatching inhibition of nematodes in combination with *P. fluorescens*	Das et al. (2021)
62	*Brevibacillus laterosporus*	F5 strain	*M. incognita*	over 90% mortality of juveniles	Hamze and Ruiu (2022)
63	*B. wiedmannii*	MW405861	*M. incognita*	significantly reduced nematode galls and egg masses in tomatoes in combination with *S. liquefaciens*	Moslehi et al. (2021)
64	*B. pumilus* *B. megaterium* *B. subtilis* *B. cereus*	MZ675428 MZ675429 MZ675430 MZ675431	*M. incognita*	Reduced nematode root galling and reproduction on tomatoes, enhancing plant defense gene expression and enzyme activity than chemical nematicide fluopyram	Devindrappa et al. (2023)
65	*B. subtilis*	-	*M. incognita*	Lettuce roots treated with *B. subtilis* made root exudates repellent to J2	Cavalcanti et al. (2024)
66	*B. pumilus*	S1-10	*M. incognita*	Volatile compound (2-(methylamino)-ethanol (2-ME)) reduced nematodes growth and eggs	Dai et al. (2023)
67	*B. wiedmannii*	AzBw1	*M. arenaria*	Siderophores, protease, and chitinase from *Bacillus* sp. with protease activity nematicidal effect reducing egg hatching by 34% and increasing juvenile mortality by 33.5%.	Fallahzadeh-Mamaghani et al. (2023)
68	*Bacillus* Sp.	Soil filtrate	*M. incognita*	Reduced the growth and development of J2.	Engelbrecht et al. (2020)
69	*B. thuringiensis*	App6Aa2 Cry13Aa1 Cry12Aa1 Cry5Ba3 Xpp55Aa1 Cyt8Aa1	*B. xylophilus*	Toxic proteins delivered higher toxicity and severe intestinal damage to nematode	Guo et al. (2022)
70	*B. amyloliquefaciens* *B. megaterium* *B. thuringiensis* *B. weihenstephanensis* *B. frigoritolerans*	FR203A FB133M FS213P FB833T FB25M FB37BR	*M. ethiopica* *X. index*	Reduced nematode index damage and reproductive indices in grapevine roots	Aballay et al. (2020)
71	*Bacillus* Sp.	85 isolates	*M. incognita*	Twenty-three *Bacillus* isolates caused >75% mortality of juveniles, and 10 strains inhibited nematode development in pot experiments	Wang et al. (2021b)

### B. velezensis

**Figure 4:**
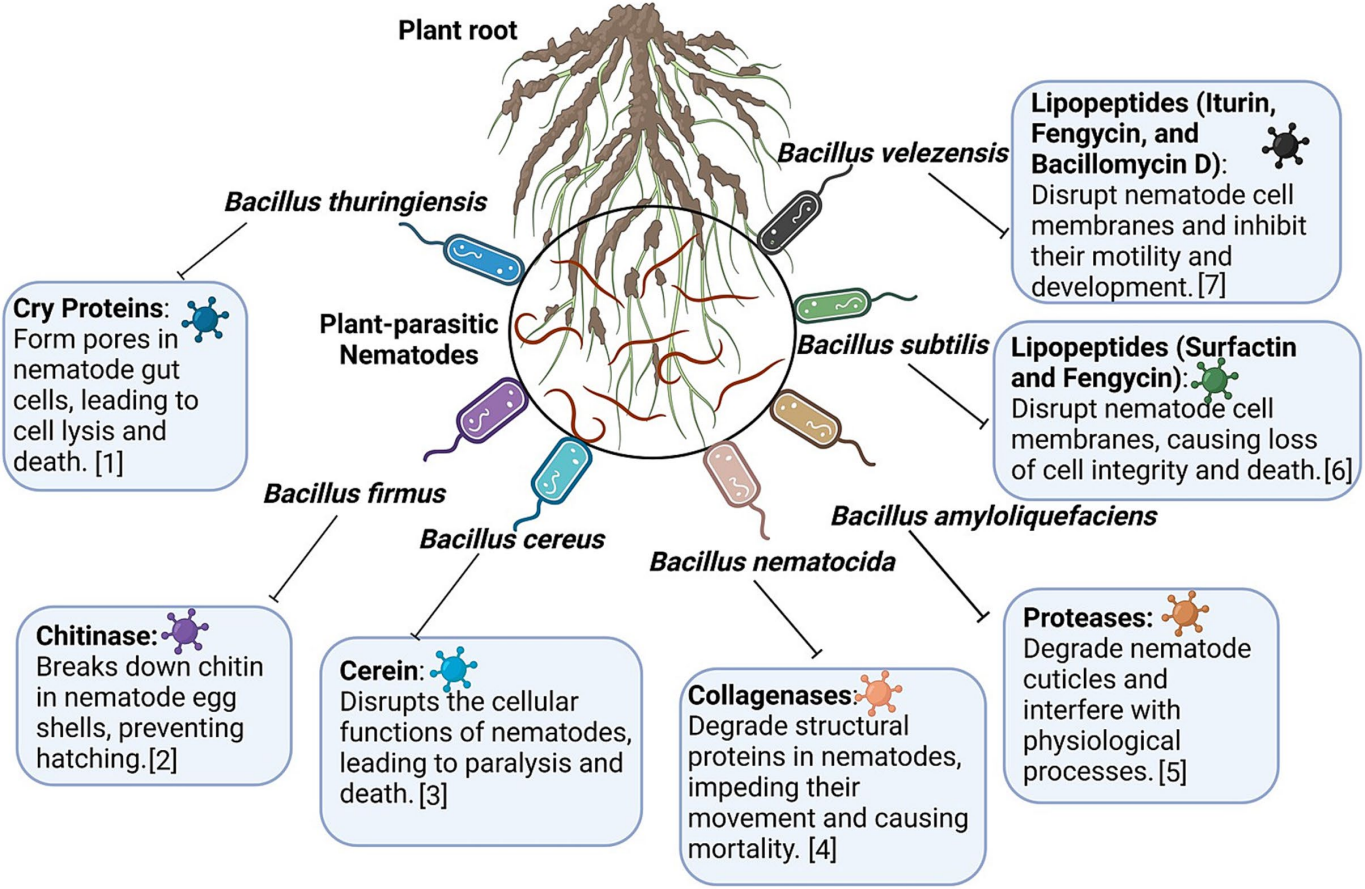
Major *Bacillus* species and their diverse array of proteins and secondary metabolites against the plant-parasitic nematodes. Information adapted from [1] Kahn et al. (2021), [2] Ghahremani et al. (2020), [3] Kulkova et al. (2023), [4] Niu et al. (2006), [5] Jamal et al. (2017), [6] Manju and Subramanian, 2017, and [7] Hu et al. (2022).

*B. velezensis*, a species closely related to *B. amyloliquefaciens*, exhibits substantial nematicidal activity by producing diverse bioactive compounds, making it a key player in sustainable agriculture. Its effects are mainly attributed to the production of lipopeptides (surfactin, fengycin, and iturin), polyketides, and siderophores, which collectively target PPNs and other phytopathogens (Rabbee et al., 2019, 2023). These compounds act by disrupting cell membranes, interfering with metabolic pathways, and creating a hostile environment for pathogens. Moreover, *B. velezensis* contributes to soil health by promoting beneficial microbial communities and enhancing nutrient cycling, making it a multifunctional agent in IPM systems. The nematicidal efficacy of *B. velezensis* has been well documented in controlled environments (Wu et al., 2023). For instance, strain YS-AT-DS1 was found to significantly reduce *M. incognita* infection rates in tomato plants by affecting water and solute transport mediated by TIP genes, without activating the JA or SA pathway (Hu et al., 2022). This finding highlights the unique mode of action of the strain compared with other *Bacillus* spp., which often rely heavily on ISR through JA/SA pathway activation. Another prominent strain, GB03, has been extensively studied for its ability to enhance plant growth and immunity by producing VOCs that prime plant defenses by inducing systemic resistance (Jang et al., 2023). Strain GB03 is recognized for its practical applications. It has also been validated by the U.S. EPA as an eco-friendly alternative to synthetic pesticides. Its ability to suppress nematodes, fungi, and bacteria while concurrently promoting plant health underscores its versatility. Genome sequencing of *B. velezensis* strains, such as strains Ag109 and FZB42, has provided a robust genetic basis for secondary metabolite production. The genome of these strains has been found to contain 13 gene clusters responsible for the synthesis of antimicrobial compounds (Borriss et al., 2019). These metabolites, including surfactin, bacillomycin, and fengycin, not only inhibit nematode activity but also suppress fungal pathogens, providing a comprehensive biocontrol solution. In one study, strain Ag109 was found to reduce *M. javanica* and *P. brachyurus* populations by 69 and 45%, respectively, while exhibiting notable antifungal properties (Mian et al., 2024). Greenhouse studies further validated the nematicidal potential of *B. velezensis*. Strains BMH and INV caused over 90% reductions in *M. incognita* gall formation and egg masses while concurrently enhancing tomato growth (Cruz‐Magalhães et al., 2022). However, combining these strains did not enhance efficacy, suggesting that competitive interactions among strains limit their synergistic potential. A novel approach combining *B. velezensis* with *T. harzianum* and gamma radiation-induced mutants caused significant reductions in *M. javanica* egg hatching (16–45%) and juvenile mortality (30–46%). This synergistic approach, when supplemented with chitosan, led to a 94% reduction in nematode reproduction factors under greenhouse conditions (Rostami et al., 2021, 2024). While *B. velezensis* has gained widespread recognition for its biocontrol properties, its dual nature requires careful management. Reports of pathogenicity in various crops, such as peaches, onions, and potatoes, necessitate stringent application strategies to avoid unintended consequences (Rabbee et al., 2019). Hence, understanding strain-specific interactions and environmental conditions is crucial to optimize its use. Compared with other *Bacillus* spp., *B. velezensis* has unique strengths, including its genetic diversity, robust secondary metabolite production ability, and ability to influence plant physiology through nontraditional ISR pathways. For its integration into sustainable agriculture, further research should be conducted on its ecological interactions and application methodologies to ensure that its potential is maximized and risks are minimized. The major *Bacillus* spp. and their diverse array of proteins and secondary metabolites against PPNs are schematically displayed in Figure 4.

### Application methods and strategies

Various methods and strategies can be used for the application of *Bacillus* spp. to effectively manage phytopathogenic nematodes. A widely used approach is soil amendment, which involves mixing *Bacillus* inoculants with organic matter, such as compost or manure, to improve soil structure and health. This method indirectly suppresses nematode populations by fostering beneficial microbial communities and enhancing plant resilience (Fabiyi, 2024). Seed treatment is another effective strategy. It involves the coating of seeds with *Bacillus* spores before planting. This approach confers early protection to seedlings by colonizing the root zone and creating a hostile environment for nematodes. Additionally, foliar sprays with *Bacillus* formulations can induce systemic resistance in plants, thereby activating defense mechanisms that reduce nematode penetration and reproduction. Biofertilizers incorporating *Bacillus* strains can be directly applied to the soil or used for root drenching, thereby enhancing nutrient availability and promoting robust plant growth. This can help plants withstand nematode attacks.

**Figure 5:**
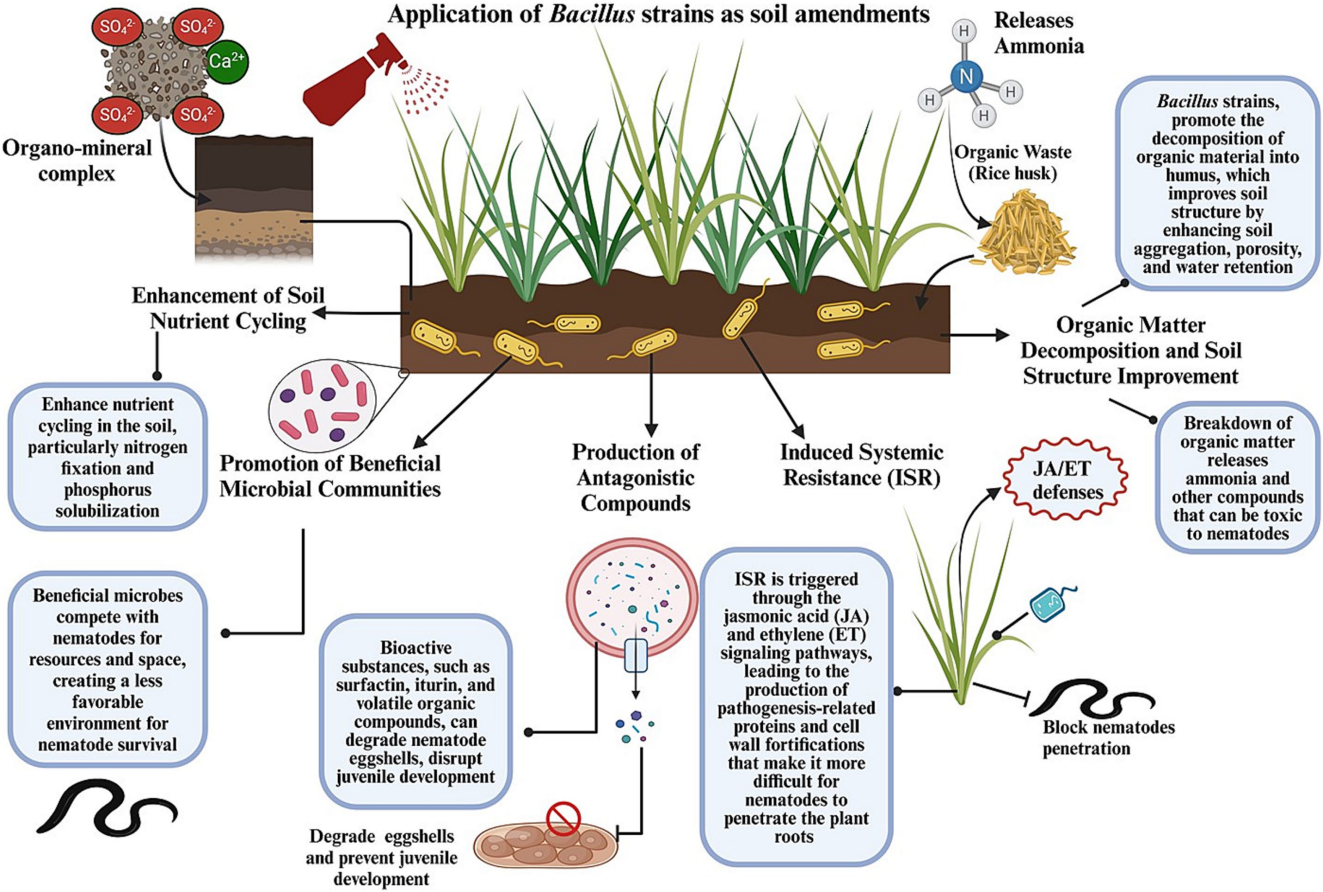
Graphical representation of how the application of *Bacillus* strains as soil amendments alone or in combination with organic matter enhances soil health and structure and reduces nematode proliferation through several interrelated mechanisms.

In IPM programs, *Bacillus* strains are often combined with other biocontrol agents, chemical treatments, or cultural practices, providing a multifaceted approach for the management of nematodes. For instance, integrating *B. subtilis* with organic amendments and reducing the use of chemical nematicides have led to enhanced efficacy against root-knot nematodes, thereby lowering infestations and improving crop yields. Such synergistic approaches can reduce reliance on chemical inputs while maintaining nematode suppression. *B. amyloliquefaciens* formulations have exhibited notable efficacy in field trials by reducing cyst nematode populations and promoting plant health through the induction of systemic resistance. This approach reduces reliance on chemical nematicides and promotes sustainable agricultural practices. *Bacillus* strains are being increasingly recognized for their potential for managing PPNs because of their diverse modes of action and adaptability to different agricultural environments. They produce various secondary metabolites, such as lipopeptides, enzymes, and antibiotics, which directly inhibit nematodes through a process known as direct antagonism (Iftikhar et al., 2020). These metabolites disrupt nematode membranes, degrade their structural proteins, or interfere with their signaling pathways, resulting in reduced nematode viability and infectivity (Bhat et al., 2023). The detailed mechanisms of the different application strategies of *Bacillus* spp. for managing nematodes are outlined below and presented in Figure 5.

### ISR

*Bacillus* strains can trigger plant defense mechanisms, enhancing the ability of plants to resist nematode infections (Choudhary and Johri, 2009). ISR is achieved through the upregulation of plant defense-related genes, resulting in the production of pathogenesis-related proteins and other defense-related compounds that inhibit nematode invasion and reproduction (Mahapatra et al., 2022). *Bacillus* strains produce specific elicitors, such as lipopeptides, VOCs, and secondary metabolites, which prime the plants to enhance defense responses. Upon nematode attacks, these primed plants exhibit accelerated production of pathogenesis-related proteins, oxidative enzymes, and secondary metabolites, thereby reducing nematode penetration, nematode reproduction, and overall damage. Adam et al. (2014) found that certain *B. subtilis* strains, known for their antifungal properties, can effectively reduce root-knot nematode infestations in tomatoes, primarily through ISR rather than direct antagonism. This demonstrates the potential of multipurpose bacteria for IPM in nematode–fungal disease complexes. Additionally, Xing et al. (2020) identified six ISR-active compounds from *B. simplex* Sneb545 that conferred resistance against the pathogen *H. glycines* in soybeans. Among these compounds, the cyclic dipeptide Val-Pro, tryptophan, and uracil were particularly effective in inducing defense-related gene expression in soybeans, offering potential novel agents for managing this destructive nematode.

### Soil amendments and biofertilizers

The application of *Bacillus* strains as soil amendments alone or in combination with organic matter can significantly improve soil health and structure, creating an environment less conducive to nematode proliferation. *Bacillus*-based biofertilizers not only enhance plant growth but also foster beneficial microbial communities in the rhizosphere, in turn antagonizing nematodes (Fabiyi, 2024). For instance, Tong-Jian et al. (2013) demonstrated that the use of *B. cereus* strain X5 in combination with bio-organic fertilizers and biofumigation materials significantly improved plant biomass and reduced nematode infestation under greenhouse and field conditions. This suggests its potential for integrated nematode management in agricultural systems. Moreover, a consortium of three plant growth-promoting rhizobacteria—*B. cereus* (AR156), *B. subtilis* (SM21), and *Serratia* sp. (XY21)—was found to reduce root-knot nematode disease severity in cucumbers by up to 72%. This consortium not only enhanced yield and fruit quality but also improved soil properties by increasing the abundance of disease-suppressive bacterial genera in the rhizosphere. The resulting changes in the microbial community positively correlated with improvements in soil chemical properties, contributing to nematode suppression and overall plant health (Zhang et al., 2024). The several interrelated mechanisms through which *Bacillus* spp. improve soil health and reduce nematode proliferation when used as soil amendments alone or in combination with organic matter are illustrated in Figure 5.

### Seed treatment

Treating seeds with *Bacillus* spores confers early protection to seedlings against nematodes. As the seeds germinate, *Bacillus* spp. colonize the root system, forming a protective barrier that hinders nematode penetration and colonization (Diyapoglu et al., 2022). Seeds are treated with *Bacillus* strains using different methods, such as dry coating, wet coating, or pelletization, to ensure even distribution and firm adherence of the bacteria to the seeds. After coating, the seeds are carefully dried and packaged to preserve bacterial viability. Upon planting, *Bacillus* spores germinate alongside the seeds. They colonize the root zone and confer protection against nematodes while promoting plant growth and soil health (Migunova and Sasanelli, 2021).

**Figure 6:**
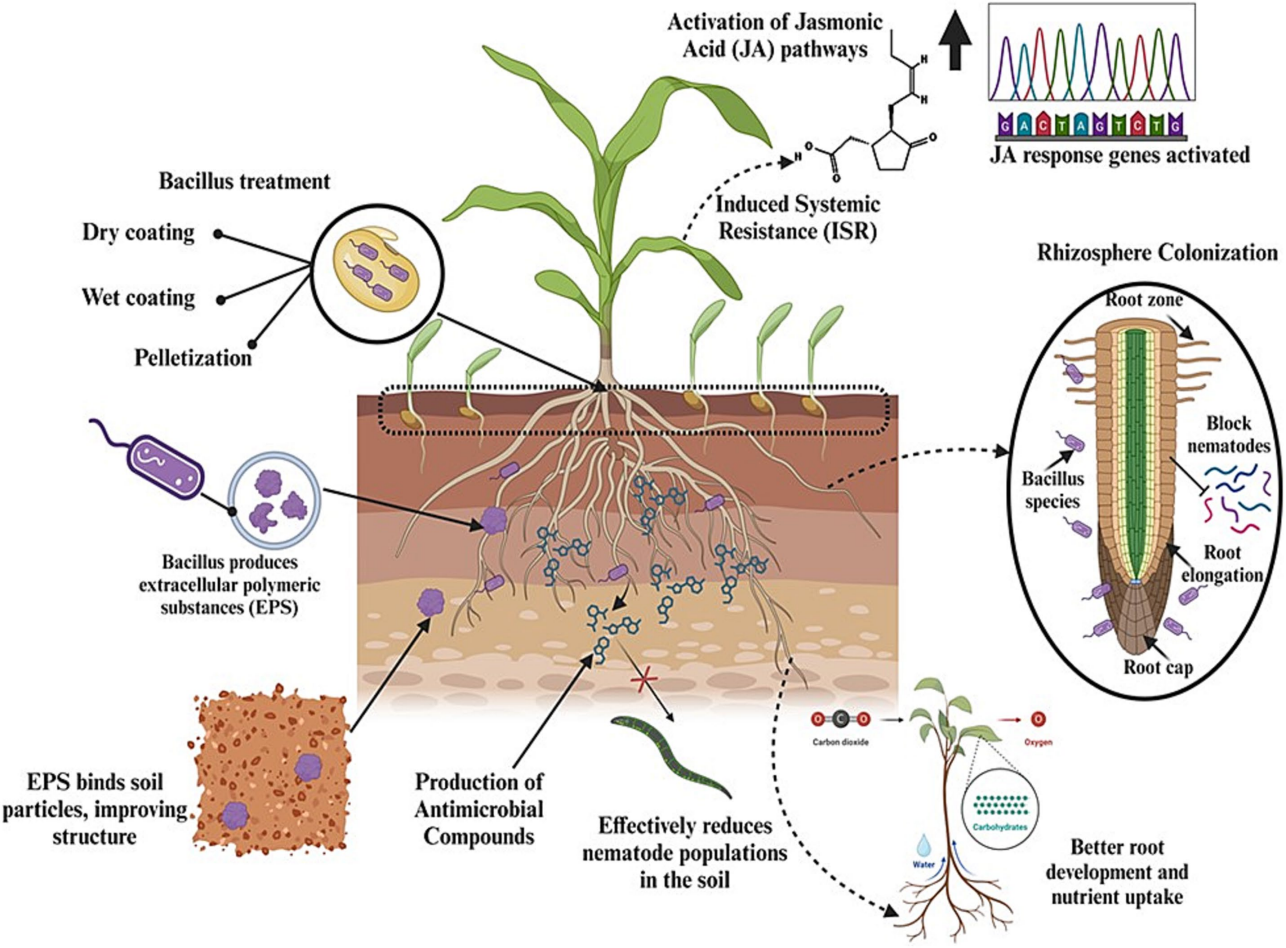
Graphical representation of how seed treatment with *Bacillus* spp. enhances plant growth, improves soil health, and reduces nematode populations.

Seed treatment with *Bacillus* strains can improve soil health and reduce nematode proliferation through several key mechanisms, including the colonization of the rhizosphere, induction of systemic resistance, enhancement of soil microbial communities, production of antimicrobial compounds, improvement of soil structure, and reduction of phytopathogens (Figure 6). When seeds are treated with *Bacillus* strains, these beneficial bacteria colonize the root zone as the plant germinates and grows. This early colonization creates a protective microbial shield around the roots, i.e., the rhizosphere, which acts as the first line of defense against nematode invasion. *Bacillus* strains occupy key ecological niches in the soil and outcompete nematodes for space and nutrients, thereby reducing the likelihood of nematode attachment and penetration into plant roots (Hu et al., 2017). Moreover, *Bacillus* strains induce systemic resistance in plants through seed treatment, priming the immune system of plants to respond more robustly to nematode attacks by activating JA and ET pathways (Choudhary and Johri, 2009). The introduction of *Bacillus* strains via seed treatment enriches the soil microbiome. These beneficial bacteria promote the growth of other advantageous microorganisms, such as mycorrhizal fungi and nitrogen-fixing bacteria, collectively improving soil health and structure. A rich and diverse microbial community enhances nutrient cycling, organic matter decomposition, and soil aggregation, creating a more stable and fertile soil environment that can support healthy plant growth and reduce nematode populations (Chernov and Semenov, 2021). Additionally, antimicrobial compounds produced by *Bacillus* strains can degrade nematode eggs, inhibit juvenile development, and reduce nematode motility, thereby limiting the ability of nematodes to infect plant roots. The persistence of these antimicrobial substances in the rhizosphere helps maintain a soil environment hostile to nematodes (Diyapoglu et al., 2022). Moreover, when applied to seeds, *Bacillus* strains colonize the rhizosphere—the area of soil directly affected by root exudates and associated soil microorganisms—and produce extracellular polymeric substances (EPS). These complex organic molecules are crucial for improving soil structure. EPS act as a natural adhesive and bind soil particles together to form stable aggregates, in turn enhancing soil porosity, promoting better air circulation, and improving water infiltration (O’Callaghan, 2016). Improved soil structure not only enhances root growth and plant vigor but also creates a less favorable environment for nematode movement and survival, as nematodes prefer compact, poorly aerated soils (Khan et al., 2022).

Overall, seed treatment with *Bacillus* strains represents a multifaceted approach for the management of nematodes by enhancing soil health, improving plant resilience, and directly suppressing nematode populations. Thus, it is a more sustainable and effective method of nematode control (Zhang et al., 2009; O’Callaghan, 2016; Hsiao et al., 2023; Hayat et al., 2023).

### Foliar application

Foliar application of *Bacillus* spp. is an effective biocontrol strategy for managing phytopathogenic nematodes (Shafi et al., 2017). This process involves culturing selected *Bacillus* strains and formulating them into a sprayable solution. Optimal timing is crucial for the success of this method, with applications typically performed during early plant growth stages under favorable environmental conditions to ensure effective colonization. Uniform application using sprayers ensures that the bacteria adhere well to plant surfaces, thereby inducing systemic resistance and protecting against nematode damage (Fu et al., 2020). This method has gained popularity in the U.S., China, India, Brazil, Spain, and South Africa, particularly for high-value crops in areas with substantial nematode pressure (Chien and Huang, 2020; Efthimiadou et al., 2020; Karačić et al., 2024). The effectiveness of foliar application is attributed to a combination of direct antagonism, ISR, and plant health enhancement, which collectively reduce nematode populations and improve crop growth and yield (Esitken et al., 2002; Ryu et al., 2011; El-Sawy et al., 2023).

However, the success of this approach hinges on optimizing the application techniques and timing and understanding the specific interactions between *Bacillus* spp., the host plant, and the target nematode species (Shafi et al., 2017). Despite the advantages, including reduced environmental impacts and improved plant vigor, various challenges need to be addressed; these include ensuring consistent root protection and managing environmental variables (Abd-Elgawad and Askary, 2020). Thus, continued research and field trials will be crucial for refining this strategy and integrating it into sustainable nematode management programs.

### Soil health status after the application of *Bacillus* strains

The application of *Bacillus* spp. as biocontrol agents provides multifaceted benefits beyond nematode suppression. *Bacillus* spp. significantly affect overall soil health through biochemical, microbial, and ecological interactions (Vasques et al., 2024). They enhance soil microbial diversity and activity by producing various secondary metabolites, such as lipopeptides, antibiotics, and VOCs, which act as antagonists to soilborne pathogens. These bioactive compounds disrupt the growth of phytopathogenic fungi, bacteria, and nematodes, thereby fostering a healthier and more balanced soil microbiome (Miljaković et al., 2020). Moreover, the metabolites released by *Bacillus* spp. often serve as signaling molecules, promoting beneficial microbial symbiosis and microbial niche differentiation within the rhizosphere. A crucial mechanism through which *Bacillus* spp. influence soil health is the decomposition of organic matter by secreting hydrolytic enzymes, such as cellulases, proteases, and chitinases. These enzymes accelerate the breakdown of complex organic materials into simpler compounds, improving soil organic carbon content and nutrient availability (Riseh et al., 2024). *Bacillus* spp. produce chitinases that degrade chitin-containing structures, such as nematode eggshells and fungal cell walls, thereby facilitating the recycling of essential elements, such as nitrogen and carbon, within soil ecosystems. This degradation process releases N-acetylglucosamine monomers, which serve as nutrient sources for various soil microorganisms, thereby enhancing nutrient cycling and soil fertility. The breakdown of these structures by *Bacillus*-derived chitinases also suppresses soilborne pathogens and pests, contributing to a healthier soil microbiome (Gomaa, 2021). Moreover, *Bacillus* spp. play a vital role in nutrient cycling, particularly in nitrogen fixation and phosphate solubilization. Certain strains, such as *B. subtilis* and *B. megaterium*, possess the genetic and enzymatic machinery required for solubilizing insoluble phosphates in the soil. They produce organic acids (e.g., gluconic acid and citric acid) and phosphatases and convert insoluble phosphates into plant-accessible forms, such as dihydrogen phosphate (Saeid et al., 2018). Several *Bacillus* spp., including *Paenibacillus polymyxa* and *P. macerans*, contain nitrogenase enzymes that enable them to fix atmospheric nitrogen into ammonia, thereby enhancing soil fertility and providing essential nutrients for plant growth. This biological nitrogen fixation facilitates sustainable agricultural practices by reducing the need for chemical nitrogen fertilizers. Studies have demonstrated the efficacy of these bacteria in promoting plant growth through nitrogen fixation (Li et al., 2022). *Bacillus* spp. can enhance soil structure by secreting EPS, which facilitate the aggregation of soil particles. This aggregation improves soil porosity, aeration, and water infiltration, thereby promoting plant root growth and nutrient uptake. Additionally, the production of EPS facilitates moisture retention and reduces soil erosion, thereby enhancing soil resilience under stress conditions. These benefits underscore the role of *Bacillus* spp. in sustainable soil management and plant health enhancement (Olagoke et al., 2022). Moreover, *Bacillus* spp. can induce systemic resistance in plants, indirectly influencing soil health by reducing pathogen pressure. *Bacillus*-treated plants exhibit enhanced production of antimicrobial compounds and defense-related enzymes through the activation of JA and SA pathways. This reduces the likelihood of pathogen colonization and minimizes disease-mediated disruptions to soil microbial dynamics (Kloepper et al., 2004). While *Bacillus* spp. offer numerous benefits as biocontrol agents, their application must be carefully managed to maintain ecological balance within the soil microbiome. Overapplication or improper use can result in the overdominance of *Bacillus* strains, potentially suppressing other beneficial microorganisms and disrupting microbial community structures. This imbalance may result in competition for resources, negatively impacting native microbial populations and overall soil health (Li et al., 2022). Therefore, it is crucial to monitor and regulate the use of *Bacillus*-based biocontrol agents in order to preserve the diversity and functionality of soil microbial communities. Sustainable management practices, including the rotation of microbial inoculants, integrated use of organic amendments, and minimal use of chemical treatments, can mitigate these risks and optimize the long-term benefits of *Bacillus* applications.

### IPM

Incorporating *Bacillus* strains into IPM strategies offers an effective and sustainable approach for the management of phytopathogenic nematodes. *Bacillus* spp., such as *B. subtilis* and Bt, employ multiple mechanisms to suppress nematodes (Gassmann et al., 2008; Jaiswal et al., 2022). These strains not only produce nematicidal compounds but also promote plant growth by producing phytohormones and enhancing nutrient availability. This dual action improves crop health and resilience, further mitigating the impact of nematode infestations (Abd-Elgawad and Askary, 2018).

Within an IPM framework, *Bacillus* strains are most effective when used in combination with other biocontrol agents, chemical nematicides, and cultural practices. For instance, the application of *B. firmus* strain 1–1,582 in combination with chemical nematicides and organic amendments significantly enhanced tomato yield and effectively suppressed *M. incognita* and *P. lycopersici* populations under greenhouse conditions, particularly when environmental conditions were less favorable for nematode development. These findings underscore the potential of *B. firmus* as a viable component of IPM strategies during tomato cultivation (d'Errico et al., 2019).

A recent review by Paradva and Kalla (2023) highlighted the potential of microbial biocontrol agents, particularly *Bacillus*-based nanoparticles, as sustainable and eco-friendly alternatives to chemical pesticides for plant disease and pest management. The synergistic use of *Bacillus* strains with nematophagous fungi or predatory nematodes can confer multilevel protection by targeting different stages of the nematode life cycle (Gassmann et al., 2008; d'Errico et al., 2019).

Native *Bacillus* strains, such as *B. marisflavi* CRB2 and *B. subtilis* CRB7, which harbor multiple antimicrobial peptide genes, have been proven to be effective against *M. incognita* in okra. Within an IPM framework, these strains have caused significant reductions in nematode incidence and improvements in crop yields in laboratory, pot, and field trials (Gurikar et al., 2022). When applied with reduced doses of chemical nematicides, *Bacillus* strains can help lower the use of chemical treatments and maintain effective nematode control, thereby minimizing the potential for resistance development and environmental impacts (Ruiu, 2015). Moreover, cultural practices, such as crop rotation, cover cropping, and the use of organic soil amendments, enhance the efficacy of *Bacillus* applications by creating less favorable conditions for nematode proliferation and supporting a healthier soil microbiome (Singh et al., 2019). For instance, the integration of *B. subtilis* with cow manure resulted in a 54% reduction in PPN populations in common beans and preserved nematode biodiversity, thereby serving as a sustainable and effective pest management strategy (Wepuhkhulu et al., 2011). Furthermore, Rao et al. (2017) demonstrated that the application of *B. subtilis* IIHR BS-2 as a seed treatment in combination with a vermicompost-enriched soil application significantly reduced nematode populations by 69.3% and disease incidence by 70.2%, resulting in a 28.8% increase in carrot yield. This integrated approach outperformed chemical treatments, highlighting the efficacy of *B. subtilis* IIHR BS-2 in managing the *M. incognita–Pectobacterium carotovorum* disease complex in carrots.

**Figure 7:**
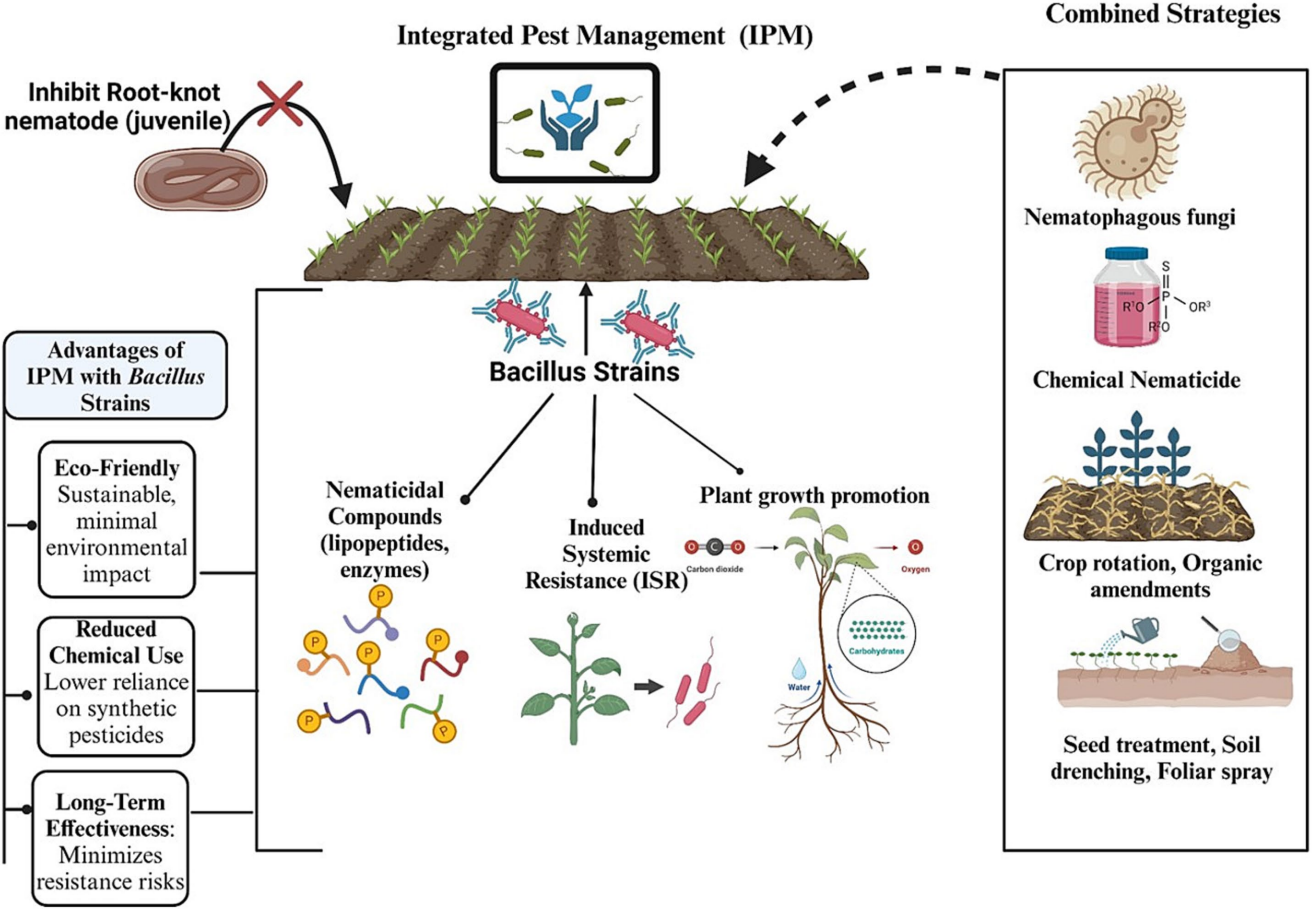
Graphical representation of integrated pest management strategies using *Bacillus* strains for nematode control.

Thus, the strategic incorporation of *Bacillus* strains into IPM programs has several advantages, including sustainable nematode management, enhanced efficacy through synergistic effects, and improved resistance management (Wepuhkhulu et al., 2011). Regular monitoring of nematode populations and crop health is crucial for optimizing the timing and application of *Bacillus* treatments to ensure the highest efficacy in conjunction with other control measures (Chinheya et al., 2017). By integrating *Bacillus* strains into a comprehensive IPM strategy, farmers can achieve long-term nematode suppression, reduce reliance on chemical pesticides, and ultimately improve crop productivity and sustainability in agricultural systems (Figure 7).

### Scalability and cost-effectiveness of *Bacillus* applications

*Bacillus* spp. are recognized for their scalability as biocontrol agents, primarily because of their ability to form resilient spores that can be produced on a large scale through cost-effective industrial fermentation processes (Serrão et al., 2024). These spore-based formulations exhibit extended shelf lives and require minimal storage conditions, thereby reducing logistical expenses for farmers, especially in resource-limited regions (Cho and Chung, 2020). Moreover, *Bacillus* formulations are compatible with existing agricultural practices, including seed treatments, soil amendments, and foliar sprays, facilitating their integration into IPM systems. Their synergistic interactions with organic amendments, such as compost, and microbial consortia further enhance their efficacy and cost-effectiveness (Asif et al., 2024). Economic analyses have indicated that *Bacillus*-based products can significantly reduce reliance on chemical nematicides and fertilizers, resulting in substantial cost savings. For instance, the application of *B. subtilis* during tomato cultivation has been shown to reduce nematode-induced losses by over 60%, resulting in notable yield improvements and financial benefits (Pontes et al., 2024). Case studies from countries like Brazil and India have demonstrated the successful large-scale application of *Bacillus* spp. in soybean and rice production systems, respectively (Galbieri et al., 2023; Pandey et al., 2024). Moreover, smallholder farmers in Africa have adopted these formulations because of their affordability and effectiveness across various crops, including maize and vegetables (Vasques et al., 2024). Cost comparisons have revealed that *Bacillus*-based biopesticides are approximately 30–50% less expensive than chemical alternatives, enhancing their appeal in low-income regions (Hezakiel et al., 2024). In addition to economic advantages, these biopesticides offer significant environmental benefits by reducing pollution and health risks associated with chemical nematicides, thereby contributing to global sustainability goals (Köhl et al., 2019). They also promote ecological balance by enhancing soil biodiversity and mitigating secondary pest outbreaks, further reinforcing their role in sustainable agriculture (Abd-Elgawad, 2024).

### Challenges and limitations

The application of *Bacillus* strains as biocontrol agents for managing nematodes in agricultural systems has several challenges and limitations. Environmental factors, such as soil type and climate, play crucial roles in determining the efficacy of these bacteria. Soil characteristics, including pH, organic matter content, and texture, can significantly influence the survival, colonization, and nematicidal activity of *Bacillus* strains. For instance, sandy soils may cause the bacteria to leach away, while heavy clay soils could limit bacterial distribution (Gurikar et al., 2022). Additionally, climatic conditions, particularly temperature and moisture levels, can significantly influence the efficacy of *Bacillus* spp. (Ayaz et al., 2023). Extreme temperatures can inhibit bacterial activity, while optimal moisture levels are necessary for the germination and functioning of bacterial spores. Furthermore, interactions with other soil microorganisms can limit the establishment of *Bacillus* strains because of competition for resources or antagonistic effects.

In addition to environmental factors, regulatory and safety concerns pose substantial barriers to the widespread use of *Bacillus* strains as biocontrol agents. The approval process for these biocontrol agents involves rigorous testing to ensure their safety for humans, animals, and the environment. This process can be time-consuming and expensive, particularly for smaller companies, thereby delaying the introduction of effective biocontrol products.

The efficacy of *Bacillus* strains in nematode control is significantly influenced by soil type, climatic conditions, and interactions with other soil microorganisms (Shafi et al., 2017; Singh et al., 2023). *Bacillus* strains often perform more consistently in controlled environments, such as greenhouses, where conditions are more predictable and manageable. However, translating the obtained results to field conditions can be challenging because of the variability in environmental factors across different geographical locations and crop systems (Ayaz et al., 2023).

Despite the promising potential of *Bacillus* spp. in managing PPNs, several challenges need to be overcome to ensure consistent efficacy under field conditions. Environmental factors, such as soil type, temperature, moisture level, and pH, can significantly impact the survival, colonization, and biocontrol activity of *Bacillus* strains (Shafi et al., 2017). Additionally, the presence of native soil microbiota can necessitate competitive interactions that may suppress the establishment and function of introduced *Bacillus* spp. Native microorganisms compete with introduced *Bacillus* strains for essential nutrients and ecological niches. This competition can limit the growth and activity of the biocontrol agents, thereby reducing their effectiveness against PPNs. For instance, indigenous soil bacteria may outcompete introduced *Bacillus* strains for carbon sources, thereby inhibiting their proliferation (Mawarda et al., 2022). Moreover, native microorganisms can form biofilms on root surfaces, creating physical barriers that can prevent *Bacillus* spp. from accessing plant roots and exhibiting their biocontrol effects. These biofilms can effectively exclude introduced bacteria from key interaction sites. For instance, biofilms formed by indigenous *Pseudomonas* spp. can inhibit the root colonization of introduced *Bacillus* strains (Steinberg et al., 2020). Additionally, field variability significantly influences the efficacy of *Bacillus* spp. as biocontrol agents, with the outcomes in controlled environments often differing from those in diverse agricultural settings. Environmental factors, such as soil type, pH, moisture level, temperature, and organic matter content, play crucial roles in the survival, colonization, and activity of introduced *Bacillus* strains (Serrão et al., 2024). To overcome these challenges, comprehensive field studies need to be conducted. Moreover, robust *Bacillus* formulations that can withstand environmental fluctuations and can be effectively integrated into existing soil microbial communities need to be developed.

For the commercialization and large-scale application of *Bacillus* strains, significant hurdles related to formulation, storage, and regulatory approval need to be overcome (Montesinos, 2003; Butu et al., 2022). The stability and shelf life of *Bacillus* products can be affected by formulation methods, storage environments, and shipment conditions. To ensure the success of *Bacillu*s spp. as biocontrol agents, it is essential to enhance formulation technologies, extend product shelf life, and reduce production costs (Ortiz and Sansinenea, 2023). Ongoing efforts by researchers and industry partners are focused on optimizing microbial strains for large-scale applications, in addition to ensuring that these products meet rigorous environmental and human health safety standards (Hossain et al., 2023).

These challenges underscore the need for continued research and collaboration to effectively integrate *Bacillus* strains into sustainable agricultural practices. Safety evaluations must also ensure that *Bacillus* strains do not pose risks to nontarget organisms or the environment and do not have unintended ecological impacts, such as the disruption of soil microbial communities or induction of resistance in pest populations (Ayaz et al., 2023). Public perception and acceptance of microbial biocontrol agents further complicate their application, highlighting the need for better education and communication about their safety and benefits. Addressing these challenges is essential to fully harness the potential of *Bacillus* strains for sustainable nematode management (Hossain et al., 2023).

### Recent advances and innovations

Targeted genome editing, particularly CRISPR/Cas9 technology, has revolutionized plant pathology by enabling precise genetic modifications to enhance disease resistance in crops. This technology is preferred for its simplicity, cost-effectiveness, and adaptability, offering a promising approach for the development of pest- and disease-resistant plants (Das et al., 2023; Yin et al., 2024). These genetic modifications often aim to increase the production of antimicrobial compounds, such as lipopeptides, enzymes, and VOCs, which are crucial for suppressing various plant pathogens (Rocha and Duggal, 2023; Maqsood et al., 2024). Additionally, genetic engineering has facilitated the introduction of novel traits, such as enhanced root colonization and rhizosphere persistence, ensuring that engineered *Bacillus* strains are more effective and resilient under diverse environmental conditions (Ramírez-Pool et al., 2024). A recent review by Khan et al. (2023) highlighted that advanced molecular strategies, including transcriptomics, RNA interference, and CRISPR/Cas9, are increasing our understanding of plant–nematode interactions and boosting plant resistance to root-knot nematodes. Engineered *Bacillus* strains exhibit improved activity against nematodes, offering a broad-spectrum biocontrol solution that is highly specific to target pests (Danilova et al., 2023).

Although genetic engineering has significant potential for enhancing the nematicidal efficacy of *Bacillus* strains, its use is associated with several biosafety concerns. Unintended ecological impacts, such as the disruption of native microbial communities or off-target effects on nontarget organisms, must be carefully evaluated (Samal et al., 2024). Horizontal gene transfer poses additional risks, potentially resulting in the spread of engineered traits to unintended microbial populations. Regulatory hurdles, including stringent testing for environmental and public health safety, also pose significant challenges. For example, the process of obtaining approval for genetically modified *Bacillus* strains varies across jurisdictions, with extensive environmental impact assessments required to ensure compliance with biosafety standards (Rozas et al., 2024).

Formulation improvements have been a major focus in the advancement of *Bacillus*-based biocontrol products (Tong-Jian et al., 2013; Umamaheswari et al., 2020). Innovations in this area include the development of more stable and effective formulations to maximize the viability and efficacy of *Bacillus* derivatives (Chavarria-Quicaño et al., 2023a,b). A significant advancement is microencapsulation. In this process, spores are enclosed within a protective matrix to shield them from environmental stressors while enabling controlled release (Gao et al., 2024). This technique has been crucial for maintaining the viability of spores over extended periods, thereby enhancing the shelf life and effectiveness of the product (Khullar et al., 2024).

Researchers are also exploring synergistic combinations of *Bacillus* strains with other biocontrol agents or biostimulants in order to create multifunctional formulations that can confer comprehensive plant protection and promote plant growth. Advances in delivery systems and increases in shelf life have further revolutionized the application of *Bacillus*-based biocontrol agents (Karačić et al., 2024). Novel delivery systems, such as nano-bioformulations and polymer-based carriers, are being developed to optimize the precision and efficacy of *Bacillus* applications (Behl et al., 2024). These systems are designed to optimize the release of active agents at the site of infection, thereby reducing the need for frequent applications and lowering the overall costs (Kumar et al., 2021). Moreover, improvements in storage technology, including the development of temperature-stable formulations and vacuum packaging techniques, have significantly extended the shelf life of *Bacillus* products (Gotor-Vila et al., 2019). These innovations not only ensure the long-term viability of biocontrol agents but also enhance their accessibility on a global scale, particularly in regions with challenging storage and transportation conditions.

## Conclusion

*Bacillus* spp. have emerged as potent biocontrol agents against PPNs, offering a promising and sustainable alternative to traditional chemical treatments. Their effectiveness is attributed to their multifaceted mechanisms, including the production of nematicidal compounds, enhancement of plant resistance, and improvement of soil health. Thus, they play invaluable roles in IPM strategies. Recent advances in genetic engineering and formulation technologies have significantly bolstered the efficacy and reliability of *Bacillus* strains for agricultural applications. However, various challenges, such as environmental variability, regulatory hurdles, and the need for optimized application methods, persist. Overcoming these challenges is essential for maximizing the efficacy of *Bacillus* spp. in sustainable nematode management and ensuring global food security.

## References

[ref1] Aballay E. Prodan S. Correa P. Allende J. (2020). Assessment of rhizobacterial consortia to manage plant parasitic nematodes of grapevine. Crop Prot. 131:105103. doi: 10.1016/j.cropro.2020.105103

[ref2] Abd-Elgawad M. M. (2024). Upgrading strategies for managing nematode pests on profitable crops. Plan. Theory 13:1558. doi: 10.3390/plants13111558, PMID: 38891366 PMC11174438

[ref3] Abd-Elgawad M. M. Askary T. H. (2018). Fungal and bacterial nematicides in integrated nematode management strategies. Egypt. J. Biol. Pest Control 28, 1–24. doi: 10.1186/s41938-018-0080-x, PMID: 39743787

[ref4] Abd-Elgawad M. M. M. Askary T. H. (2020). Factors affecting success of biological agents used in controlling the plant-parasitic nematodes. Egypt. J. Biol. Pest Control 30:17. doi: 10.1186/s41938-020-00215-2

[ref5] Abdellatif A. A. Tahany A. R. Sayed M. A. Dina I. Elmaghraby M. M. K. (2021). Activity of *Serratia* spp. and *Bacillus* spp. as biocontrol agents against *Meloidogyne incognita* infecting tomato. Pakist. J. Biotechnol. 18, 37–47. doi: 10.34016/pjbt.2021.18.2/3.37

[ref6] Abdel-Salam M. S. Ameen H. H. Soliman G. M. Elkelany U. S. Asar A. M. (2018). Improving the nematicidal potential of *Bacillus amyloliquefaciens* and *Lysinibacillus sphaericus* against the root-knot nematode *Meloidogyne incognita* using protoplast fusion technique. Egypt. J. Biol. Pest Control 28, 1–6. doi: 10.1186/s41938-018-0034-3, PMID: 39743787

[ref8] Adam M. Heuer H. Hallmann J. (2014). Bacterial antagonists of fungal pathogens also control root-knot nematodes by induced systemic resistance of tomato plants. PLoS One 9:e90402. doi: 10.1371/journal.pone.0090402, PMID: 24587352 PMC3938715

[ref9] Adiwena M. Murtilaksono A. Egra S. Hoesain M. Asyiah I. N. Pradana A. P.. (2023). The effects of micronutrient-enriched media on the efficacy of *Bacillus subtilis* as biological control agent against *Meloidogyne incognita*. Biodiv. J. Biol. Divers. 24, 33–39. doi: 10.13057/biodiv/d240105, PMID: 39204774

[ref10] Ahmad G. Khan A. Khan A. A. Ali A. Mohhamad H. I. (2021). Biological control: a novel strategy for the control of the plant parasitic nematodes. Antonie Van Leeuwenhoek 114, 885–912. doi: 10.1007/s10482-021-01577-9, PMID: 33893903

[ref11] Ahmed S. Liu Q. Jian H. (2019). *Bacillus cereus* a potential strain infested cereal cyst nematode (*Heterodera avenae*). Pak. J. Nematol. 37, 53–61. doi: 10.18681/pjn.v37.i01.p53-61

[ref12] Ali Q. Yu C. Wang Y. Sheng T. Zhao X. Wu X.. (2023). High killing rate of nematode and promotion of rice growth by synthetic volatiles from *Bacillus* strains due to enhanced oxidative stress response. Physiol. Plant. 175:e13868. doi: 10.1111/ppl.13868, PMID: 36724171

[ref13] Amorim D. J. Tsujimoto T. F. Baldo F. B. Leite L. G. Harakava R. Wilcken S. R. S.. (2024). *Bacillus*, Pseudomonas and Serratia control *Meloidogyne incognita* (Rhabditida: Meloidogynidae) and promote the growth of tomato plants. Rhizosphere 31:100935. doi: 10.1016/j.rhisph.2024.100935

[ref14] Antil S. Kumar R. Pathak D. V. Kumar A. Panwar A. Kumari A. (2022a). Plant growth-promoting rhizobacteria-*Bacillus cereus* KMT-5 and *B. megaterium* KMT-8 effectively suppressed *Meloidogyne javanica* infection. Appl. Soil Ecol. 174:104419. doi: 10.1016/j.apsoil.2022.104419

[ref15] Antil S. Kumar R. Pathak D. V. Kumar A. Panwar A. Kumari A.. (2021). On the potential of *Bacillus aryabhattai* KMT-4 against *Meloidogyne javanica*. Egypt. J. Biol. Pest Control 31, 1–9. doi: 10.1186/s41938-021-00417-2, PMID: 39743787

[ref16] Antil S. Kumar R. Pathak D. V. Kumar A. Panwar A. Kumari A.. (2022b). Potential of *Bacillus altitudinis* KMS-6 as a biocontrol agent of *Meloidogyne javanica*. J. Pest. Sci. 95, 1443–1452. doi: 10.1007/s10340-021-01469-x

[ref17] Asaturova A. M. Bugaeva L. N. Homyak A. I. Slobodyanyuk G. A. Kashutina E. V. Yasyuk L. V.. (2022). *Bacillus velezensis* strains for protecting cucumber plants from root-knot nematode *Meloidogyne incognita* in a greenhouse. Plan. Theory 11:275. doi: 10.3390/plants11030275, PMID: 35161255 PMC8838184

[ref18] Asif K. Shabaan M. Mahmood W. Asghar H. N. Zahir Z. A. Zulfiqar U.. (2024). Synergistic application of bacterial consortium and organic amendments improves the growth and seed quality of mash bean (*Vigna Mungo* L.). Soil Sci. Plant Nutr. 24, 6893–6905. doi: 10.1007/s42729-024-02012-4, PMID: 39744507

[ref19] Ayaz M. Ali Q. Farzand A. Khan A. R. Ling H. Gao X. (2021). Nematicidal volatiles from *Bacillus atrophaeus* GBSC56 promote growth and stimulate induced systemic resistance in tomato against *Meloidogyne incognita*. Int. J. Mol. Sci. 22:5049. doi: 10.3390/ijms22095049, PMID: 34068779 PMC8126219

[ref20] Ayaz M. Li C. H. Ali Q. Zhao W. Chi Y. K. Shafiq M.. (2023). Bacterial and fungal biocontrol agents for plant disease protection: journey from lab to field, current status, challenges, and global perspectives. Molecules 28:6735. doi: 10.3390/molecules28186735, PMID: 37764510 PMC10537577

[ref21] Bacon C. W. Hinton D. M. Hinton A. Jr. (2006). Growth-inhibiting effects of concentrations of fusaric acid on the growth of *Bacillus mojavensis* and other biocontrol Bacillus species. J. Appl. Microbiol. 100, 185–194. doi: 10.1111/j.1365-2672.2005.02770.x, PMID: 16405699

[ref22] Banihashemian S. N. Jamali S. Golmohammadi M. Ghasemnezhad M. (2023). Management of root-knot nematode in kiwifruit using resistance-inducing *Bacillus altitudinis*. Trop. Plant Pathol. 48, 443–451. doi: 10.1007/s40858-023-00573-w

[ref23] Baptista J. P. Teixeira G. M. Jesus M. L. A. Bertê R. Higashi A. Mosella M.. (2022). Antifungal activity and genomic characterization of the isolate *Bacillus velezensis* CMRP 4489, a biocontrol agent for plant-pathogenic fungi. Res. Sq. doi: 10.21203/rs.3.rs-1619465/v1 PMC9579199 36257999

[ref24] Barnawal D. Bharti N. Pandey S. S. Pandey A. Chanotiya C. S. Kalra A. (2017). Plant growth-promoting rhizobacteria enhance wheat salt and drought stress tolerance by altering endogenous phytohormone levels and TaCTR1/TaDREB2 expression. Physiol. Plant. 161, 502–514. doi: 10.1111/ppl.12614, PMID: 28786221

[ref25] Basiouny A. G. Abo-Zaid G. A. (2018). Biocontrol of the root-knot nematode, *Meloidogyne incognita*, using an eco-friendly formulation from *Bacillus subtilis*, lab and greenhouse studies. Egypt. J. Biol. Pest Control 28:87. doi: 10.1186/s41938-018-0094-4

[ref26] Bavaresco L. G. Guaberto L. M. Araujo F. F. (2021). Interaction of *Bacillus subtilis* with resistant and susceptible tomato (*Solanum lycopersicum* L.) in the control of *Meloidogyne incognita*. Arch. Phytopathol. Plant Protect. 54, 359–374. doi: 10.1080/03235408.2020.1833279

[ref27] Behl K. Jaiswal P. Pabbi S. (2024). Recent advances in microbial and nano-formulations for effective delivery and agriculture sustainability. Biocatal. Agric. Biotechnol. 58:103180. doi: 10.1016/j.bcab.2024.103180

[ref28] Berlitz D. L. Knaak N. Cassal M. C. Fiuza L. M. (2014). “*Bacillus* and biopesticides in control of phytonematodes” in Basic and applied aspects of biopesticides. ed. Sahayaraj K. (New Delhi: Springer).

[ref29] Bhat A. A. Shakeel A. Waqar S. Handoo Z. A. Khan A. A. (2023). Microbes vs. nematodes: insights into biocontrol through antagonistic organisms to control root-knot nematodes. Plan. Theory 12:451. doi: 10.3390/plants12030451, PMID: 36771535 PMC9919851

[ref30] Blouin M. S. Yowell C. A. Courtney C. H. Dame J. B. (1998). Substitution bias, rapid saturation, and the use of mtDNA for nematode systematics. Mol. Biol. Evol. 15, 1719–1727. doi: 10.1093/oxfordjournals.molbev.a025898, PMID: 9866206

[ref31] Bo T. Kong C. Zou S. Mo M. Liu Y. (2022). *Bacillus nematocida* B16 enhanced the rhizosphere colonization of *Pochonia chlamydosporia* ZK7 and controlled the efficacy of the root-knot nematode *Meloidogyne incognita*. Microorganisms 10:218. doi: 10.3390/microorganisms10020218, PMID: 35208675 PMC8879550

[ref33] Borriss R. Wu H. Gao X. (2019). “Secondary metabolites of the plant growth promoting model rhizobacterium *Bacillus velezensis* FZB42 are involved in direct suppression of plant pathogens and in stimulation of plant-induced systemic resistance” in Secondary metabolites of plant growth promoting rhizomicroorganisms: discovery and applications. eds. Singh H. Keswani C. Reddy M. Sansinenea E. García-Estrada C.. 147–168.

[ref34] Bravo A. Gill S. S. Soberón M. (2007). Mode of action of *Bacillus thuringiensis* cry and Cyt toxins and their potential for insect control. Toxicon 49, 423–435. doi: 10.1016/j.toxicon.2006.11.022, PMID: 17198720 PMC1857359

[ref35] Brzezinska M. S. Kalwasińska A. Świątczak J. Żero K. Jankiewicz U. (2020). Exploring the properties of chitinolytic *Bacillus* isolates for the pathogens biological control. Microb. Pathog. 148:104462. doi: 10.1016/j.micpath.2020.104462, PMID: 32835774

[ref36] Bui H. X. Hadi B. A. Oliva R. Schroeder N. E. (2020). Beneficial bacterial volatile compounds for the control of root-knot nematode and bacterial leaf blight on rice. Crop Prot. 135:104792. doi: 10.1016/j.cropro.2019.04.016

[ref37] Butu M. Rodino S. Butu A. (2022). Biopesticide formulations-current challenges and future perspectives. Biopesticides 2, 19–29. doi: 10.1016/B978-0-12-823355-9.00010-9, PMID: 39743835

[ref38] Calvo P. Ormeño-Orrillo E. Martínez-Romero E. Zúñiga D. (2010). Characterization of Bacillus isolates of potato rhizosphere from Andean soils of Peru and their potential PGPR characteristics. Braz. J. Microbiol. 41, 899–906. doi: 10.1590/S1517-83822010000400008, PMID: 24031569 PMC3769774

[ref39] Camacho M. de Los Santos B. Vela M. D. Talavera M. (2023). Use of bacteria isolated from berry rhizospheres as biocontrol agents for charcoal rot and root-knot nematode strawberry diseases. Horticulturae 9:346. doi: 10.3390/horticulturae9030346

[ref40] Camatti G. dos Santos F. M. Júnior G. L. D. S. R. Camargo D. P. Manfio G. S. Santos J. R. P.. (2023). *Bacillus*-and *Trichoderma*-based products control the spiral nematode *Helicotylenchus dihystera* in soybean. Rhizosphere 27:100717. doi: 10.1016/j.rhisph.2023.100717

[ref41] Carmona-Hernandez S. Reyes-Pérez J. J. Chiquito-Contreras R. G. Rincon-Enriquez G. Cerdan-Cabrera C. R. Hernandez-Montiel L. G. (2019). Biocontrol of postharvest fruit fungal diseases by bacterial antagonists: a review. Agronomy 9:121. doi: 10.3390/agronomy9030121

[ref9001] Carriel C. B. Soto D. V. (2022). Persistence of Bacillus thuringiensis and Bacillus pumilus potential biological control agents of the coffee berry borer under field conditions of Puerto Rico. Sci. Agric. 19, 43–56. doi: 10.19053/01228420.v19.n3.2022.14685

[ref42] Castillo H. F. Reyes C. F. Morales G. G. Herrera R. R. Aguilar C. (2013). “Biological control of root pathogens by plant growth promoting Bacillus spp” in Weed and pest control - conventional and new challenges. eds. Soloneski S. Larramendy M. L. (Rijeka, Croatia: InTech), 79–103.

[ref43] Cavalcanti V. P. Terra W. C. de Souza J. T. Pacheco P. V. M. de Sousa L. F. Belizario R. A.. (2024). A commercial formulation of *Bacillus subtilis* induces metabolomic changes in root exudates that invert the chemotactic responses of the nematode *Meloidogyne incognita* to host and non-host plants. J. Plant Dis. Protect. 131, 899–909. doi: 10.1007/s41348-024-00892-3

[ref44] Cetintas R. Kusek M. Fateh S. A. (2018). Effect of some plant growth-promoting rhizobacteria strains on root-knot nematode, *Meloidogyne incognita*, on tomatoes. Egypt. J. Biol. Pest Control 28, 1–5. doi: 10.1186/s41938-017-0008-x

[ref45] Chavarria-Quicaño E. Contreras-Jácquez V. Carrillo-Fasio A. De la Torre-González F. Asaff-Torres A. (2023a). Native *Bacillus paralicheniformis* isolate as a potential agent for phytopathogenic nematodes control. Front. Microbiol. 14:1213306. doi: 10.3389/fmicb.2023.1213306, PMID: 37588888 PMC10425774

[ref46] Chavarria-Quicaño E. De la Torre-González F. González-Riojas M. Rodríguez-González J. Asaff-Torres A. (2023b). Nematicidal lipopeptides from *Bacillus paralicheniformis* and *Bacillus subtilis*: a comparative study. Appl. Microbiol. Biotechnol. 107, 1537–1549. doi: 10.1007/s00253-023-12391-w, PMID: 36719435

[ref47] Chen J. Abawi G. S. Zuckerman B. M. (2000). Efficacy of *Bacillus thuringiensis*, *Paecilomyces marquandii*, and *Streptomyces costaricanus* with and without organic amendments against *Meloidogyne hapla* infecting lettuce. J. Nematol. 32, 70–77, PMID: . Available at: https://pubmed.ncbi.nlm.nih.gov/19270951/19270951 PMC2620431

[ref48] Chen L. Wang Y. Zhu L. Min Y. Tian Y. Gong Y.. (2024). 3-(Methylthio) propionic acid from *Bacillus thuringiensis* Berliner exhibits high Nematicidal activity against the root knot nematode *Meloidogyne incognita* (Kofoid and white) Chitwood. Int. J. Mol. Sci. 25:1708. doi: 10.3390/ijms25031708, PMID: 38338986 PMC10855422

[ref49] Chernov T. I. Semenov M. V. (2021). Management of soil microbial communities: opportunities and prospects (a review). Eurasian Soil Sci. 54, 1888–1902. doi: 10.1134/S1064229321120024

[ref50] Chien Y. C. Huang C. H. (2020). Biocontrol of bacterial spot on tomato by foliar spray and growth medium application of *Bacillus amyloliquefaciens* and *Trichoderma asperellum*. Eur. J. Plant Pathol. 156, 995–1003. doi: 10.1007/s10658-020-01947-5

[ref51] Chinheya C. C. Yobo K. S. Laing M. D. (2017). Biological control of the rootknot nematode, *Meloidogyne javanica* (Chitwood) using *Bacillus* isolates, on soybean. Biol. Control 109, 37–41. doi: 10.1016/j.biocontrol.2017.03.009

[ref52] Cho W. I. Chung M. S. (2020). Bacillus spores: A review of their properties and inactivation processing technologies. Food Sci. Biotechnol. 29, 1447–1461. doi: 10.1007/s10068-020-00809-4, PMID: 33041624 PMC7538368

[ref53] Choi T. G. Maung C. E. H. Lee D. R. Henry A. B. Lee Y. S. Kim K. Y. (2020). Role of bacterial antagonists of fungal pathogens, *Bacillus thuringiensis* KYC and *Bacillus velezensis* CE 100 in control of root-knot neatode, *Meloidogyne incognita* and subsequent growth promotion of tomato. Biocontrol Sci. Tech. 30, 685–700. doi: 10.1080/09583157.2020.1765980

[ref54] Choudhary D. K. Johri B. N. (2009). Interactions of *Bacillus* spp. and plants–with special reference to induced systemic resistance (ISR). Microbiol. Res. 164, 493–513. doi: 10.1016/j.micres.2008.08.007, PMID: 18845426

[ref55] Chowdhury S. P. Hartmann A. Gao X. Borriss R. (2015). Biocontrol mechanism by root-associated *Bacillus amyloliquefaciens* FZB42–a review. Front. Microbiol. 6:780. doi: 10.3389/fmicb.2015.00780, PMID: 26284057 PMC4517070

[ref56] Cruz‐Magalhães V. Guimarães R. A. Da Silva J. C. de Faria A. F. Pedroso M. P. Campos V. P.. (2022). The combination of two Bacillus strains suppresses *Meloidogyne incognita* and fungal pathogens, but does not enhance plant growth. Pest Manag. Sci. 78, 722–732. doi: 10.1002/ps.6685, PMID: 34689397

[ref57] Dai M. M. Liu R. Jiang H. Zhang X. P. Song W. W. Zhang J.. (2023). Volatile organic compounds of *Bacillus pumilus* strain S1-10 exhibit fumigant activity against *Meloidogyne incognita*. Plant Dis. 107, 3057–3063. doi: 10.1094/PDIS-10-22-2391-RE, PMID: 36916837

[ref58] Danilova I. V. Vasileva I. A. Gilmutdinova A. I. Dyadkina I. V. Khusnullina L. K. Khasanov D. I.. (2023). Characterization of *Bacillus pumilus* strains with targeted gene editing for antimicrobial peptides and sporulation factor. Microorganisms 11:1508. doi: 10.3390/microorganisms11061508, PMID: 37375011 PMC10303315

[ref59] Das K. Ayim B. Y. Borodynko-Filas N. Das S. C. Aminuzzaman F. M. (2023). Genome editing (CRISPR/Cas9) in plant disease management: challenges and future prospects. J. Plant Protect. Res. 63, 159–172. doi: 10.24425/jppr.2023.145761

[ref60] Das S. Wadud M. A. Khokon M. A. R. (2021). Functional evaluation of culture filtrates of *Bacillus subtilis* and *Pseudomonas fluorescens* on the mortality and hatching of *Meloidogyne javanica*. Saudi J. Biol. Sci. 28, 1318–1323. doi: 10.1016/j.sjbs.2020.11.055, PMID: 33613061 PMC7878824

[ref61] Daulagala P. W. H. K. P. (2021). Chitinolytic endophytic bacteria as biocontrol agents for phytopathogenic fungi and nematode pests: a review. Asian J. Res. Bot. 5, 14–24.

[ref62] de Paula L. L. Campos V. P. Terra W. C. de Brum D. Jacobs D. C. Bui H. X.. (2024). The combination of *Bacillus amyloliquefaciens* and *Purpureocillium lilacinum* in the control of *Meloidogyne enterolobii*. Biol. Control 189:105438. doi: 10.1016/j.biocontrol.2023.105438

[ref63] d'Errico G. Marra R. Crescenzi A. Davino S. W. Fanigliulo A. Woo S. L.. (2019). Integrated management strategies of *Meloidogyne incognita* and *Pseudopyrenochaeta lycopersici* on tomato using a *Bacillus firmus*-based product and two synthetic nematicides in two consecutive crop cycles in greenhouse. Crop Prot. 122, 159–164. doi: 10.1016/j.cropro.2019.05.004

[ref64] Devindrappa M. Kamra A. Singh D. Gawade B. Sirohi A. (2023). Plant growth promoting *Bacillus* species elicit defense against *Meloidogyne incognita* infecting tomato in polyhouse. J. Basic Microbiol. 2023, 1–9. doi: 10.22541/au.168001566.62776546/v1 37528495

[ref65] Díaz-Manzano F. E. Amora D. X. Martínez-Gómez Á. Moelbak L. Escobar C. (2023). Biocontrol of *Meloidogyne* spp. in *Solanum lycopersicum* using a dual combination of *Bacillus* strains. Front. Plant Sci. 13:1077062. doi: 10.3389/fpls.2022.1077062, PMID: 36684755 PMC9846617

[ref66] Diyapoglu A. Oner M. Meng M. (2022). Application potential of bacterial volatile organic compounds in the control of root-knot nematodes. Molecules 27:4355. doi: 10.3390/molecules27144355, PMID: 35889228 PMC9318376

[ref67] Dobrzyński J. Jakubowska Z. Kulkova I. Kowalczyk P. Kramkowski K. (2023). Biocontrol of fungal phytopathogens by *Bacillus pumilus*. Front. Microbiol. 14:1194606. doi: 10.3389/fmicb.2023.1194606, PMID: 37560520 PMC10407110

[ref68] Du J. Gao Q. Ji C. Song X. Liu Y. Li H.. (2022). *Bacillus licheniformis* JF-22 to control *Meloidogyne incognita* and its effect on tomato rhizosphere microbial community. Front. Microbiol. 13:863341. doi: 10.3389/fmicb.2022.863341, PMID: 35464941 PMC9022077

[ref69] Efthimiadou A. Katsenios N. Chanioti S. Giannoglou M. Djordjevic N. Katsaros G. (2020). Effect of foliar and soil application of plant growth promoting bacteria on growth, physiology, yield and seed quality of maize under Mediterranean conditions. Sci. Rep. 10:21060. doi: 10.1038/s41598-020-78034-6, PMID: 33273634 PMC7713431

[ref70] El Aimani A. Houari A. Laasli S. E. Mentag R. Iraqi D. Diria G.. (2022). Antagonistic potential of Moroccan entomopathogenic nematodes against root-knot nematodes, *Meloidogyne javanica* on tomato under greenhouse conditions. Sci. Rep. 12:2915. doi: 10.1038/s41598-022-07039-0, PMID: 35190634 PMC8861030

[ref71] Elango K. Sobhana E. Sujithra P. Bharath D. Ahuja A. (2020). Traditional agricultural practices as a tool for management of insects and nematode pests of crops: an overview. J. Entomol. Zool. Stud. 8, 237–245.

[ref72] El-Saadony M. T. Abuljadayel D. A. Shafi M. E. Albaqami N. M. Desoky E. S. M. El-Tahan A. M.. (2021). Control of foliar phytoparasitic nematodes through sustainable natural materials: current progress and challenges. Saudi J. Biol. Sci. 28, 7314–7326. doi: 10.1016/j.sjbs.2021.08.035, PMID: 34867034 PMC8626253

[ref73] El-Sawy S. El-Nagdi W. Mohamed S. Khalil B. Soliman G. (2023). The efficiency of biofertilizer and bio-control on root-knot nematode, using bacterial strains, and its effect on tomato plant protein patterns, and improving yield under field conditions. Res. Sq., 2–37. doi: 10.21203/rs.3.rs-3475183/v1

[ref74] Engelbrecht G. Claassens S. Mienie C. M. Fourie H. (2022). Filtrates of mixed *Bacillus* spp inhibit second-stage juvenile motility of root-knot nematodes. Rhizosphere 22:100528. doi: 10.1016/j.rhisph.2022.100528

[ref75] Engelbrecht G. van Rensburg P. J. J. Fourie H. Claassens S. (2020). *In vitro* bioassays to determine the effect of *Bacillus soli* filtrates on the paralysis of *Meloidogyne incognita* second-stage juveniles. Nematology 22, 239–243. doi: 10.1163/15685411-00003345

[ref76] Esitken A. H. M. E. T. Karlidag H. Ü. S. E. Y. İ. N. Ercisli S. E. Z. A. İ. Sahin F. İ. K. R. E. T. T. İ. N. (2002). Effects of foliar application of *Bacillus subtilis* Osu-142 on the yield, growth and control of shot-hole disease (Coryneum blight) of apricot. Gartenbauwissenschaft 67, 139–142.

[ref77] Etesami H. Jeong B. R. Glick B. R. (2023). Biocontrol of plant diseases by *Bacillus* Spp. Physiol. Mol. Plant Pathol. 126:102048. doi: 10.1016/j.pmpp.2023.102048

[ref78] Fabiyi O. A. (2024). “Application of *Bacillus* species in the Management of *Meloidogyne incognita*” in Sustainable Management of Nematodes in agriculture, role of microbes-assisted strategies, vol. 19 (Cham: Springer International Publishing), 249–264.

[ref79] Fallahzadeh-Mamaghani V. Shahbazi-Ezmareh R. Shirzad A. Moslehi S. (2023). Possible mechanisms of action of *Bacillus wiedmannii* AzBw1, a biocontrol agent of the root-knot nematode, *Meloidogyne arenaria*. Egypt. J. Biol. Pest Control 33:28. doi: 10.1186/s41938-023-00668-1

[ref80] Forghani F. Hajihassani A. (2020). Recent advances in the development of environmentally benign treatments to control root-knot nematodes. Front. Plant Sci. 11:1125. doi: 10.3389/fpls.2020.01125, PMID: 32793271 PMC7387703

[ref81] Fu H. Z. Marian M. Enomoto T. Hieno A. Ina H. Suga H.. (2020). Biocontrol of tomato bacterial wilt by foliar spray application of a novel strain of endophytic *Bacillus* sp. Microbes Environ. 35:p.ME20078. doi: 10.1264/jsme2.ME20078, PMID: 33012743 PMC7734409

[ref82] Galbieri R. Oliveira J. A. D. Negri B. F. Boldt A. S. Rizzi U. D. S. Belot J. L. (2023). *Bacillus subtilis* as growth-promoting rhizobacteria co-inoculated on *Bradyrhizobium*-treated soybean seeds in the planting furrow. Rev. Ceres 70:e70601. doi: 10.1590/0034-737X202370060001

[ref83] Gamalero E. Glick B. R. (2020). The use of plant growth-promoting bacteria to prevent nematode damage to plants. Biology 9:381. doi: 10.3390/biology9110381, PMID: 33171782 PMC7695023

[ref84] Gao A. Zheng L. Wang S. Pan H. Zhang H. (2024). Preparation of microcapsules and evaluation of their biocontrol efficacy. J. Biosci. Bioeng. 138, 328–337. doi: 10.1016/j.jbiosc.2024.05.007, PMID: 38997872

[ref85] Gassmann A. J. Stock S. P. Sisterson M. S. Carrière Y. Tabashnik B. E. (2008). Synergism between entomopathogenic nematodes and *Bacillus thuringiensis* crops: integrating biological control and resistance management. J. Appl. Ecol. 45, 957–966. doi: 10.1111/j.1365-2664.2008.01457.x

[ref86] Gattoni K. M. Park S. W. Lawrence K. S. (2022). Evaluation of the mechanism of action of bacillus spp. to manage meloidogyne incognita with split root assay, RT-qPCR and qPCR. Front. Plant Sci. 13:1079109. doi: 10.3389/fpls.2022.1079109, PMID: 36743572 PMC9895862

[ref87] Geng C. Liu Y. Li M. Tang Z. Muhammad S. Zheng J.. (2017). Dissimilar crystal proteins Cry5Ca1 and Cry5Da1 synergistically act against *Meloidogyne incognita* and delay Cry5Ba-based nematode resistance. Appl. Environ. Microbiol. 83, e03505–e03516. doi: 10.1128/AEM.03505-16, PMID: 28710264 PMC5583498

[ref88] Ghahremani Z. Escudero N. Beltrán-Anadón D. Saus E. Cunquero M. Andilla J.. (2020). *Bacillus firmus* strain I-1582, a nematode antagonist by itself and through the plant. Front. Plant Sci. 11:796. doi: 10.3389/fpls.2020.00796, PMID: 32765537 PMC7381289

[ref89] Gill S. S. Cowles E. A. Pietrantonio P. V. (1992). The mode of action of *Bacillus thuringiensis* endotoxins. Annu. Rev. Entomol. 37, 615–634. doi: 10.1146/annurev.en.37.010192.003151 1311541

[ref90] Gomaa E. Z. (2021). Microbial chitinases: properties, enhancement and potential applications. Protoplasma 258, 695–710. doi: 10.1007/s00709-021-01612-6, PMID: 33483852

[ref91] Gotor-Vila A. Usall J. Torres R. Solsona C. Teixidó N. (2019). Enhanced shelf-life of the formulated biocontrol agent *Bacillus amyloliquefaciens* CPA-8 combining diverse packaging strategies and storage conditions. Int. J. Food Microbiol. 290, 205–213. doi: 10.1016/j.ijfoodmicro.2018.10.013, PMID: 30366262

[ref92] Grage K. McDermott P. Rehm B. H. (2017). Engineering *Bacillus megaterium* for production of functional intracellular materials. Microb. Cell Factories 16, 211–212. doi: 10.1186/s12934-017-0823-5, PMID: 29166918 PMC5700737

[ref93] Griffitts J. S. Whitacre J. L. Stevens D. E. Aroian R. V. (2005). Bt toxin resistance from loss of a putative carbohydrate-modifying enzyme. Science 293, 860–864. doi: 10.1126/science.1062441, PMID: 11486087

[ref94] Grubišić D. Uroić G. Ivošević A. Grdiša M. (2018). Nematode control by the use of antagonistic plants. Agric. Conspec. Sci. 83, 269–275. Available at: https://hrcak.srce.hr/207925

[ref95] Guo S. Liu M. Peng D. Ji S. Wang P. Yu Z.. (2008). New strategy for isolating novel nematicidal crystal protein genes from *Bacillus thuringiensis* strain YBT-1518. Appl. Environ. Microbiol. 74, 6997–7001. doi: 10.1128/AEM.01346-08, PMID: 18820056 PMC2583473

[ref96] Guo Y. Weng M. Sun Y. Carballar-Lejarazú R. Wu S. Lian C. (2022). *Bacillus thuringiensis* toxins with nematocidal activity against the pinewood nematode *Bursaphelenchus xylophilus*. J. Invertebr. Pathol. 189:107726. doi: 10.1016/j.jip.2022.107726, PMID: 35122837

[ref97] Gupta R. Mfarrej M. Elnour R. Hashem M. Ahmad F. (2023). Defence response of host plants for cyst nematode: a review on parasitism and defence. Science 35:102829:102829. doi: 10.1016/j.jksus.2023.102829, PMID: 39743835

[ref98] Gurikar C. Gowda N. N. Hanumantharaju K. N. Netravati B. P. (2022). “Role of *Bacillus* species in soil fertility with reference to rhizosphere engineering” in Rhizosphere engineering (Amsterdam, Netherlands: Elsevier), 65–76.

[ref99] Habazar T. Yanti Y. Dani M. R. Monica D. (2021). “Biocontrol of *Meloidogyne* sp. on tomato plants by selected *Bacillus* spp” in IOP Conference Series: Earth and Environmental Science (Bristol, United Kingdom: IOP Publishing). 757:012019.

[ref100] Hamze R. Ruiu L. (2022). *Brevibacillus laterosporus* as a natural biological control agent of soil-dwelling nematodes. Agronomy 12:2686. doi: 10.3390/agronomy12112686

[ref101] Hartz P. Gehl M. König L. Bernhardt R. Hannemann F. (2021). Development and application of a highly efficient CRISPR-Cas9 system for genome engineering in *Bacillus megaterium*. J. Biotechnol. 329, 170–179. doi: 10.1016/j.jbiotec.2021.02.006, PMID: 33600891

[ref102] Hayat H. S. Rehman A. U. Farooq S. Naveed M. Ali H. M. Hussain M. (2023). Boron seed coating combined with seed inoculation with boron tolerant bacteria (*Bacillus* sp. MN-54) and maize stalk biochar improved growth and productivity of maize (*Zea mays* L.) on saline soil. Heliyon 9:e22075. doi: 10.1016/j.heliyon.2023.e22075, PMID: 38034772 PMC10682679

[ref103] He Y. Wang R. Zhao H. Ren Y. Agarwal M. Zheng D.. (2022). Predicting potential global distribution and risk regions for potato cyst nematodes (*Globodera rostochiensis* and *Globodera pallida*). Sci. Rep. 12:21843. doi: 10.1038/s41598-022-26443-0, PMID: 36528656 PMC9759053

[ref104] Heerklotz H. Seelig J. (2007). Leakage and lysis of lipid membranes induced by the lipopeptide surfactin. Eur. Biophys. J. 36, 305–314. doi: 10.1007/s00249-006-0091-5, PMID: 17051366

[ref105] Henry G. Deleu M. Jourdan E. Thonart P. Ongena M. (2011). The bacterial lipopeptide surfactin targets the lipid fraction of the plant plasma membrane to trigger immune-related responses. Cell. Microbiol. 13, 1824–1837. doi: 10.1111/j.1462-5822.2011.01664.x, PMID: 21838773

[ref106] Hezakiel H. E. Thampi M. Rebello S. Sheikhmoideen J. M. (2024). Biopesticides: a green approach towards agricultural pests. Appl. Biochem. Biotechnol. 196, 5533–5562. doi: 10.1007/s12010-023-04765-7, PMID: 37994977

[ref107] Hossain M. A. Hossain M. S. Akter M. (2023). Challenges faced by plant growth-promoting bacteria in field-level applications and suggestions to overcome the barriers. Physiol. Mol. Plant Pathol. 126:102029. doi: 10.1016/j.pmpp.2023.102029

[ref108] Hsiao C. Y. Blanco S. D. Peng A. L. Fu J. Y. Chen B. W. Luo M. C.. (2023). Seed treatment with calcium carbonate containing *Bacillus amyloliquefaciens* PMB05 powder is an efficient way to control black rot disease of cabbage. Agriculture 13:926. doi: 10.3390/agriculture13050926

[ref109] Hu H. J. Chen Y. L. Wang Y. F. Tang Y. Y. Chen S. L. Yan S. Z. (2017). Endophytic *Bacillus cereus* effectively controls *Meloidogyne incognita* on tomato plants through rapid rhizosphere occupation and repellent action. Plant Dis. 101, 448–455. doi: 10.1094/PDIS-06-16-0871-RE, PMID: 30677349

[ref110] Hu H. Gao Y. Li X. Chen S. Yan S. Tian X. (2020). Identification and nematicidal characterization of proteases secreted by endophytic bacteria *Bacillus cereus* BCM2. Phytopathology 110, 336–344. doi: 10.1094/PHYTO-05-19-0164-R, PMID: 31524559

[ref111] Hu L. B. Shi Z. Q. Zhang T. Yang Z. M. (2007). Fengycin antibiotics isolated from B-FS01 culture inhibit the growth of *fusarium moniliforme* Sheldon ATCC 38932. FEMS Microbiol. Lett. 272, 91–98. doi: 10.1111/j.1574-6968.2007.00743.x, PMID: 17490402

[ref112] Hu Y. You J. Wang Y. Long Y. Wang S. Pan F.. (2022). Biocontrol efficacy of *Bacillus velezensis* strain YS-AT-DS1 against the root-knot nematode *Meloidogyne incognita* in tomato plants. Front. Microbiol. 13:1035748. doi: 10.3389/fmicb.2022.1035748, PMID: 36483201 PMC9722970

[ref113] Huang M. Bulut A. Shrestha B. Matera C. Grundler F. M. Schleker A. S. S. (2021). *Bacillus firmus* I-1582 promotes plant growth and impairs infection and development of the cyst nematode *Heterodera schachtii* over two generations. Sci. Rep. 11:14114. doi: 10.1038/s41598-021-93567-0, PMID: 34239009 PMC8266893

[ref114] Huang X. W. Niu Q. H. Zhou W. Zhang K. Q. (2005). *Bacillus nematocida* sp. nov., a novel bacterial strain with nematotoxic activity isolated from soil in Yunnan, China. Syst. Appl. Microbiol. 28, 323–327. doi: 10.1016/j.syapm.2005.01.008, PMID: 15997705

[ref115] Huang X. Wei Z. Zhao G. Gao X. Yang S. Cui Y. (2008). Optimization of sterilization of *Escherichia coli* in milk by surfactin and fengycin using a response surface method. Curr. Microbiol. 56, 376–381. doi: 10.1007/s00284-007-9066-8, PMID: 18058172

[ref116] Huang Y. Xu C. Ma L. Zhang K. Duan C. Mo M. (2010). Characterisation of volatiles produced from *Bacillus megaterium* YFM3. 25 and their nematicidal activity against *Meloidogyne incognita*. Eur. J. Plant Pathol. 126, 417–422. doi: 10.1007/s10658-009-9550-z

[ref117] Hui F. Scheib U. Hu Y. Sommer R. J. Aroian R. V. Ghosh P. (2012). Structure and glycolipid binding properties of the nematicidal protein Cry5B. Biochemistry 51, 9911–9921. doi: 10.1021/bi301386q, PMID: 23150986 PMC3567309

[ref118] Iftikhar Y. Sajid A. Shakeel Q. Ahmad Z. Ul Haq Z. (2020). “Biological antagonism: a safe and sustainable way to manage plant diseases” in Plant disease management strategies for sustainable agriculture through traditional and modern approaches: sustainability in plant and crop protection. eds. Ul Haq I. Ijaz S. (Cham: Springer).

[ref119] Jaiswal D. K. Gawande S. J. Soumia P. S. Krishna R. Vaishnav A. Ade A. B. (2022). Biocontrol strategies: an eco-smart tool for integrated pest and diseases management. BMC Microbiol. 22:324. doi: 10.1186/s12866-022-02744-2, PMID: 36581846 PMC9801620

[ref120] Jamal Q. Cho J. Y. Moon J. H. Munir S. Anees M. Kim K. Y. (2017). Identification for the first time of Cyclo (d-pro-l-Leu) produced by *Bacillus amyloliquefaciens* Y1 as a Nematocide for control of *Meloidogyne incognita*. Molecules 22:1839. doi: 10.3390/molecules22111839, PMID: 29077011 PMC6150376

[ref121] Jang S. Choi S. K. Zhang H. Zhang S. Ryu C. M. Kloepper J. W. (2023). History of a model plant growth-promoting rhizobacterium, *Bacillus velezensis* GB03: from isolation to commercialization. Front. Plant Sci. 14:1279896. doi: 10.3389/fpls.2023.1279896, PMID: 37885658 PMC10598611

[ref122] Jeong M. H. Yang S. Y. Lee Y. S. Ahn Y. S. Park Y. S. Han H. R.. (2015). Selection and characterization of *Bacillus licheniformis* MH48 for the biocontrol of pine wood nematode (*Bursaphelenchus xylophilus*). J. Korean Soc. Forest Sci. 104, 512–518. doi: 10.14578/jkfs.2015.104.3.512

[ref123] Jiang H. Tian L. Bu F. Sun Q. Zhao X. Han Y. (2021). RNA-seq-based identification of potential resistance genes against the soybean cyst nematode (*Heterodera glycines*) HG type 1.2.3.5.7 in ‘Dongnong L-10’. Physiol. Mol. Plant Pathol. 114:101627. doi: 10.1016/j.pmpp.2021.101627

[ref124] Jouzani G. S. Valijanian E. Sharafi R. (2017). *Bacillus thuringiensis*: a successful insecticide with new environmental features and tidings. Appl. Microbiol. Biotechnol. 101, 2691–2711. doi: 10.1007/s00253-017-8175-y, PMID: 28235989

[ref125] Jung W. J. Jung S. J. An K. N. Jin Y. L. Park R. D. Kim K. Y.. (2002). Effect of chitinase-producing *Paenibacillus illinoisensis* KJA-424 on egg hatching of root-knot nematode (*Meloidogyne incognita*). J. Microbiol. Biotechnol. 12, 865–871. Available at: https://koreascience.kr/ksci/search/article/articleView.ksci?articleBean.atclMgntNo=E1MBA4_2002_v12n6_865

[ref126] Kahn T. W. Duck N. B. McCarville M. T. Schouten L. C. Schweri K. Zaitseva J.. (2021). A *Bacillus thuringiensis* cry protein controls soybean cyst nematode in transgenic soybean plants. Nat. Commun. 12:3380. doi: 10.1038/s41467-021-23743-3, PMID: 34099714 PMC8184815

[ref127] Kamalanathan V. Sevugapperumal N. Nallusamy S. (2023). Antagonistic bacteria *Bacillus velezensis* VB7 possess nematicidal action and induce an immune response to suppress the infection of root-knot nematode (RKN) in tomato. Genes 14:1335. doi: 10.3390/genes14071335, PMID: 37510240 PMC10378951

[ref128] Kang W. S. Chen L. J. Wang Y. Y. Zhu X. F. Liu X. Y. Fan H. Y.. (2020). *Bacillus simplex* treatment promotes soybean defence against soybean cyst nematodes: a metabolomics study using GC-MS. PLoS One 15:e0237194. doi: 10.1371/journal.pone.0237194, PMID: 32760135 PMC7410315

[ref129] Karačić V. Miljaković D. Marinković J. Ignjatov M. Milošević D. Tamindžić G.. (2024). *Bacillus* species: excellent biocontrol agents against tomato diseases. Microorganisms 12:457. doi: 10.3390/microorganisms12030457, PMID: 38543508 PMC10972177

[ref130] Khan A. Chen S. Fatima S. Ahamad L. Siddiqui M. A. (2023). Biotechnological tools to elucidate the mechanism of plant and nematode interactions. Plan. Theory 12:2387. doi: 10.3390/plants12122387, PMID: 37376010 PMC10304871

[ref131] Khan A. R. Mustafa A. Hyder S. Valipour M. Rizvi Z. F. Gondal A. S.. (2022). *Bacillus* spp. as bioagents: uses and application for sustainable agriculture. Biology 11:1763. doi: 10.3390/biology11121763, PMID: 36552272 PMC9775066

[ref132] Khanh T. L. V. (2020). Selection of *Bacillus thuringiensis* against pathogenic nematodes attacking pepper tree. Biotechnology 36, 57–62. doi: 10.21519/0234-2758-2020-36-3-57-62

[ref133] Khullar G. Karami Z. Prakitchaiwattana C. (2024). Development of microencapsulated dried *Bacillus* sp. 63‐11 with enhanced shelf stability and bioactivity for use as a food supplement. Int. J. Food Sci. Technol. 59, 1291–1298. doi: 10.1111/ijfs.16853

[ref134] Kloepper J. W. Ryu C. M. Zhang S. (2004). Induced systemic resistance and promotion of plant growth by *Bacillus* spp. Phytopathology 94, 1259–1266. doi: 10.1094/PHYTO.2004.94.11.1259, PMID: 18944464

[ref135] Köhl J. Kolnaar R. Ravensberg W. J. (2019). Mode of action of microbial biological control agents against plant diseases: relevance beyond efficacy. Front. Plant Sci. 10:845. doi: 10.3389/fpls.2019.00845, PMID: 31379891 PMC6658832

[ref136] Kulkova I. Dobrzyński J. Kowalczyk P. Bełżecki G. Kramkowski K. (2023). Plant growth promotion using *Bacillus cereus*. Int. J. Mol. Sci. 24:9759. doi: 10.3390/ijms24119759, PMID: 37298706 PMC10253305

[ref137] Kumar A. Kakrana A. Sirohi A. Subramaniam K. Srinivasan R. Abdin M. Z.. (2017). Host-delivered RNAi-mediated root-knot nematode resistance in *Arabidopsis* by targeting splicing factor and integrase genes. J. Gen. Plant Pathol. 83, 91–97. doi: 10.1007/s10327-017-0701-3

[ref138] Kumar P. Pandhi S. Mahato D. K. Kamle M. Mishra A. (2021). *Bacillus*-based nano-bioformulations for phytopathogens and insect–pest management. Egypt. J. Biol. Pest Control 31, 1–128. doi: 10.1186/s41938-021-00475-6, PMID: 39743787

[ref139] Lee Y. S. Cho J. Y. Moon J. H. Kim K. Y. (2016). Identification of 2-methylbutyric acid as a Nematicidal metabolite, and biocontrol and biofertilization potentials of *Bacillus pumilus* L1. Korean J. Soil Sci. Fertil. 49, 401–408. doi: 10.7745/KJSSF.2016.49.4.401

[ref140] Li Q. Liu S. Li Y. Hao T. Chen S. (2022). Nitrogen fixation by *Paenibacillus polymyxa* WLY78 is responsible for cucumber growth promotion. Plant Soil 473, 507–516. doi: 10.1007/s11104-022-05307-6, PMID: 39744507

[ref141] Li L. Sun Y. Chen F. Hao D. Tan J. (2023). An alkaline protease from *Bacillus cereus* NJSZ-13 can act as a pathogenicity factor in infection of pinewood nematode. BMC Microbiol. 23:10. doi: 10.1186/s12866-022-02752-2, PMID: 36627592 PMC9830832

[ref142] Li L. Tan J. Chen F. (2018). *Bacillus pumilus* strain LYMC-3 shows nematicidal activity against *Bursaphelenchus xylophilus* via the production of a guanidine compound. Biocontrol Sci. Tech. 28, 1128–1139. doi: 10.1080/09583157.2018.1514587

[ref143] Lilley C. J. Kyndt T. Gheysen G. (2011). “Nematode resistant GM crops in industrialised and developing countries” in Genomics and molecular genetics of plant-nematode interactions (Dordrecht: Springer), 17–541.

[ref144] Lin L. Z. Zheng Q. W. Wei T. Zhang Z. Q. Zhao C. F. Zhong H.. (2020). Isolation and characterization of fengycins produced by *Bacillus amyloliquefaciens* JFL21 and its broad-spectrum antimicrobial potential against multidrug-resistant foodborne pathogens. Front. Microbiol. 11:579621. doi: 10.3389/fmicb.2020.579621, PMID: 33391199 PMC7775374

[ref145] Liu Z. Budiharjo A. Wang P. Shi H. Fang J. Borriss R.. (2013). The highly modified microcin peptide plantazolicin is associated with nematicidal activity of *Bacillus amyloliquefaciens* FZB42. Appl. Microbiol. Biotechnol. 97, 10081–10090. doi: 10.1007/s00253-013-5247-5, PMID: 24085393

[ref146] Liu G. Lin X. Xu S. Liu G. Liu F. Mu W. (2020). Screening, identification and application of soil bacteria with nematicidal activity against root‐knot nematode (*Meloidogyne incognita*) on tomato. Pest Manag. Sci. 76, 2217–2224. doi: 10.1002/ps.5759 31970922

[ref147] Luo L. Zhao C. Wang E. Raza A. Yin C. (2022). *Bacillus amyloliquefaciens* as an excellent agent for biofertilizer and biocontrol in agriculture: an overview for its mechanisms. Microbiol. Res. 259:127016. doi: 10.1016/j.micres.2022.127016, PMID: 35390741

[ref148] Mahapatra S. Chakraborty S. Samanta M. Das S. Islam T. (2022). “Current understanding and future directions of biocontrol of plant diseases by *Bacillus* spp., with special reference to induced systemic resistance” in Bacilli in agrobiotechnology: plant stress tolerance, bioremediation, and bioprospecting (Cham: Springer International Publishing), 127–150.

[ref149] Mahmoud W. M. Abdelmoneim T. S. Elazzazy A. M. (2016). The impact of silver nanoparticles produced by *Bacillus pumilus* as antimicrobial and nematicide. Front. Microbiol. 7:1746. doi: 10.3389/fmicb.2016.01746, PMID: 27891113 PMC5102886

[ref150] Manivannan A. Kumar K. K. Varanavasiappan S. Manimegalai S. Poornima K. Devrajan B. C.. (2019). Expression, purification and bioassay of Cry55Aa protein against tomato root knot nematode, *Meloidogyne incognita*. Res. J. Pharmacogn. Phytochem. 8, 570–573. doi: 10.5958/0975-4385.2020.00004.7

[ref151] Manju P. Subramanian S. (2017). Iturin and Surfactin families of Lipopeptides as key factors in antagonism of *Bacillus subtilis* towards *Meloidogyne incognita* on *Gerbera jamesonii*. Indian J. Nematol. 47, 31–38.

[ref152] Maqsood A. Aslam M. N. Khaliq H. Shakeel M. T. Wu H. Fahad S. (2024). Endophytic *Bacillus* spp. mediated plant growth promotion of tomato seedlings and suppression of *Meloidogyne incognita* and *fusarium oxysporum* disease complex. J. Plant Growth Regul. 43, 2454–2469. doi: 10.1007/s00344-024-11279-x

[ref153] Marin-Bruzos M. Grayston S. J. Forge T. Nelson L. M. (2021). Isolation and characterization of streptomycetes and pseudomonad strains with antagonistic activity against the plant parasitic nematode *Pratylenchus penetrans* and fungi associated with replant disease. Biol. Control 158:104599. doi: 10.1016/j.biocontrol.2021.104599

[ref154] Mathew R. Opperman C. H. (2019). The genome of the migratory nematode, *Radopholus similis*, reveals signatures of close association to the sedentary cyst nematodes. PLoS One 14:e0224391. doi: 10.1371/journal.pone.0224391, PMID: 31652297 PMC6814228

[ref155] Mawarda P. C. Mallon C. A. Le Roux X. Van Elsas J. D. Salles J. F. (2022). Interactions between bacterial inoculants and native soil bacterial community: the case of spore-forming *Bacillus* spp. FEMS Microbiol. Ecol. 98:fiac127. doi: 10.1093/femsec/fiac127, PMID: 36302145 PMC9681130

[ref156] Mazzuchelli R. D. C. L. Mazzuchelli E. H. L. de Araujo F. F. (2020). Efficiency of *Bacillus subtilis* for root-knot and lesion nematodes management in sugarcane. Biol. Control 143:104185. doi: 10.1016/j.biocontrol.2020.104185

[ref157] Mesa-Valle C. M. Garrido-Cardenas J. A. Cebrian-Carmona J. Talavera M. Manzano-Agugliaro F. (2020). Global research on plant nematodes. Agronomy 10:1148. doi: 10.3390/agronomy10081148

[ref158] Messa V. Nunes J. Mattei D. (2019). Seed treatment with *Bacillus amyloliquefaciens* for the control of *Meloidogyne javanica*" *in vivo*" bean culture and its direct effect on the motility, mortality and hatching of *M. javanica* "*in vitro*". Agron. Sci. Biotechnol. 5:59. doi: 10.33158/ASB.2019v5i2p59

[ref159] Mian S. Machado A. C. Z. Hoshino R. T. Mosela M. Higashi A. Y. Shimizu G. D.. (2024). Complete genome sequence of *Bacillus velezensis* strain Ag109, a biocontrol agent against plant-parasitic nematodes and *Sclerotinia sclerotiorum*. BMC Microbiol. 24:194. doi: 10.1186/s12866-024-03282-9, PMID: 38849775 PMC11157790

[ref160] Migunova V. D. Sasanelli N. (2021). Bacteria as biocontrol tool against phytoparasitic nematodes. Plan. Theory 10:389. doi: 10.3390/plants10020389, PMID: 33670522 PMC7922938

[ref161] Migunova V. D. Tomashevich N. S. Konrat A. N. Lychagina S. V. Dubyaga V. M. D’Addabbo T.. (2021). Selection of bacterial strains for control of root-knot disease caused by *Meloidogyne incognita*. Microorganisms 9:1698. doi: 10.3390/microorganisms9081698, PMID: 34442777 PMC8402187

[ref162] Miljaković D. Marinković J. Balešević-Tubić S. (2020). The significance of *Bacillus* spp. in disease suppression and growth promotion of field and vegetable crops. Microorganisms 8:1037. doi: 10.3390/microorganisms8071037, PMID: 32668676 PMC7409232

[ref163] Moens M. Perry R. N. Jones J. T. (2018). “Cyst nematodes - life cycle and economic importance” in Cyst nematodes (Wallingford: CABI), 1–26.

[ref164] Mohamed S. A. El-Sayed G. M. Elkelany U. S. Youssef M. M. El-Nagdi W. M. Soliman G. M. (2021). A local *Bacillus* spp.: isolation, genetic improvement, nematode biocontrol, and nitrogen fixation. Egyptian. Pharm. J. 20, 352–363. doi: 10.4103/epj.epj_30_21, PMID: 36254942

[ref165] Montesinos E. (2003). Development, registration and commercialization of microbial pesticides for plant protection. Int. Microbiol. 6, 245–252. doi: 10.1007/s10123-003-0144-x, PMID: 12955583

[ref166] Moslehi S. Pourmehr S. Shirzad A. Khakvar R. (2021). Potential of some endophytic bacteria in biological control of root-knot nematode *Meloidogyne incognita*. Egypt. J. Biol. Pest Control 31, 1–11. doi: 10.1186/s41938-021-00396-4, PMID: 39743787

[ref167] Mostafa F. A. Khalil A. E. Nour A. Ibrahim D. S. (2018). The role of *Bacillus megaterium* and other bio-agents in controlling root-knot nematodes infecting sugar beet under field conditions. Egypt. J. Biol. Pest Control 28, 1–6. doi: 10.1186/s41938-018-0068-6, PMID: 39743787

[ref168] Nadeem H. Niazi P. Asif M. Kaskavalci G. Ahmad F. (2021). Bacterial strains integrated with surfactin molecules of *Bacillus subtilis* MTCC441 enrich nematocidal activity against *Meloidogyne incognita*. Plant Biol. 23, 1027–1036. doi: 10.1111/plb.13301, PMID: 34263982

[ref169] Ngalimat M. S. Yahaya R. S. R. Baharudin M. M. A. A. Yaminudin S. M. Karim M. Ahmad S. A.. (2021). A review on the biotechnological applications of the operational group *Bacillus amyloliquefaciens*. Microorganisms 9:614. doi: 10.3390/microorganisms9030614, PMID: 33802666 PMC8002464

[ref170] Nguyen V. N. Ju W. T. Kim Y. J. Jung W. J. Kim K. Y. Park R. D. (2014). Suppression of cucumber root-knot nematode Meloidogyne incognita by chitinolytic fungi *Lecanicillium pasalliotae* A-1 and *Lecanicillium antillanum* B-3. J. Chitin Chitos. 19, 93–99.

[ref171] Nguyen D. M. C. Jung W. J. (2014). Nematicidal properties of crude extracts obtained from medicinal plants against root-lesion nematode Pratylenchus coffeae. J. Viet. Environ. 6, 264–269. doi: 10.13141/jve.vol6.no3.pp264-269

[ref172] Nguyen X. H. Naing K. W. Lee Y. S. Jung W. J. Anees M. Kim K. Y. (2013). Antagonistic potential of *Paenibacillus elgii* HOA73 against the root-knot nematode, *Meloidogyne incognita*. Nematology 15, 991–1000. doi: 10.1163/15685411-00002737

[ref173] Nguyen D. M. C. Seo D. J. Kim K. Y. Kim T. H. Jung W. J. (2012). Nematode-antagonistic effects of *Cinnamomum aromaticum* extracts and a purified compound against *Meloidogyne incognita*. Nematology 14, 913–924. doi: 10.1163/156854112X634987

[ref174] Nguyen V. N. Seo D. J. Park R. D. Jung W. J. (2009). Nematicidal activity of compounds extracted from medicinal plants against the pine wood nematode *Bursaphelenchus xylophilus*. Nematology 11, 835–845. doi: 10.1163/156854109X424353

[ref175] Nguyen D. M. C. Seo D. J. Park R. D. Lee B. R. Jung W. J. (2011). Changes in antioxidative enzyme activities in cucumber plants with regard to biological control of root-knot nematode, *Meloidogyne incognita*, with *Cinnamomum cassia* crude extracts. J. Korean Soc. Appl. Biol. Chem. 54, 507–514. doi: 10.3839/jksabc.2011.078

[ref176] Nicol J. M. Turner S. J. Coyne D. L. Nijs L. D. Hockland S. Maafi Z. T. (2011). “Current nematode threats to world agriculture” in Genomics and molecular genetics of plant-nematode interactions (Dordrecht: Springer), 21–43.

[ref177] Niu Q. Huang X. Zhang L. Li Y. Li J. Yang J.. (2006). A neutral protease from *Bacillus nematocida*, another potential virulence factor in the infection against nematodes. Arch. Microbiol. 185, 439–448. doi: 10.1007/s00203-006-0112-x, PMID: 16775750

[ref178] Niu Q. Tian Y. Zhang L. Xu X. E. Niu X. Xia Z.. (2011). Overexpression of the key virulence proteases Bace16 and Bae16 in *Bacillus nematocida* B16 to improve its nematocidal activity. J. Mol. Microbiol. Biotechnol. 21, 130–137. doi: 10.1159/000332805, PMID: 22286040

[ref179] Niu Q. Zhang L. Zhang K. Huang X. Hui F. Kan Y.. (2016). Changes in intestinal microflora of *Caenorhabditis elegans* following *Bacillus nematocida* B16 infection. Sci. Rep. 6:20178. doi: 10.1038/srep20178, PMID: 26830015 PMC4735852

[ref180] O’Callaghan M. (2016). Microbial inoculation of seed for improved crop performance: issues and opportunities. Appl. Microbiol. Biotechnol. 100, 5729–5746. doi: 10.1007/s00253-016-7590-9, PMID: 27188775 PMC4909795

[ref181] Oh I. J. Ju W. T. Kim Y. J. Jung W. J. Kim K. Y. Park R. D. (2014a). Nematicidal activity of *Auxarthron reticulatum* DY-2 against the pine wood nematode *Bursaphelenchus mucronatus*. Nematology 16, 427–436. doi: 10.1163/15685411-00002775

[ref182] Oh I. J. Kim Y. J. Kim K. Y. (2014b). Nematicidal activity of *Verticillium saksenae* A-1 against the pine wood nematode *Bursaphelenchus mucronatus*. J. Chitin Chitos. 19, 81–86.

[ref183] Oka Y. (2010). Mechanisms of nematode suppression by organic soil amendments—a review. Appl. Soil Ecol. 44, 101–115. doi: 10.1016/j.apsoil.2009.11.003

[ref184] Olagoke F. K. Bettermann A. Nguyen P. T. B. Redmile-Gordon M. Babin D. Smalla K.. (2022). Importance of substrate quality and clay content on microbial extracellular polymeric substances production and aggregate stability in soils. Biol. Fertil. Soils 58, 435–457. doi: 10.1007/s00374-022-01632-1

[ref185] Oliveira D. F. Santos H. M. D. Nunes A. S. Campos V. P. Pinho R. S. D. Gajo G. C. (2014). Purification and identification of metabolites produced by *Bacillus cereus* and *B. subtilis* active against *Meloidogyne exigua*, and their in silico interaction with a putative phosphoribosyltransferase from *M. incognita*. An. Acad. Bras. Cienc. 86, 525–538. doi: 10.1590/0001-3765201402412, PMID: 24770454

[ref186] Ortiz A. Sansinenea E. (2023). “Microbial-based biopesticides: commercialization and regulatory perspectives” in Development and commercialization of biopesticides (Cambridge, Massachusetts, USA: Academic Press), 103–118.

[ref187] Osman H. A. Ameen H. H. Mohamed M. Elkelany U. S. (2020). Efficacy of integrated microorganisms in controlling root-knot nematode *Meloidogyne javanica* infecting peanut plants under field conditions. Bull. Natl. Res. Cent. 44, 1–10. doi: 10.1186/s42269-020-00366-0, PMID: 39743787

[ref188] Pacifico M. G. Eckstein B. Bettiol W. (2021). Screening of *Bacillus* for the development of bioprotectants for the control of *Fusarium oxysporum* f. sp. vasinfectum and *Meloidogye incognita*. Biol. Control 164:104764. doi: 10.1016/j.biocontrol.2021.104764, PMID: 39746829

[ref189] Padgham J. L. Sikora R. A. (2007). Biological control potential and modes of action of *Bacillus megaterium* against *Meloidogyne graminicola* on rice. Crop Prot. 26, 971–977. doi: 10.1016/j.cropro.2006.09.004

[ref190] Palomares-Rius J. E. Clavero-Camacho I. Archidona-Yuste A. Cantalapiedra-Navarrete C. León-Ropero G. Braun Miyara S.. (2020). Global distribution of the reniform nematode genus Rotylenchulus with the synonymy of *Rotylenchulus macrosoma* with *Rotylenchulus borealis*. Plan. Theory 10:7. doi: 10.3390/plants10010007, PMID: 33374728 PMC7822487

[ref191] Pandey N. Vaishnav R. Rajavat A. S. Singh A. N. Kumar S. Tripathi R. M.. (2024). Exploring the potential of *Bacillus* for crop productivity and sustainable solution for combating rice false smut disease. Front. Microbiol. 15:1405090. doi: 10.3389/fmicb.2024.1405090, PMID: 38863756 PMC11165134

[ref192] Paradva K. C. Kalla S. (2023). Nanopesticides: a review on current research and future perspective. Chem. Select 8:e202300756. doi: 10.1002/slct.202300756, PMID: 39744071

[ref193] Park M. R. Oh S. Son S. J. Park D. J. Oh S. Kim S. H.. (2015). *Bacillus licheniformis* isolated from traditional Korean food resources enhances the longevity of *Caenorhabditis elegans* through serotonin signaling. J. Agric. Food Chem. 63, 10227–10233. doi: 10.1021/acs.jafc.5b03730, PMID: 26541069

[ref194] Patil G. B. Lakhssassi N. Wan J. Song L. Zhou Z. Klepadlo M.. (2019). Whole‐genome re‐sequencing reveals the impact of the interaction of copy number variants of the rhg1 and Rhg4 genes on broad‐based resistance to soybean cyst nematode. Plant Biotechnol. J. 17, 1595–1611. doi: 10.1111/pbi.13086, PMID: 30688400 PMC6662113

[ref195] Pontes K. B. Machado A. C. Z. Nogueira A. F. Fagundes D. F. V. de Lima Filho R. B. Mosela M.. (2024). Efficacy of microbiological nematicides in controlling root-knot nematodes in tomato. Front. Agron. 6:1462323. doi: 10.3389/fagro.2024.1462323

[ref196] Pradhan P. Naresh P. Barik S. Acharya G. C. Bastia R. (2023). Adamala breeding for root-knot nematode resistance in fruiting Solanaceous vegetable crops: a review. Euphytica 219:71. doi: 10.1007/s10681-023-03204-2

[ref197] Pueyo M. T. Bloch C. Carmona-Ribeiro A. M. Di Mascio P. (2009). Lipopeptides produced by a soil *Bacillus megaterium* strain. Microb. Ecol. 57, 367–378. doi: 10.1007/s00248-008-9464-x, PMID: 18958512

[ref198] Rabbee M. F. Ali M. S. Choi J. Hwang B. S. Jeong S. C. Baek K. H. (2019). *Bacillus velezensis*: a valuable member of bioactive molecules within plant microbiomes. Molecules 24:1046. doi: 10.3390/molecules24061046, PMID: 30884857 PMC6470737

[ref199] Rabbee M. F. Hwang B. S. Baek K. H. (2023). *Bacillus velezensis*: a beneficial biocontrol agent or facultative phytopathogen for sustainable agriculture. Agronomy 13:840. doi: 10.3390/agronomy13030840

[ref200] Radwan M. A. (2007). Efficacy of *Bacillus thuringiensis* integrated with other non-chemical materials to control *Meloidogyne incognita* in tomato. Nematol. Mediterr. 35, 69–73.

[ref201] Ramalakshmi A. Sharmila R. Iniyakumar M. Gomathi V. (2020). Nematicidal activity of native *Bacillus thuringiensis* against the root knot nematode, *Meloidogyne incognita* (Kofoid and white). Egypt. J. Biol. Pest Control 30, 1–9. doi: 10.1186/s41938-020-00293-2, PMID: 39743787

[ref202] Ramezani Moghaddam M. Mahdikhani Moghaddam E. Baghaee Ravari S. Rouhani H. (2014). The first report of *Bacillus pumilus* influence against *Meloidogyne javanica* in Iran. J. Crop Protect. 3, 105–112.

[ref203] Ramírez-Pool J. A. Calderón-Pérez B. Ruiz-Medrano R. Ortiz-Castro R. Xoconostle-Cazares B. (2024). *Bacillus* strains as effective biocontrol agents against Phytopathogenic Bacteria and promoters of plant growth. Microb. Ecol. 87:76. doi: 10.1007/s00248-024-02384-1, PMID: 38801423 PMC11129970

[ref204] Ramyabharathi S. A. Meena K. S. Rajendran L. Raguchander T. Jonathan E. I. (2020). Potential of a rhizobacterium *Bacillus subtilis* (Bbv 57) on *fusarium oxysporum* f. sp. gerberae and *Meloidogyne incognita* infecting Gerbera grown in protected cultivation. Eur. J. Plant Pathol. 158, 615–632. doi: 10.1007/s10658-020-02087-6

[ref205] Rao M. S. Kamalnath M. Umamaheswari R. Rajinikanth R. Prabu P. Priti K.. (2017). *Bacillus subtilis* IIHR BS-2 enriched vermicompost controls root knot nematode and soft rot disease complex in carrot. Sci. Hortic. 218, 56–62. doi: 10.1016/j.scienta.2017.01.051

[ref206] Raymond B. Federici B. A. (2017). In defence of *Bacillus thuringiensis*, the safest and most successful microbial insecticide available to humanity - a response to EFSA. FEMS Microbiol. Ecol. 93. doi: 10.1093/femsec/fix084, PMID: 28645183 PMC5812528

[ref207] Raza A. Hassan A. Akram W. Anjum T. Ali B. (2024). Seed coating with the synthetic consortium of beneficial Bacillus microbes improves seedling growth and manages fusarium wilt disease. Sci. Hortic. 325:112645. doi: 10.1016/j.scienta.2023.112645

[ref208] Riascos-Ortiz D. Mosquera-Espinosa A. T. Varón de Agudelo F. Oliveira C. M. G. Muñoz Flórez J. E. (2022). “Non-conventional management of plant-parasitic nematodes in musaceas crops” in Sustainable management of nematodes in agriculture, Vol. 1: organic management (Cham: Springer International Publishing), 381–422.

[ref209] Riseh R. S. Vatankhah M. Hassanisaadi M. Barka E. A. (2024). Unveiling the role of hydrolytic enzymes from soil biocontrol Bacteria in sustainable Phytopathogen management. Front. Biosci. 29:105. doi: 10.31083/j.fbl2903105, PMID: 38538262

[ref210] Rocha L. F. Duggal P. (2023). “Management of Cyst-Forming Nematodes in agricultural crops through novel biological and genetic engineering technologies” in Novel biological and biotechnological applications in plant nematode management (Singapore: Springer Nature), 313–339.

[ref211] Rostami M. Karegar A. Taghavi S. M. (2021). Biocontrol potential of bacterial isolates from vermicompost and earthworm against the root-knot nematode *Meloidogyne javanica* infecting tomato plants. Egypt. J. Biol. Pest Control 31:36. doi: 10.1186/s41938-021-00383-9

[ref212] Rostami M. Shahbazi S. Soleimani R. Ghorbani A. (2024). Optimizing sustainable control of *Meloidogyne javanica* in tomato plants through gamma radiation-induced mutants of Trichoderma harzianum and *Bacillus velezensis*. Sci. Rep. 14:17774. doi: 10.1038/s41598-024-68365-z, PMID: 39090171 PMC11294331

[ref213] Rozas E. E. Dias M. Acosta A. M. L. Custódio M. R. do C. Mendes M. (2024). Proteomic characterization of metal recovery process realized by marine bacteria *bacillus subtilis* Hyhel1expossed to bioleaching liquor. Braz. J. Chem. Eng. 41, 865–874. doi: 10.1007/s43153-023-00350-x

[ref214] Ruiu L. (2015). Insect pathogenic bacteria in integrated pest management. Insects 6, 352–367. doi: 10.3390/insects6020352, PMID: 26463190 PMC4553484

[ref215] Ryu C. M. Shin J. N. Qi W. Ruhong M. Kim E. J. Pan J. G. (2011). Potential for augmentation of fruit quality by foliar application of bacilli spores on apple tree. Plant Pathol. J. 27, 164–169. doi: 10.5423/PPJ.2011.27.2.164

[ref216] Saeid A. Prochownik E. Dobrowolska-Iwanek J. (2018). Phosphorus solubilization by *Bacillus* species. Molecules 23:2897. doi: 10.3390/molecules23112897, PMID: 30404208 PMC6278551

[ref217] Saikai K. MacGuidwin A. E. (2022). Impact of *Pratylenchus penetrans* on soybean grown in Wisconsin, USA. Plant Dis. 106, 2904–2910. doi: 10.1094/PDIS-09-21-1888-RE, PMID: 35285260

[ref218] Samal I. Bhoi T. K. Mahanta D. K. Komal J. Singh S. (2024). Chapter 3 biorational pest management: potentials, unintended consequences, and future concerns. In: Kumar R. Oliveira M. de Aguiar Andrade E. de Suyal D. Soni R., eds. Biorationals and biopesticides: pest management, Berlin, Boston: De Gruyter 47–76.

[ref219] Sanahuja G. Banakar R. Twyman R. M. Capell T. Christou P. (2011). *Bacillus thuringiensis*: a century of research, development and commercial applications. Plant Biotechnol. J. 9, 283–300. doi: 10.1111/j.1467-7652.2011.00595.x, PMID: 21375687

[ref220] Santos J. Silva A. Queiroz P. Eckstein B. Monnerat R. (2022). Selection of *Bacillus thuringiensis* strains toxic to *Meloidogyne incognita*. Anais Escol. Agron. Veter. 52:e73070. doi: 10.1590/1983-40632022v5273070, PMID: 39699478

[ref221] Saxena A. K. Kumar M. Chakdar H. Anuroopa N. Bagyaraj D. J. (2020). *Bacillus* species in soil as a natural resource for plant health and nutrition. J. Appl. Microbiol. 128, 1583–1594. doi: 10.1111/jam.14506, PMID: 31705597

[ref222] Schnepf E. Crickmore N. Van Rie J. Lereclus D. Baum J. Feitelson J.. (1998). *Bacillus thuringiensis* and its pesticidal crystal proteins. Microbiol. Mol. Biol. Rev. 62, 775–806. doi: 10.1128/mmbr.62.3.775-806.1998, PMID: 9729609 PMC98934

[ref223] Seo D. J. Nguyen V. N. Kim K. Y. Park R. D. Jung W. J. (2013). Nematicidal activity of gallic acid purified from *Terminalia nigrovenulosa* bark against the root-knot nematode *Meloidogyne incognita*. Nematology 15, 507–518. doi: 10.1163/15685411-00002696

[ref224] Serrão C. P. Ortega J. C. G. Rodrigues P. C. de Souza C. R. B. (2024). *Bacillus* species as tools for biocontrol of plant diseases: a meta-analysis of twenty-two years of research, 2000–2021. World J. Microbiol. Biotechnol. 40:110. doi: 10.1007/s11274-024-03935-x, PMID: 38411743

[ref225] Settu V. Annaiyan S. Mannu J. (2024). Revealing the genetic arsenal of *Bacillus firmus* TNAU1: unleashing nematicidal and plant growth promotion traits. Physiol. Mol. Plant Pathol. 129:102177. doi: 10.1016/j.pmpp.2023.102177

[ref226] Shafi J. Tian H. Ji M. (2017). *Bacillus* species as versatile weapons for plant pathogens: a review. Biotechnol. Biotechnol. Equip. 31, 446–459. doi: 10.1080/13102818.2017.1286950

[ref227] Shi J. Peng D. Zhang F. Ruan L. Sun M. (2020). The *Caenorhabditis elegans* CUB-like-domain containing protein RBT-1 functions as a receptor for *Bacillus thuringiensis* Cry6Aa toxin. PLoS Pathog. 16:e1008501. doi: 10.1371/journal.ppat.1008501, PMID: 32369532 PMC7228132

[ref228] Shu J. Zhang R. J. Liang Y. C. Chen Y. Q. Zhang J. Guo J.. (2021). Control of root-knot nematode disease by compounding biological agents from plant and microorganisms. Biotechnol. Bull. 37, 164–174. doi: 10.13560/j.cnki.biotech.bull.1985.2021-0408

[ref229] Sikora R. A. Roberts P. A. (2018). “Management practices: an overview of integrated nematode management technologies,” Plant Parasit. Nemat. Subtrop. Trop. Agric. eds. Sikora R. A. Coyne D. Hallmann J. Timper P. (Wallingford, UK: CABI), *2nd Edition*. 795–838.

[ref230] Singh S. Balodi R. Meena P. N. Singhal S. (2021). Biocontrol activity of Trichoderma harzianum, *Bacillus subtilis* and *Pseudomonas fluorescens* against *Meloidogyne incognita*, *fusarium oxysporum* and *Rhizoctonia solani*. Indian Phytopathol. 74, 703–714. doi: 10.1007/s42360-021-00368-6

[ref231] Singh B. K. Delgado-Baquerizo M. Egidi E. Guirado E. Leach J. E. Liu H.. (2023). Climate change impacts on plant pathogens, food security and paths forward. Nat. Rev. Microbiol. 21, 640–656. doi: 10.1038/s41579-023-00900-7, PMID: 37131070 PMC10153038

[ref232] Singh A. Sharma P. Kumari A. Kumar R. Pathak D. V. (2019). “Management of Root-Knot Nematode in different crops using microorganisms” in Plant biotic interactions. eds. Varma A. Tripathi S. Prasad R. (Cham: Springer), 85–99.

[ref233] Sohrabi F. Sheikholeslami M. Heydari R. Rezaee S. Sharifi R. (2020). Investigating the effect of *Glomus mosseae*, *Bacillus subtilis* and *Trichoderma harzianum* on plant growth and controlling *Meloidogyne javanica* in tomato. Indian Phytopathol. 73, 293–300. doi: 10.1007/s42360-020-00227-w

[ref234] Steinberg N. Keren-Paz A. Hou Q. Doron S. Yanuka-Golub K. Olender T.. (2020). The extracellular matrix protein TasA is a developmental cue that maintains a motile subpopulation within *Bacillus subtilis* biofilms. Sci. Signal. 13:eaaw8905. doi: 10.1126/scisignal.aaw8905, PMID: 32430292

[ref235] Stoica R. M. Moscovici M. I. Ș. U. Tomulescu C. A. T. E. R. I. N. A. Cășărică A. N. G. E. L. A. Băbeanu N. A. R. C. I. S. A. Popa O. V. I. D. I. U.. (2019). Antimicrobial compounds of the genus Bacillus: a review. Rom. Biotechnol. Lett. 24, 1111–1119. doi: 10.25083/rbl/24.6/1111.1119

[ref236] Sturhan D. I. E. T. E. R. Brzeski M. W. (2020). “Stem and bulb nematodes, *Ditylenchus* spp” in Manual of agricultural nematology (Boca Raton, Florida, USA: CRC Press), 423–464.

[ref237] Subbotin S. A. Rius J. E. P. Castillo P. (2021). Systematics of root-knot nematodes (Nematoda: Meloidogynidae): Brill Available at: https://Iccn.loc.gov/2021030916.

[ref238] Sun M. Liang C. Fu X. Liu G. Zhong Y. Wang T.. (2024). Nematocidal activity and biocontrol efficacy of endophytic *Bacillus velezensis* Pt-RP9 from *Pinus tabuliformis* against pine wilt disease caused by *Bursaphelenchus xylophilus*. Biol. Control 196:105579. doi: 10.1016/j.biocontrol.2024.105579, PMID: 39743835

[ref239] Sun X. L. Yang Y. H. Zhu L. Liu F. Y. Xu J. P. Huang X. W.. (2018). The lysine acetylome of the nematocidal bacterium *Bacillus nematocida* and impact of nematode on the acetylome. J. Proteome 177, 31–39. doi: 10.1016/j.jprot.2018.02.005, PMID: 29425737

[ref240] Tian B. Yang J. Zhang K. Q. (2007). Bacteria used in the biological control of plant-parasitic nematodes: populations, mechanisms of action, and future prospects. FEMS Microbiol. Ecol. 61, 197–213. doi: 10.1111/j.1574-6941.2007.00349.x, PMID: 17651135

[ref241] Tian X. L. Zhao X. M. Zhao S. Y. Zhao J. L. Mao Z. C. (2022). The biocontrol functions of *Bacillus velezensis* strain Bv-25 against *Meloidogyne incognita*. Front. Microbiol. 13:843041. doi: 10.3389/fmicb.2022.843041, PMID: 35464938 PMC9022661

[ref242] Timper P. (2014). Conserving and enhancing biological control of nematodes. J. Nematol. 46, 75–89. 24987159 PMC4077175

[ref9002] Tong-Jian X. I. A. O. Fang C. H. E. N. Chao G. A. O. Qing-Yun Z. H. A. O. Qi-Rong S. H. E. N. Wei R. A. N. (2013). Bacillus cereus X5 enhanced bio-organic fertilizers effectively control root-knot nematodes (Meloidogyne sp.). Pedosphere 23, 160–168. doi: 10.1016/S1002-0160(13)60003-X

[ref243] Tran T. P. H. Wang S. L. Nguyen V. B. Tran D. M. Nguyen D. S. Nguyen A. D. (2019). Study of novel endophytic bacteria for biocontrol of black pepper root-knot nematodes in the central highlands of Vietnam. Agronomy 9:714. doi: 10.3390/agronomy9110714

[ref244] Umamaheswari R. Rao M. S. Chaya M. K. Sowmyavani M. Navyashree R. K. Kavya B. M. (2020). Bio-efficacy of liquid formulations of *Bacillus subtilis* IIHR Bs-2 (1% AS) and *Bacillus amyloliquefaciens* IIHR Ba-2 (1% AS) in the management of *Meloidogyne incognita* infecting tomato. Pest Manag. Horticul. Ecosyst. 26, 262–268.

[ref245] Van Frankenhuyzen K. (2009). Insecticidal activity of *Bacillus thuringiensis* crystal proteins. J. Invertebr. Pathol. 101, 1–16. doi: 10.1016/j.jip.2009.02.009, PMID: 19269294

[ref246] Van Frankenhuyzen K. (2013). Cross-order and cross-phylum activity of *Bacillus thuringiensis* pesticidal proteins. J. Invertebr. Pathol. 114, 76–85. doi: 10.1016/j.jip.2013.05.010, PMID: 23747826

[ref247] Vasques N. C. Nogueira M. A. Hungria M. (2024). Increasing application of multifunctional *Bacillus* for biocontrol of pests and diseases and plant growth promotion: lessons from Brazil. Agronomy 14:1654. doi: 10.3390/agronomy14081654

[ref248] Verduzco-Rosas L. A. García-Suárez R. López-Tlacomulco J. J. Ibarra J. E. (2021). Selection and characterization of two *Bacillus thuringiensis* strains showing nematicidal activity against *Caenorhabditis elegans* and Meloidogyne incognita. FEMS Microbiol. Lett. 368:fnaa186. doi: 10.1093/femsle/fnaa186, PMID: 33720297

[ref249] Waller P. J. Thamsborg S. M. (2004). Nematode control in ‘green’ ruminant production systems. Trends Parasitol. 20:493. doi: 10.1016/j.pt.2004.07.012, PMID: 15363444

[ref250] Wang J. Y. Guo C. Zhao P. Yu F. Y. Su Y. Qu J. P.. (2021a). Biocontrol potential of *Bacillus altitudinis* AMCC1040 against root-knot nematode disease of ginger and its impact on rhizosphere microbial community. Biol. Control 158:104598:104598. doi: 10.1016/j.biocontrol.2021.104598

[ref251] Wang J. Y. Zhang X. C. Guo C. Li P. G. Yu F. Y. Zhao P.. (2021b). Diversity and nematocidal activity of culturable bacteria from suppressive soils in Shandong Province, China. Biocontrol Sci. Tech. 31, 387–399. doi: 10.1080/09583157.2020.1854176

[ref252] Wei J. Z. Hale K. Carta L. Platzer E. Wong C. Fang S. C.. (2003). *Bacillus thuringiensis* crystal proteins that target nematodes. Proc. Natl. Acad. Sci. 100, 2760–2765. doi: 10.1073/pnas.0538072100, PMID: 12598644 PMC151414

[ref253] Wepuhkhulu M. Kimenju J. Anyango B. Wachira P. Kyallo G. (2011). Effect of soil fertility management practices and *Bacillus subtilis* on plant parasitic nematodes associated with common bean, *Phaseolus vulgaris*. Trop. Subtrop. Agroecosyst. 13, 27–34.

[ref254] Widianto D. Pramita A. D. Kurniasari I. Arofatullah N. A. Prijambada I. D. Widada J.. (2021). *Bacillus* is one of the most potential genus as a biocontrol agent of golden cyst nematode (*Globodera rostochiensis*). Arch. Phytopathol. Plant Protect. 54, 2191–2205. doi: 10.1080/03235408.2021.1925501

[ref255] Won S. J. Choub V. Kwon J. H. Kim D. H. Ahn Y. S. (2018). The control of fusarium root rot and development of coastal pine (*Pinus thunbergii* Parl.) seedlings in a container nursery by use of *Bacillus licheniformis* MH48. Forests 10:6. doi: 10.3390/f10010006

[ref256] Wu W. Zeng Y. Yan X. Wang Z. Guo L. Zhu Y.. (2023). Volatile organic compounds of *Bacillus velezensis* GJ-7 against *Meloidogyne hapla* through multiple prevention and control modes. Molecules 28:3182. doi: 10.3390/molecules28073182, PMID: 37049944 PMC10096442

[ref257] Xia Y. Xie S. Ma X. Wu H. Wang X. Gao X. (2011). The purL gene of *Bacillus subtilis* is associated with nematicidal activity. FEMS Microbiol. Lett. 322, 99–107. doi: 10.1111/j.1574-6968.2011.02336.x, PMID: 21671997

[ref259] Xiao F. Zhang Y. Zhang L. Li S. Chen W. Shi G.. (2024). Advancing *Bacillus licheniformis* as a superior expression platform through promoter engineering. Microorganisms 12:1693. doi: 10.3390/microorganisms12081693, PMID: 39203534 PMC11356801

[ref260] Xing Z. Wu X. Zhao J. Zhao X. Zhu X. Wang Y.. (2020). Isolation and identification of induced systemic resistance determinants from *Bacillus simplex* Sneb545 against *Heterodera glycines*. Sci. Rep. 10:11586. doi: 10.1038/s41598-020-68548-4, PMID: 32665669 PMC7360772

[ref261] Xiong J. Zhou Q. Luo H. Xia L. Li L. Sun M.. (2015). Systemic nematicidal activity and biocontrol efficacy of *Bacillus firmus* against the root-knot nematode *Meloidogyne incognita*. World J. Microbiol. Biotechnol. 31, 661–667. doi: 10.1007/s11274-015-1820-7, PMID: 25672545

[ref262] Yang J. Liang L. Li J. Zhang K. (2013). Nematicidal enzymes from microorganisms and their applications. Appl. Microbiol. Biotechnol. 97, 7081–7095. doi: 10.1007/s00253-013-5045-0, PMID: 23832084

[ref263] Yang T. Xin Y. Liu T. Li Z. Liu X. Wu Y.. (2022). Bacterial volatile-mediated suppression of root-knot nematode (*Meloidogyne incognita*). Plant Dis. 106, 1358–1365. doi: 10.1094/PDIS-06-21-1139-RE, PMID: 34844448

[ref264] Ye L. Wang J. Y. Liu X. F. Guan Q. Dou N. X. Li J.. (2022). Nematicidal activity of volatile organic compounds produced by *Bacillus altitudinis* AMCC 1040 against *Meloidogyne incognita*. Arch. Microbiol. 204:521. doi: 10.1007/s00203-022-03024-3, PMID: 35879581

[ref265] Yin N. Liu R. Zhao J. L. Khan R. A. A. Li Y. Ling J.. (2021a). Volatile organic compounds of *Bacillus cereus* strain Bc-cm103 exhibit fumigation activity against *Meloidogyne incognita*. Plant Dis. 105, 904–911. doi: 10.1094/PDIS-04-20-0783-RE, PMID: 33135991

[ref266] Yin Y. Wang P. Wang X. Wen J. (2024). Construction of *Bacillus subtilis* for efficient production of fengycin from xylose through CRISPR-Cas9. Front. Microbiol. 14:1342199. doi: 10.3389/fmicb.2023.1342199, PMID: 38249479 PMC10797001

[ref267] Yin N. Zhao J. L. Liu R. Li Y. Ling J. Yang Y. H.. (2021b). Biocontrol efficacy of *Bacillus cereus* strain Bc-cm103 against *Meloidogyne incognita*. Plant Dis. 105, 2061–2070. doi: 10.1094/PDIS-03-20-0648-RE, PMID: 33599517

[ref268] Yu Z. Xiong J. Zhou Q. Luo H. Hu S. Xia L.. (2015). The diverse nematicidal properties and biocontrol efficacy of *Bacillus thuringiensis* Cry6A against the root-knot nematode *Meloidogyne hapla*. J. Invertebr. Pathol. 125, 73–80. doi: 10.1016/j.jip.2014.12.011, PMID: 25556591

[ref269] Yuan Y. Yan Z. Chen Y. Ye J. Tan J. (2023). Effects of *Bacillus cereus* on survival, fecundity, and host adaptability of pine wood nematode. Diversity 15:566. doi: 10.3390/d15040566

[ref270] Yun H. S. Heo J. H. Son S. J. Park M. R. Oh S. Song M. H.. (2014). *Bacillus licheniformis* isolated from Korean traditional food sources enhances the resistance of *Caenorhabditis elegans* to infection by *Staphylococcus aureus*. J. Microbiol. Biotechnol. 24, 1105–1108. doi: 10.4014/jmb.1406.06008, PMID: 24912555

[ref271] Zhang L. N. Jiang C. H. Si F. Song N. Yang W. Zhu Y.. (2024). Long-term field application of a plant growth-promoting rhizobacterial consortium suppressed root-knot disease by shaping the rhizosphere microbiota. Plant Dis. 108, 94–103. doi: 10.1094/PDIS-09-22-2196-RE, PMID: 37467122

[ref272] Zhang J. Li Y. Yuan H. Sun B. Li H. (2016). Biological control of the cereal cyst nematode (*Heterodera filipjevi*) by *Achromobacter xylosoxidans* isolate 09X01 and *Bacillus cereus* isolate 09B18. Biol. Control 92, 1–6. doi: 10.1016/j.biocontrol.2015.08.004

[ref273] Zhang F. Peng D. Ye X. Yu Z. Hu Z. Ruan L.. (2012). *In vitro* uptake of 140 kDa *Bacillus thuringiensis* nematicidal crystal proteins by the second stage juvenile of *Meloidogyne hapla*. PLoS One 7:e38534. doi: 10.1371/journal.pone.0038534, PMID: 22737212 PMC3380895

[ref274] Zhang J. X. Xue A. G. Tambong J. T. (2009). Evaluation of seed and soil treatments with novel *Bacillus subtilis* strains for control of soybean root rot caused by *fusarium oxysporum* and *F. Graminearum*. Plant Dis. 93, 1317–1323. doi: 10.1094/PDIS-93-12-1317, PMID: 30759515

[ref275] Zhaojian G. Qiufen W. Feihong D. Xiang X. Yifeng Z. Wei J.. (2021). Screening and mutagenesis of broad-spectrum antagonistic *Bacillus licheniformis* and purification and identification of antimicrobial substances produced by its mutant. Food Sci. 42, 143–150. doi: 10.7506/spkx1002-6630-20191112-161

[ref276] Zhou Y. Chen J. Zhu X. Wang Y. Liu X. Fan H.. (2021). Efficacy of *Bacillus megaterium* strain Sneb207 against soybean cyst nematode (*Heterodera glycines*) in soybean. Pest Manag. Sci. 77, 568–576. doi: 10.1002/ps.6057, PMID: 32815305

[ref277] Zhu M. Xu X. E. Li Y. Wang P. Niu S. Zhang K.. (2019). Biosynthesis of the nematode attractant 2-Heptanone and its co-evolution between the pathogenic bacterium *Bacillus nematocida* and non-pathogenic bacterium *Bacillus subtilis*. Front. Microbiol. 10:1489. doi: 10.3389/fmicb.2019.01489, PMID: 31312190 PMC6614512

[ref278] Zuckerman B. M. Dicklow M. B. Acosta N. (1993). A strain Of*bacillus thuringiensis* for the control of plant‐parasitic nematodes. Biocontrol Sci. Tech. 3, 41–46. doi: 10.1080/09583159309355257

